# N-MYC impairs innate immune signaling in high-grade serous ovarian carcinoma

**DOI:** 10.1126/sciadv.adj5428

**Published:** 2024-05-15

**Authors:** Alex Miranda, Swetansu Pattnaik, Phineas T. Hamilton, Monica Alvaro Fuss, Shreena Kalaria, Céline M. Laumont, Julian Smazynski, Monica Mesa, Allyson Banville, Xinpei Jiang, Russell Jenkins, Israel Cañadas, Brad H. Nelson

**Affiliations:** ^1^ Deeley Research Centre, BC Cancer, Victoria, BC V8R 6V5, Canada.; ^2^ Department of Medical Genetics, University of British Columbia, Vancouver, BC V6T 1Z3, Canada.; ^3^ The Kinghorn Cancer Centre and Cancer Division, Garvan Institute of Medical Research, 370 Victoria St, Darlinghurst, NSW, Australia.; ^4^ Department of Biochemistry and Microbiology, University of Victoria, Victoria, BC V8P 3E6, Canada.; ^5^ Nuclear Dynamics and Cancer Program, Fox Chase Cancer Center, Philadelphia, PA, USA.; ^6^ Massachusetts General Hospital, Harvard Medical School, Boston, MA, USA.

## Abstract

High-grade serous ovarian cancer (HGSC) is a challenging disease, especially for patients with immunologically “cold” tumors devoid of tumor-infiltrating lymphocytes (TILs). We found that HGSC exhibits among the highest levels of *MYCN* expression and transcriptional signature across human cancers, which is strongly linked to diminished features of antitumor immunity. N-MYC repressed basal and induced IFN type I signaling in HGSC cell lines, leading to decreased chemokine expression and T cell chemoattraction. N-MYC inhibited the induction of IFN type I by suppressing tumor cell–intrinsic STING signaling via reduced STING oligomerization, and by blunting RIG-I–like receptor signaling through inhibition of MAVS aggregation and localization in the mitochondria. Single-cell RNA sequencing of human clinical HGSC samples revealed a strong negative association between cancer cell–intrinsic *MYCN* transcriptional program and type I IFN signaling. Thus, N-MYC inhibits tumor cell–intrinsic innate immune signaling in HGSC, making it a compelling target for immunotherapy of cold tumors.

## Teaser

N-MYC inhibits nucleic acid sensing and interferon signaling in ovarian cancer, making it a compelling target for immunotherapy.

## INTRODUCTION

High-grade serous ovarian carcinoma (HGSC) is the most common form of ovarian cancer, accounting for 70 to 80% of ovarian cancer deaths. Survival rates for HGSC have improved little since the 1980s, highlighting an urgent need for new treatments (*1*). Despite the poor outcomes overall, there is strong evidence that the immune system promotes favorable prognosis in some patients (*2*). Nonetheless, HGSC cancer cells and the tumor microenvironments they engender exhibit numerous immunosuppressive features (*3*), and clinical trials of immunotherapy for this disease have failed to demonstrate substantial benefit (*4*, *5*).

A key process regulating antitumor immunity in many cancer types involves the sensing of cytoplasmic nucleic acids by the cGAS [cyclic guanosine monophosphate–adenosine monophosphate (cGAMP) synthase]/STING [stimulator of interferon (IFN) genes, encoded by *TMEM173*] and RIG-I (retinoic acid inducible gene I, encoded by *DDX58*)–like receptor (RLR) pathways (*6*). Activation of these pathways in tumor cells induces the production of type I IFNs via the serine/threonine kinase TBK1 (TANK-binding kinase 1) and transcription factor IRF3 (IFN regulatory factor 3) (*7*–*11*). Autocrine signaling through IFN type I receptors activates the transcription factor signal transducer and activator of transcription 1 (STAT1), leading to expression of IFN-stimulated genes (ISGs), including multiple immunostimulatory cytokines and chemokines (*12*). Recent evidence demonstrates the impact of cancer cell–intrinsic activation of these pathways on the tumor microenvironment and response to immunotherapies. For example, activation of RIG-I signaling in tumors promotes infiltration of tumor antigen–specific CD8^+^ T cells and response to checkpoint inhibitor–mediated immunotherapy (*7*, *8*). In addition, activation of the STING pathway augments the antigenicity and recognition of human melanoma cells by tumor-infiltrating lymphocytes (TILs), while loss of STING or cGAS in tumor cells decreases T cell infiltration and response to checkpoint blockade in mismatch repair–deficient tumors (*9*–*11*). Furthermore, several studies have shown that suppression of IFN type I production by tumor cells is a fundamental mechanism of immune evasion and resistance to immunotherapy (*13*).

Evidence from other cancers has identified tumor-intrinsic oncogenic signaling pathways that promote not only tumor cell growth but also immune evasion (*14*). Key examples include the β-catenin, phosphatidylinositol 3-kinase (PI3K), and LKB1 (liver kinase B1) pathways, which have been linked to immunosuppression in a variety of cancers (*15*). However, we have only a rudimentary understanding of such mechanisms in HGSC. HGSCs are characterized by near-universal mutation of *TP53*, an intermediate tumor mutation burden, and high frequency of DNA copy number variation (*16*). About half of HGSC cases show evidence of homologous recombination deficiency (HRD) involving BRCA1, BRCA2, or other pathways, and HRD shows a modest positive association with TIL (*17*). In contrast, tumors with genomes characterized by foldback inversions are associated with a colder immune phenotype (*18*, *19*). Frequently mutated or dysregulated oncogenic signaling pathways in HGSC include PI3K, cyclin E, and RAS/mitogen-activated protein kinase (MAPK); however, these alterations have not been linked to immunologically cold tumors (*16*). Intriguingly, Helland *et al.* (*20*) reported dysregulation of the *MYCN* oncogene in the so-called C5/proliferative molecular subtype of HGSC, which is characterized by the lack of immune infiltration. Aberrant activity of N-MYC has been reported in numerous other cancer types, including neuroblastoma, neuroendocrine prostate cancer, breast cancer, glioblastoma multiforme, and small cell lung cancer (*21*), where N-MYC has been implicated in driving tumorigenesis, leading to poor prognosis. In bioinformatic studies of neuroblastoma, *MYCN* gene amplification and the ensuing dysregulation of N-MYC function have been linked to a colder tumor phenotype (*22*, *23*), lending further support to the notion that N-MYC activity may impair antitumor immunity.

Here, we report that HGSC exhibits among the highest levels of *MYCN* expression and activity in human cancer, and this is strongly linked to diminished IFN type I signaling and antitumor immunity. By inducibly controlling *MYCN* expression in human HGSC cell lines, we show that N-MYC inhibits basal and induced IFN type I signaling, leading to suppression of numerous downstream genes and T cell chemoattraction. We further show in vitro that N-MYC suppresses STING and RLR signaling in a tumor cell–intrinsic fashion by inhibiting oligomerization of STING and MAVS (mitochondrial antiviral-signaling protein) independent of transcriptional repression. Single-cell RNA sequencing (scRNA-seq) of clinical HGSC samples validated the cancer cell–intrinsic connection between the *MYCN* gene signature and suppression of IFN type I signaling. Thus, N-MYC emerges as an important regulator of tumor cell–intrinsic immune signaling in HGSC, making this pathway a compelling target to enhance immune control in this challenging malignancy.

## RESULTS

### *MYCN* is inversely associated with antitumor immunity in HGSC

**Fig. 1.**
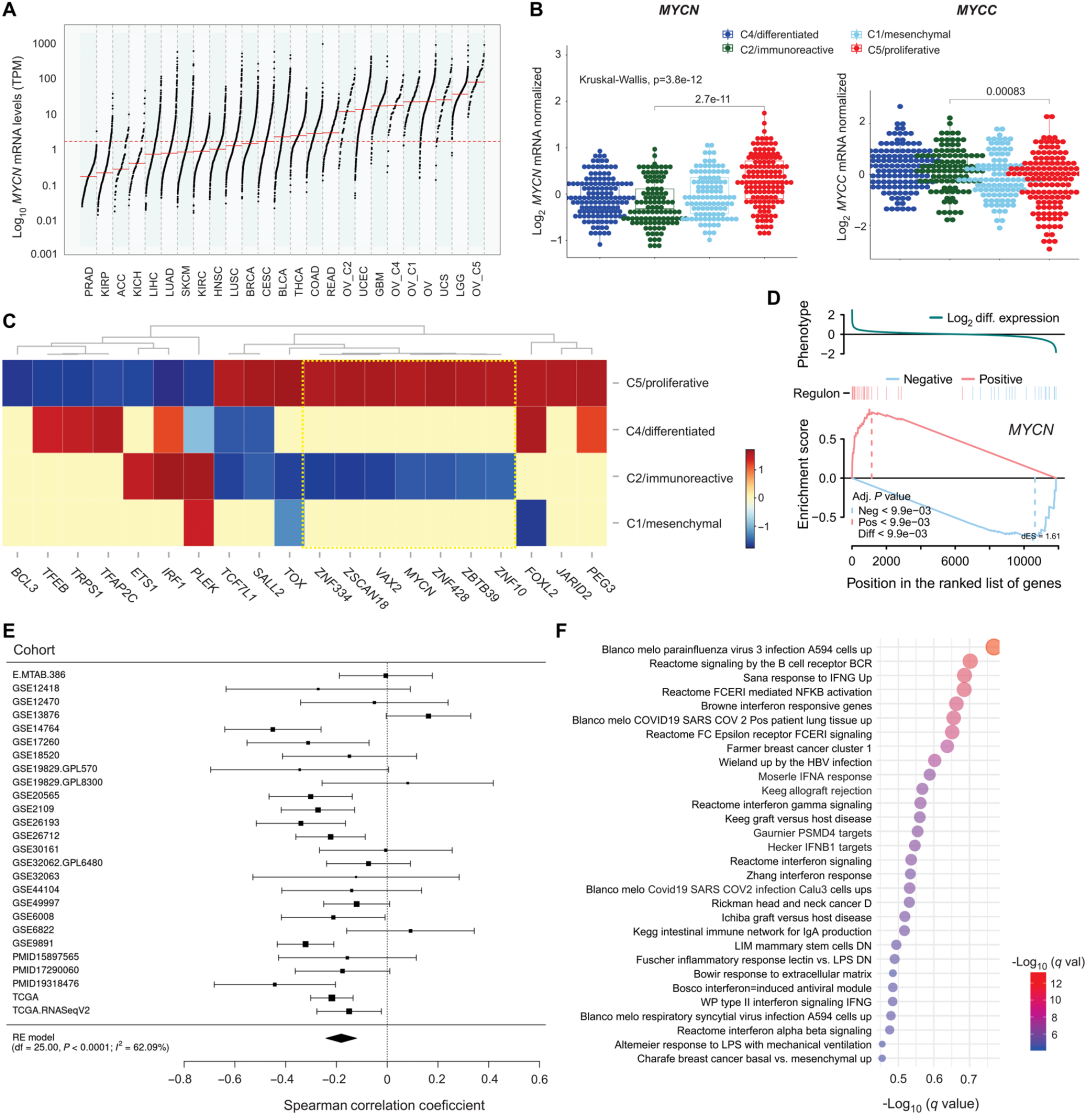
*MYCN* is highly expressed in HGSC and negatively associated with features of antitumor immunity. (**A**) *MYCN* gene expression across solid cancers and the four molecular subtypes of HGSC (C1, C2, C4, and C5) from TCGA. Each point represents an individual case. The dashed line represents median *MYCN* score across all 21 TCGA cancers. (**B**) Box plots depict expression of *MYCN* and *MYCC* genes across the four molecular subtypes of HGSC. Microarray gene expression data for 486 patients with HGSC were obtained from TCGA. Kruskal-Wallis with Dunn posttest for pairwise comparison. (**C**) Hierarchical clustering analysis of two-tailed Gene Set Enrichment Analysis (GSEA) enrichment scores. Enrichment scores are shown for the top 20 differentially enriched regulons in the proliferative molecular subtype. Two-tailed GSEA was used to assess whether transcriptional targets of a transcription factor were statistically enriched in genes differentially expressed between the proliferative subtype and all others. The ARACNE inferred regulons are treated as gene sets in this analysis. The observed enrichment score, the extreme values color coded as red, and blue indicate positive and negative correspondence with phenotype. (**D**) Two-tailed GSEA plot for N-MYC regulon. N-MYC regulon is split into positive and negative targets, and differential enrichment score (dES) is shown for positive (red line) and negative (blue line) targets. (**E**) Forest plot depicting correlation of *MYCN* mRNA expression with the immune cytolytic score [geometric mean *GZMA*, *PRF1* (*27*)] on multiple ovarian cohorts (*26*). Correlation analysis was performed using Spearman’s rank method. (**F**) Gene signatures that are significantly up-regulated in low-expressing *MYCN* tumors. HGSC samples were rank-ordered using *MYCN* expression values, and the top 0.3 quantiles (higher than 70th percentile, 125 cases) and bottom 0.3 quantiles (lower than 30th percentile, 124 cases) were used for GAGE analysis. Pathway gene sets contained in the MSigDb (C2 gene sets) database were used.

An analysis of RNA-seq data from 8290 primary tumors representing 21 solid cancer types from TCGA (The Cancer Genome Atlas) revealed that *MYCN* mRNA expression varied widely within and across cancer types. In agreement with prior work (*24*), *MYCN* expression was highest in low-grade glioma and glioblastoma. HGSC showed among the highest expression of *MYCN*, third only to low-grade glioma and uterine carcinosarcoma (Fig. 1A). As previously reported (*20*), higher expression of *MYCN* was observed in the C5/proliferative (immunologically cold) subtype compared to the C2/immunoreactive (immunologically hot) subtype (Fig. 1, A and B). The C5/proliferative subtype showed the highest *MYCN* expression of all cancer types in TCGA (Fig. 1A). Most HGSC tumors, including a large proportion of C2/immunoreactive tumors, exhibited *MYCN* expression above the median of the TCGA cohort. Compared with ovary, where metastases from HGSC are commonly found, HGSC tumors showed substantially higher *MYCN* expression (fig. S1). *MYCC* isoform expression showed the inverse pattern, with the highest expression seen in the C2/immunoreactive subtype (Fig. 1B). To further investigate the relationship between *MYCN* expression and HGSC molecular subtype, we inferred global transcription networks in the TCGA dataset by applying ARACNe (*25*) to a FANTOM5-derived list of 1672 transcription factors. This revealed that the N-MYC regulon was among the top 10 differentially activated regulons in the C5/proliferative compared to the C2/immunoreactive subtype (Fig. 1C and data S1). Furthermore, Gene Set Enrichment Analysis 2 (GSEA^2^) revealed concurrent up-regulation of N-MYC–induced targets and down-regulation of N-MYC–repressed targets in C5/proliferative tumors relative to other molecular subtypes (*P* < 0.001) (Fig. 1D).

We also assessed *MYCN* expression across different cancer cell lines using data from the Cancer Cell Line Encyclopedia (CCLE). In contrast to the elevated *MYCN* expression seen in HGSC clinical samples, the human ovarian and HGSC cell lines in CCLE exhibited relatively low expression and gene amplification of *MYCN* (fig. S2, A and B). This suggests that *MYCN*-amplified tumors may not be amenable to cell line derivation and/or that the high *MYCN* expression seen in clinical samples reflects extrinsic regulatory mechanisms that manifest in C5/proliferative tumors.

Returning to clinical datasets, we investigated the relationship between *MYCN* and antitumor immunity in the curated ovarian cancer database, which combines data from TCGA and other independent cohorts (*26*). Meta-analysis across different studies revealed a negative association between *MYCN* mRNA expression and immune cytolytic score (Fig. 1E) (*27*). Likewise, a negative association was seen between a *MYCN* HGSC–derived signature (described below) and immune cytolytic score (fig. S3A). Furthermore, to provide an unbiased survey of processes that negatively correlate with *MYCN* expression, we performed GAGE (generally applicable gene set enrichment) analysis in the TCGA cohort, comparing samples with higher than 70th percentile versus lower than 30th percentile *MYCN* mRNA expression. Recognizing that the presence of nonmalignant cells can confound expression analyses of bulk-sequenced tumor samples by diluting tumor-specific expression signatures, we used tumor purity–corrected expression data for this analysis (*28*). Almost all of the pathways enriched in low-*MYCN* samples were immune related, including multiple IFN signatures (Fig. 1F and data S2). Similar results were obtained using a *MYCN* HGSC–derived signature score (described below) (fig. S3B). Collectively, these analyses demonstrate a negative association between signatures of *MYCN* expression/activity and antitumor immunity in HGSC.

### N-MYC represses basal IFN-regulated genes and T cell chemoattraction

**Fig. 2.**
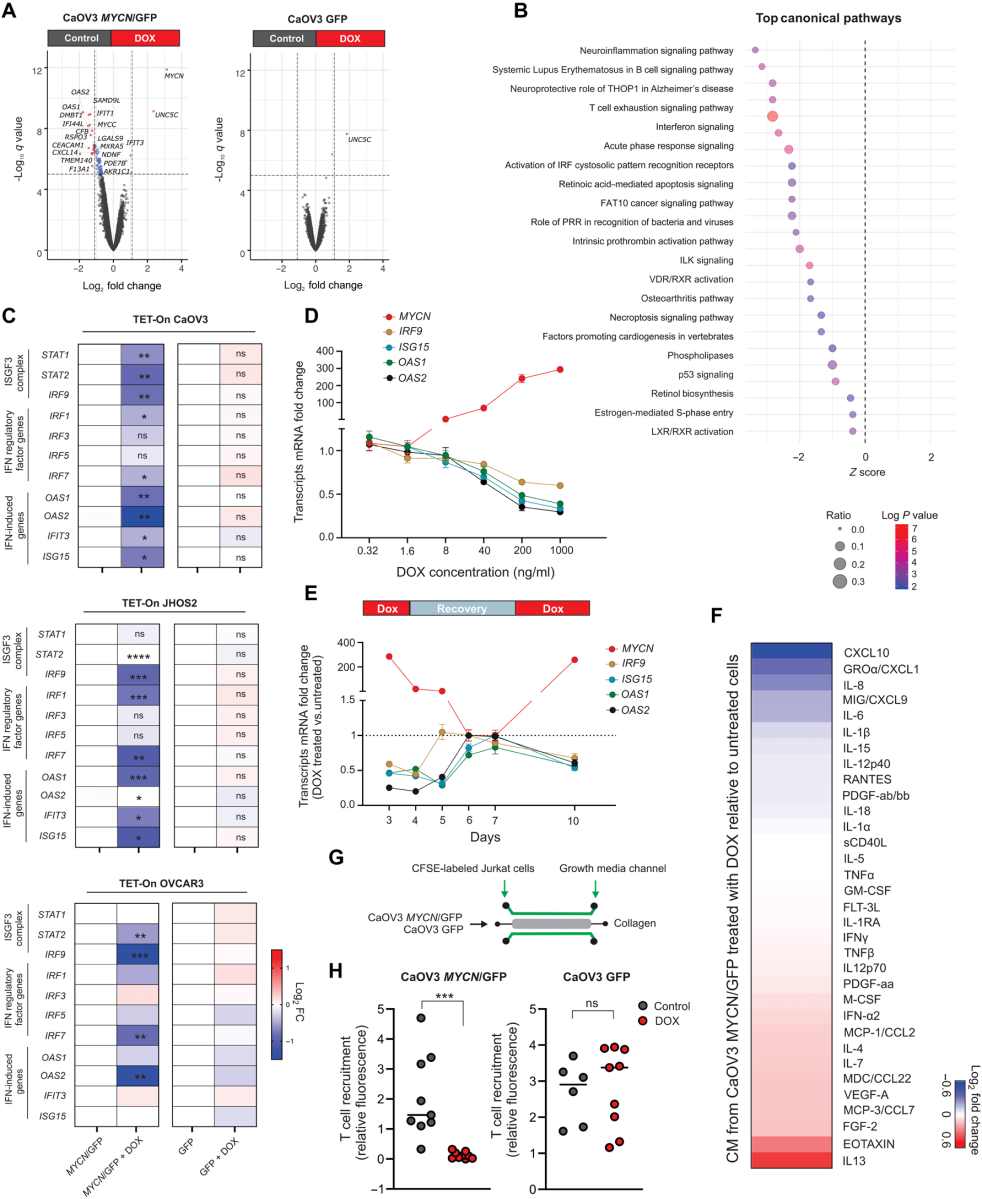
N-MYC represses basal IFN-regulated genes and T cell chemoattraction in HGSC. (**A**) Volcano plots depicting differentially expressed genes on CaOV3 *MYCN*/green fluorescent protein (GFP) (left) and GFP (right) TET-On cells treated ± DOX (1 μg/ml). *X* axis represents log_2_ fold change of gene expression (treated/untreated), and *Y* axis represents 1 × log_10_ false discovery rate (FDR) *q* value for each gene. The dashed line indicates FDR *q* value = 0.05. (**B**) Canonical Ingenuity pathway analysis (IPA) on differentially expressed genes (*P*_adj_ < 0.05) of CaOV3 *MYCN*/GFP cells treated ± DOX (1 μg/ml). (**C**) Heatmaps showing the change in expression of IFN-induced genes (ISGs), IFN regulatory factors, and genes encoding the ISGF3 signaling complex, in CaOV3 *MYCN*/GFP cells (left) or GFP (right) treated with DOX. Red-blue intensities reflect the fold changes determined by qRT-PCR. ns, nonsignificant. (**D**) qRT-PCR evaluation of ISGs and *MYCN* in CaOV3 *MYCN*/GFP cells treated with the indicated doses of DOX. (**E**) qRT-PCR evaluation of ISGs and *MYCN* in CaOV3 *MYCN*/GFP cells treated with DOX (1 μg/ml) for 24, 48, and 72 hours; a washout/recovery period in media without DOX; and a second stimulation with DOX (1 μg/ml) for another 72 hours. (**F**) Changes (log_2_ fold) in cytokine and chemokine concentrations in media from CaOV3 *MYCN*/GFP cells treated with DOX relative to media alone. (**G**) Schematics of coculture of CaOV3 *MYCN*/GFP and GFP cells with CFSE-labeled Jurkat T cells. (**H**) Quantification of fluorescence intensity of migrated CFSE-labeled Jurkat cells in collagen with CaOV3 *MYCN*/GFP and GFP cells pretreated ± DOX. Data are representative of two independent experiments. Data in (C) to (F) are means ± SEM of *n* = 3 biological replicates. *P < 0.05; **P < 0.005; ***P < 0.001; ****P < 0.0001. All *P* values were calculated using an unpaired two-tailed Student’s *t* test.

To create an experimental system to investigate the molecular events associated with N-MYC activity in HGSC, we generated *MYCN* Tetracycline-On (TET-On) models in three independent human HGSC cell lines (CaOV3, JHOS-2, and NIH:OVCAR3), which, as described above, expressed negligible levels of *MYCN* at baseline (fig. S2A). We achieved tight, dose-dependent control of N-MYC levels with low background, and N-MYC was preferentially localized in the nuclear fraction as expected (fig. S4, A to D, and Fig. 2D). N-MYC induction did not affect cell proliferation or migration; however, it increased anchorage-independent growth (fig. S5, A to C), in line with its described oncogenic functions (*21*). To define the N-MYC transcriptional program, we profiled the transcriptome of CaOV3 *MYCN* TET-On cells treated with doxycycline (DOX) compared to untreated controls. As confirmation of successful induction, *MYCN* was the top up-regulated gene after DOX treatment (Fig. 2A). We identified 519 others differentially expressed genes (*P*_adj_ < 0.05), of which the vast majority (87.7%) were down-regulated in DOX-treated cells. *MYCC* was identified among the top down-regulated genes, and further experiments demonstrated a time-dependent reduction of c-MYC protein levels after DOX treatment (fig. S6, A and B). Canonical Ingenuity pathway analysis on differentially expressed genes identified “IFN signaling” as the top down-regulated network (*P* < 10^−8^) (Fig. 2B). Other immune-related networks were also repressed, including “Acute Phase Response” and “Activation of IRF by cytosolic Pattern recognition receptors” (Fig. 2B and data S3).

Quantitative polymerase chain reaction (qPCR)–based evaluation of individual genes in multiple *MYCN* TET-On HGSC cell lines revealed consistent DOX-induced repression of ISGs, IRFs, and genes encoding the ISGF3 signaling complex (Fig. 2C). Exposure of cells to increasing DOX concentrations led to dose-dependent repression of the ISGs *OAS2*, *ISG15*, *OAS1*, and *IRF9* (Fig. 2D). Withdrawal of DOX resulted in complete loss of *MYCN* mRNA expression and recovery of ISG expression after 48 to 72 hours, demonstrating the reversibility of N-MYC–mediated suppression of the basal IFN-regulated gene program. Furthermore, redosing with DOX restored *MYCN* expression and repression of ISG expression (Fig. 2E).

We next explored the impact of N-MYC on the secretion of IFN-regulated chemokines. Following DOX treatment, multiplexed cytokine/chemokine profiling identified decreased levels of CXCL8 [interleukin-8 (IL-8)], CXCL1, and the T helper 1 (T_H_1)–associated chemokines CXCL9 and CXCL10. Conversely, the T_H_2 chemokines Eotaxin and IL-13 were increased (Fig. 2F). These changes in chemokine secretion were associated with reduced recruitment of Jurkat T cells in three-dimensional (3D) microfluidic coculture with DOX-stimulated CaOV3 *MYCN* TET-On cells (Fig. 2, G and H). In other coculture experiments, *MYCN* induction did not affect the proliferation of healthy donor–derived peripheral blood T cells (fig. S7, A and B). Thus, N-MYC suppresses the expression of basal IFN-regulated genes and chemokines in tumor cells, with a concomitant reduction in T cell chemoattraction.

### N-MYC represses IFN type I synthesis and signaling

**Fig. 3.**
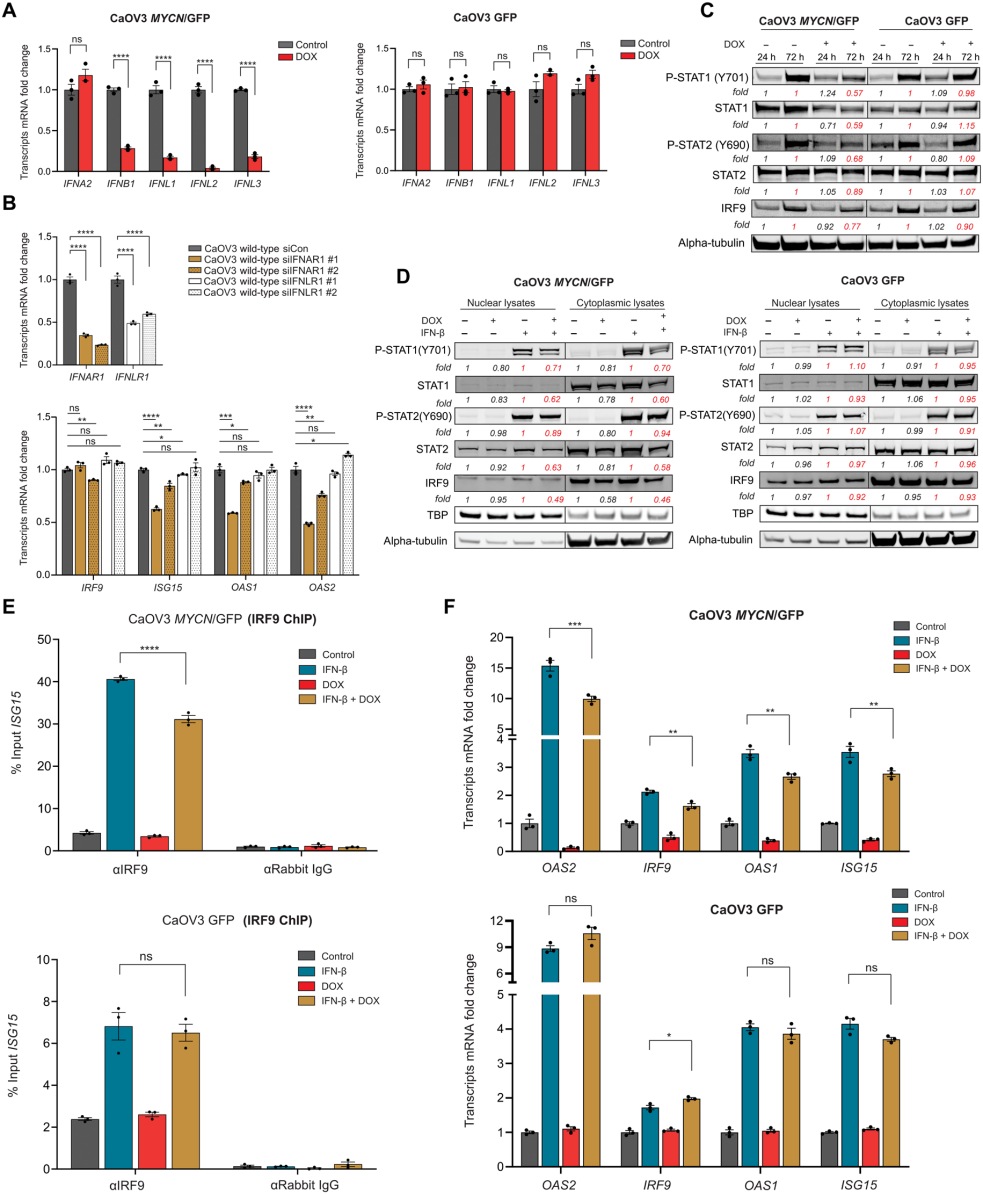
N-MYC represses IFN type I immune signaling. (**A**) qRT-PCR of IFN type I (*IFNA2* and *IFNB1*) and III (*IFNL1*, *IFNL2*, and *IFNL3*) ligands in CaOV3 *MYCN*/GFP and GFP cells treated ± DOX (1 μg/ml) for 72 hours. *P* values were calculated using an unpaired two-tailed Student’s *t* test. (**B**) qRT-PCR of *IFNAR1* and *IFNLR1* (top) and ISGs (bottom) in CaOV3 wild-type cells transfected with scrambled negative control siRNA or siRNAs specific for *IFNAR1* and *IFNLR1* for 72 hours. (**C**) Immunoblot of P-STAT1, STAT1, P-STAT2, STAT2, IRF9, and α-tubulin levels in CaOV3 *MYCN*/GFP and CaOV3 GFP cells treated ± DOX for 24 and 72 hours. Data are representative of three independent experiments. Paired comparisons are shown in the same color for densitometry fold changes. (**D**) Immunoblot with the indicated antibodies in CaOV3 *MYCN*/GFP and GFP cells pretreated ± DOX for 72 hours and then treated with IFNB1 (10 ng/ml) for 2 hours. Nuclear and cytoplasmic fractions were prepared and subjected to Western blot. Data are representative of three independent experiments. (**E**) ChIP-qPCR analysis of IRF9 binding to the ISRE sequence of the *ISG15* promoter in CaOV3 *MYCN*/GFP and GFP cells pretreated ± DOX for 72 hours and then treated with IFNB1 (50 ng/ml) for 30 min. *P* values were calculated using one-way analysis of variance (ANOVA) with Tukey posttest for pairwise comparison. (**F**) qRT-PCR of multiple ISGs in CaOV3 *MYCN*/GFP and GFP cells pretreated ± DOX (1 μg/ml) for 72 hours followed by IFNB1 pulse (10 ng/ml) for 2 hours and then 24-hour chase. *P* values were calculated using one-way ANOVA with Tukey posttest for pairwise comparison. Data in (A), (B), (E), and (F) are means ± SEM of *n* = 3 biological replicates. *P < 0.05; **P < 0.005; ***P < 0.001; ****P < 0.0001. Densitometry analysis was performed using ImageJ software.

To explain the transcriptional repression of ISGs by N-MYC, we hypothesized that N-MYC might inhibit basal IFN type I synthesis and/or signaling. After DOX treatment of CaOV3 *MYCN* TET-On cell lines, we observed reduced mRNA expression of *IFNB1*, as well as the type III IFNs *IFNL1*, *IFNL2*, and *IFNL3* (Fig. 3A). While N-MYC can repress both IFN type I and III ligands, small interfering RNA (siRNA) and antibody blocking experiments demonstrated higher dependency on *IFNAR1* (cognate receptor for type I IFN) for the basal expression of ISGs (Fig. 3B and fig. S8A). This finding corresponded with higher expression of *IFNAR1* compared to *IFNLR1* in 17 independent HGSC cell lines, in multiple ovarian cohorts, and in primary HGSC cancer cells analyzed by scRNA-seq (fig. S8, B to D).

To assess the impact of N-MYC on basal IFN-mediated signal transduction, we cultured CaOV3 *MYCN* TET-On cells for 24 or 72 hours in the presence or absence of DOX. At 72 hours, the basal protein levels of STAT1, STAT2, and IRF9, as well as the tyrosine phosphorylation of STAT1 and STAT2, were decreased in cells cultured with DOX versus without DOX, consistent with N-MYC–induced inhibition of basal IFN signaling (Fig. 3B).

We then evaluated the effect of N-MYC on the response to exogenous type I IFN. Binding of IFN to IFNAR activates Janus kinase (JAK) family kinases, which in turn leads to tyrosine phosphorylation of STAT1. STAT1 then assembles with STAT2 and IRF9 to form the ISGF3 complex, which translocates to the nucleus and drives expression of ISGs (*29*). By treating CaOV3, JHOS-2, and NIH:OVCAR3 *MYCN* TET-On cells with IFNB1 in the presence or absence of DOX, we found that N-MYC decreased total and phosphorylated levels of STAT1 (both cytoplasmic and nuclear), as well as nuclear IRF9 (Fig. 3C and fig. S9, A and B). IRF9 is the DNA binding subunit of the ISGF3 complex and has a well-established role in the induction of ISRE (IFN-sensitive response element)–associated ISGs (*29*). Therefore, we used chromatin immunoprecipitation (ChIP)–qPCR to evaluate the impact of N-MYC on the binding of IRF9 to *ISG15* ISRE-promoter sequences. Consistent with the above results, DOX treatment led to decreased IRF9 binding in the presence of exogenous IFNB1 (Fig. 3D and fig. S9, C and D), along with reduced expression of ISGs assessed by qPCR (Fig. 3E). Thus, N-MYC suppresses basal and IFN-stimulated signaling events and target gene expression.

### N-MYC inhibits RLR signaling pathway by suppressing MAVS aggregation and localization in mitochondria

**Fig. 4.**
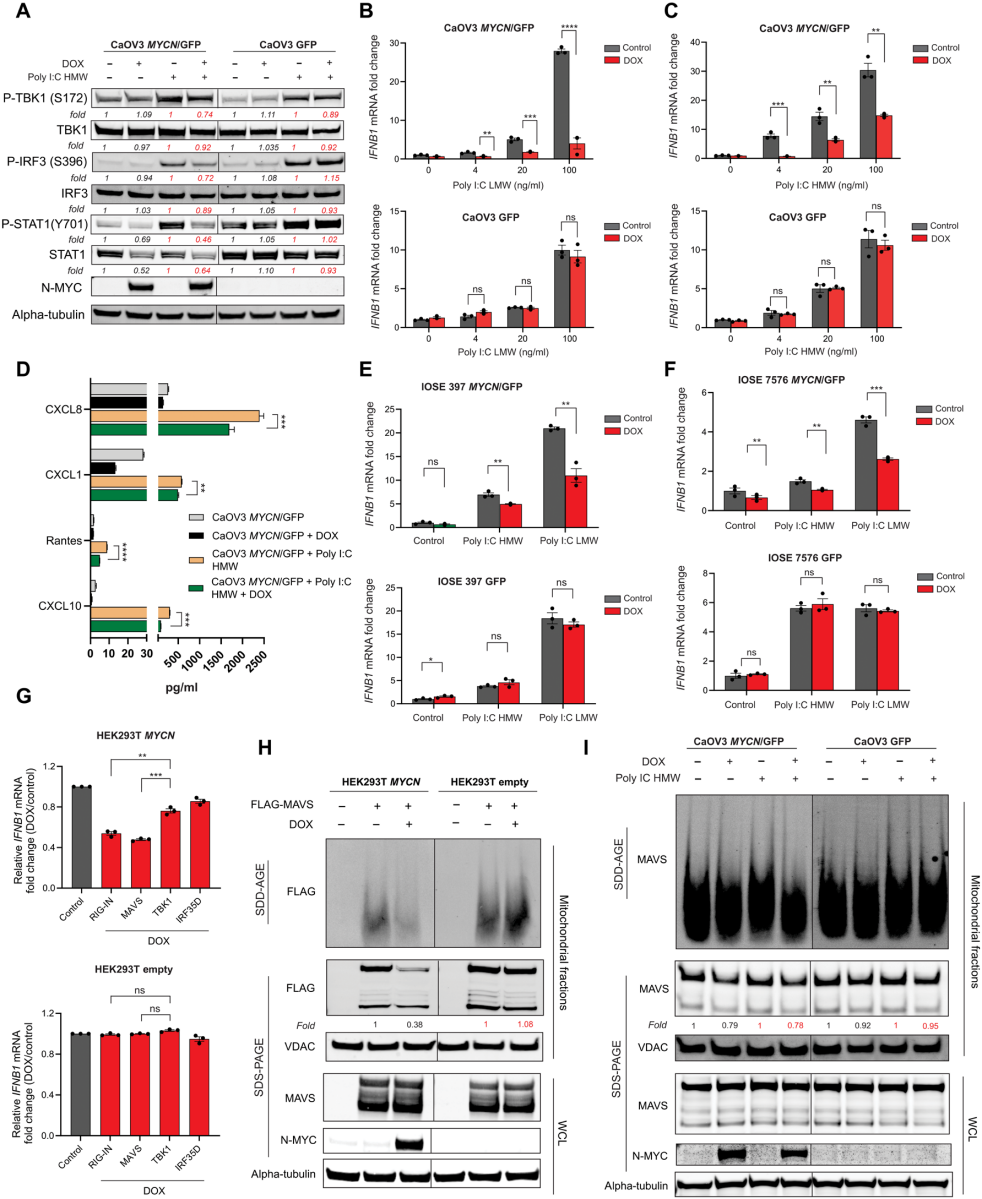
N-MYC inhibits response to dsRNA by suppressing MAVS aggregation and localization in the mitochondria. (**A**) Immunoblot with the indicated antibodies against lysates from CaOV3 *MYCN*/GFP and GFP cells pretreated ± DOX and transfected ± Poly I:C HMW (500 ng/ml). Data are representative of three independent experiments. (**B** and **C**) qRT-PCR of *IFNB1* in CaOV3 *MYCN*/GFP and GFP cells pretreated ± DOX and transfected with the indicated concentrations of Poly I:C LMW (B) and Poly I:C HMW (C). (**D**) Luminex data of human CXCL8, CXCL1, RANTES, and CXCL10 in supernatants from CaOV3 *MYCN*/GFP cells treated ± DOX and stimulated ± Poly I:C HMW (100 ng/ml). (**E** and **F**) qRT-PCR of *IFNB1* in IOSE-397 (E) and IOSE-7576 (F) *MYCN*/GFP and GFP cells pretreated ± DOX and transfected ± Poly I:C HMW or Poly I:C LMW (100 ng/ml). (**G**) qRT-PCR of *IFNB1* expression in HEK293T *MYCN* and empty vector cells pretreated ± DOX and transfected with the indicated plasmids. (**H**) Immunoblots showing expression/aggregation of MAVS in HEK293T *MYCN* and empty vector cells that were pretreated ± DOX and transfected with pFLAG-MAVS. Mitochondrial fractions and whole-cell lysates were run by SDD-AGE or SDS-PAGE and immunoblotted with the indicated antibodies. Data are representative of three independent experiments. (**I**) Immunoblots showing expression/aggregation of MAVS in CaOV3 *MYCN*/GFP and GFP cells that were pretreated ± DOX and transfected ± Poly I:C HMW (500 ng/ml). Mitochondrial fractions and whole-cell lysates were run by SDD-AGE or SDS-PAGE and analyzed by immunoblots with the indicated antibodies. Data are representative of the independent experiments. All *P* values were calculated using an unpaired two-tailed Student’s *t* test. Data in (A), (B), and (D) to (G) are means ± SEM of *n* = 3 biological replicates. *P < 0.05; **P < 0.005; ***P < 0.001; ****P < 0.0001. Densitometry analysis was performed using ImageJ software.

The transcription of basal type I IFNs is typically regulated by double-stranded DNA (dsDNA) and RNA (dsRNA) nucleic acid sensing pathways (*6*). RIG-I and MDA-5 are the two major dsRNA sensors that defend against viruses and other pathogens. Upon detection of cytoplasmic dsRNA, RIG-I and MDA-5 associate with MAVS, leading to the recruitment of TBK1 and IRF3, which in turn triggers the expression of type I IFNs and T cell chemokines (*30*). To evaluate the effect of N-MYC on dsRNA sensing, we transfected CaOV3 *MYCN* TET-On cells with the dsRNA mimic Polyinosinic:polycytidylic acid (Poly I:C) high molecular weight (HMW; which preferentially activates MDA-5) and Poly I:C low molecular weight (LMW; which preferentially activates RIG-I). Strikingly, DOX treatment inhibited dsRNA-induced events, including phosphorylation of TBK1, IRF3, and STAT1 (Fig. 4A); expression of mRNAs encoding *IFNB1*, *IFNL1*, and *IFNL2* (Fig. 4, B and C, and fig. S10, A and B); and secretion of the chemokines CXCL10, RANTES, CXCL8, and CXCL1 (Fig. 4D). Similar results were observed using *MYCN* TET-On models derived from the immortalized but nonmalignant ovarian surface epithelial cell lines IOSE-397 and IOSE-7576 (*31*) (fig. S11, A to D, and Fig. 4, E and F), demonstrating that these effects of N-MYC are not unique to fully transformed cancer cells.

To investigate the mechanism by which N-MYC inhibits the dsRNA sensing pathway, we turned to human embryonic kidney (HEK) 293T cells, which are highly amenable to transient transfection and have been used extensively as a model system for nucleic acid sensing. We generated an HEK293T *MYCN* TET-On model (fig. S12, A to C) and, as expected, found that DOX-induced N-MYC expression inhibited the induction of *IFNB1* mRNA in response to Poly I:C HMW and Poly I:C LMW (fig. S12D). To evaluate the step(s) of the pathway affected by N-MYC, HEK293T *MYCN* TET-On cells were transfected separately with plasmids encoding RIG-IN [the N terminus 2 caspase activation and recruitment domain (2CARD) of RIG-I], MAVS, TBK1, or IRF3-5D (an active form of IRF3). As expected, each of these constructs induced *IFNB1* mRNA expression in the absence of DOX, but DOX treatment preferentially inhibited *IFNB1* induction by RIG-IN and MAVS compared to TBK1 and IRF3 (Fig. 4G), suggesting that N-MYC acts just upstream of TBK1 and IRF3.

RNA binding by RIG-I or MDA-5 induces aggregation of MAVS on the mitochondrial membrane, which then leads to activation of TBK1, phosphorylation of IRF3, and the expression of type 1 IFNs (*32*). These events can be mimicked by overexpressing MAVS in cells (*32*), providing a convenient experimental system to examine the effect of N-MYC on MAVS aggregation. To this end, HEK293T *MYCN* TET-On cells were transfected with FLAG-tagged MAVS, and isolated mitochondrial fractions were subjected to semi-denaturing detergent agarose gel electrophoresis (SDD-AGE). MAVS aggregation was evident in mitochondrial fractions from control cells but was notably reduced by DOX-induced expression of N-MYC (Fig. 4H). Furthermore, in the CaOV3 *MYCN* TET-On model, Poly I:C–induced aggregation of endogenous MAVS was inhibited by DOX treatment (Fig. 4I). MAVS aggregation has been shown to involve K63-linked polyubiquitination (*33*), and we detected K63-linked ubiquitination of MAVS in HEK293T *MYCN* TET-On cells in the absence of DOX (fig. S13). However, this was only marginally reduced by DOX treatment, suggesting that N-MYC suppresses MAVS aggregation through another mechanism. Collectively, these data demonstrate that N-MYC suppresses dsRNA sensing by inhibiting MAVS localization and aggregation in mitochondria.

### N-MYC increases cytoplasmic dsDNA while inhibiting the cGAS/STING signaling pathway

Whereas dsRNA sensing is mediated through MAVS, dsDNA sensing depends on the cGAS/STING pathway (fig. S14A). To define the differential contributions of these pathways to basal IFN expression, we individually knocked down the genes encoding MAVS and STING and, as a control, MYD88 (which is essential for Toll-like receptor signaling but not nucleic acid sensing). Knockdown of STING led to the greatest repression of basal ISG (fig. S14B), corroborating data from other cancer models (*34*).

**Fig. 5.**
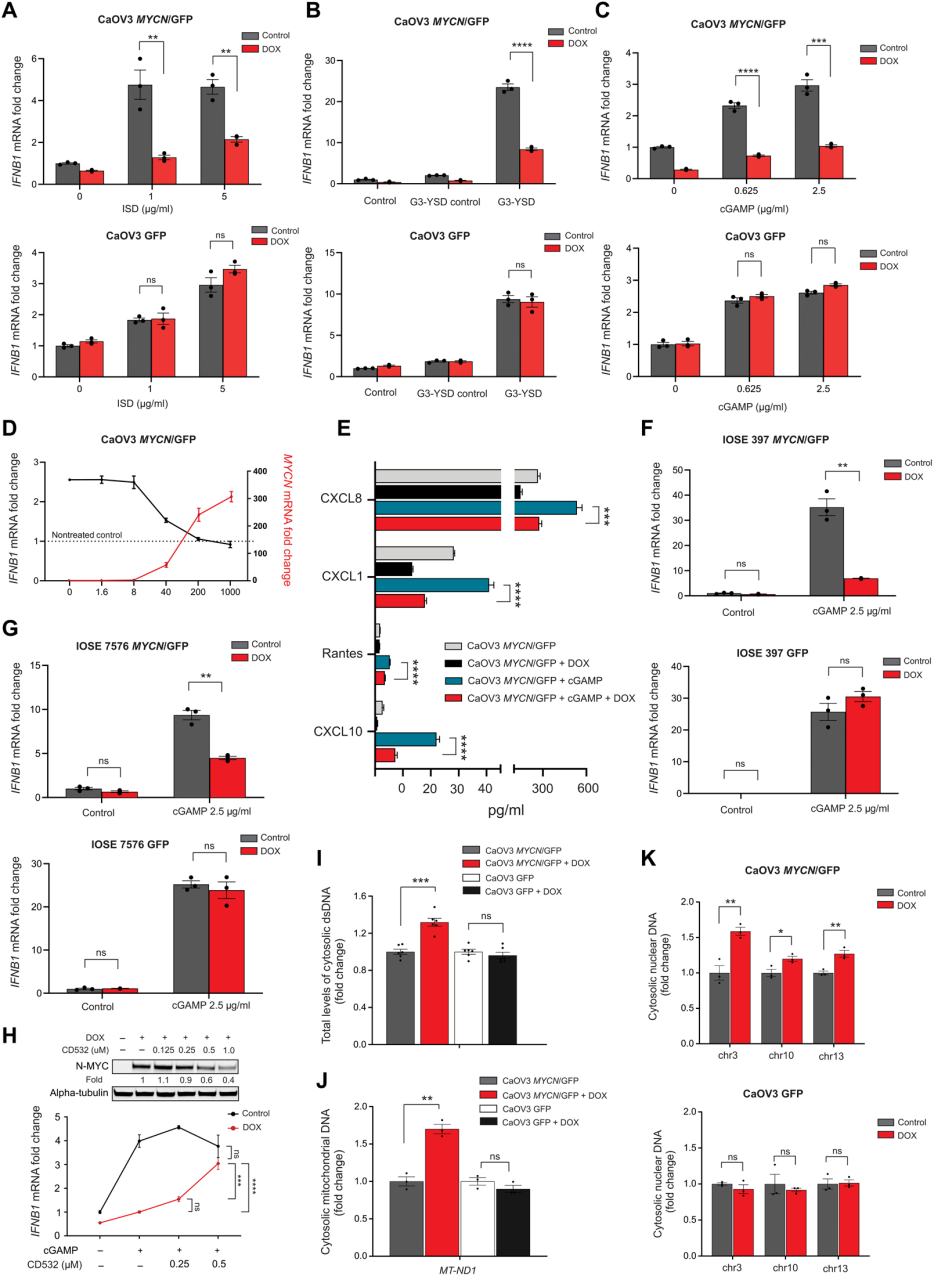
N-MYC increases cytosolic dsDNA while repressing the cGAS/STING signaling pathway. (**A** to **C**) qRT-PCR of *IFNB1* in CaOV3 *MYCN*/GFP and GFP cells pretreated ± DOX (1 μg/ml) for 72 hours and transfected for 24 hours with the indicated concentrations of ISD (A), G3-YSD versus G3-YSD control (B), or 2′3′-cGAMP (C). (**D**) qRT-PCR of *IFNB1* expression in CaOV3 *MYCN*/GFP and GFP cells pretreated with the indicated concentrations of DOX, followed by transfection ± 2′3′-cGAMP (2.5 μg/ml). (**E**) CXCL8, CXCL1, RANTES, and CXCL10 protein levels in supernatants from CaOV3 *MYCN*/GFP cells treated ± DOX and transfected ± 2′3′-cGAMP (2.5 μg/ml). (**F**) qRT-PCR of *IFNB1* in IOSE-397 *MYCN*/GFP and GFP cells pretreated ± DOX (1 μg/ml) and transfected ± 2′3′-cGAMP (2.5 μg/ml). (**G**) Similar experiment as (F) but using IOSE-7576 cells. (**H**) Top: Immunoblot of N-MYC and α-tubulin in CaOV3 *MYCN*/GFP cells treated ± DOX and the indicated concentrations of the Aurora A/N-MYC inhibitor CD532. Bottom: qRT-PCR of *IFNB1* expression in CaOV3 *MYCN*/GFP cells cotreated ± DOX and the indicated concentrations of CD532, followed by transfection ± 2′3′-cGAMP (2.5 μg/ml). (**I**) QuBit quantification of dsDNA in cytoplasmic fractions from CaOV3 *MYCN*/GFP and GFP cells treated ± DOX. Means ± SEM of *n* = 6 biological replicates shown. (**J**) qRT-PCR of mitochondrial ND1 (*MT-ND1*) in cytoplasmic fractions from CaOV3 *MYCN*/GFP and GFP cells treated ± DOX. (**K**) qPCR of chromosomal DNA in cytoplasmic fractions from CaOV3 *MYCN*/GFP and GFP cells treated ± DOX. All *P* values were calculated using an unpaired two-tailed Student’s *t* test. Means ± SEM of *n* = 3 biological replicates shown. *P < 0.05; **P < 0.005; ***P < 0.001; ****P < 0.0001. Densitometry analysis was performed using ImageJ software.

This finding led us to evaluate whether N-MYC represses the cGAS/STING signaling pathway. Transfection of CaOV3 *MYCN* TET-On cells with dsDNA [specifically, non-CPG oligomer IFN stimulatory DNA (ISD)] resulted in dose-dependent induction of *IFNB1*, and this effect was suppressed by DOX-induced N-MYC expression (Fig. 5A). Similar results were seen using the cGAS-specific agonist G3-YSFD (Fig. 5B).

To further evaluate the impact of N-MYC on downstream STING activation, CaOV3 *MYCN* TET-On cells were transfected with 2′3′-cGAMP, a second messenger typically produced in mammalian cells by cGAS (cGAMP synthase) in response to cytoplasmic dsDNA.2′3′-cGAMP activates innate signaling by binding to STING and inducing the TBK1-IRF3–dependent production of IFN-β (*35*). DOX-induced N-MYC expression abrogated 2′3′-cGAMP–induced *IFNB1* mRNA expression in a dose-dependent manner (Fig. 5, C and D), as well as the expression of *IFNL1* and *IFNL2* mRNA, indicating that N-MYC suppresses dsDNA sensing downstream of cGAS (fig. S10, C and D). Moreover, multiplexed cytokine and chemokine profiling revealed decreased secretion of CXCL10, RANTES, CXCL8, and CXCL1 following 2′3′-cGAMP stimulation in the presence of DOX (Fig. 5E). Similar results were seen in the immortalized ovarian surface epithelial cells IOSE-397 and IOSE-7576 (Fig. 5, F and G).

To confirm that the effects we were observing on the cGAS/STING pathway were due to N-MYC activity, we treated CaOV3, JHOS2, and NIH:OVCAR3 *MYCN* TET-On cells with CD532, an Aurora A kinase inhibitor that promotes N-MYC degradation. In all three HGSC cell lines, CD532 treatment reduced DOX-induced N-MYC protein levels and restored 2′3′-cGAMP–induced *IFNB1* mRNA expression (Fig. 5H and fig. S15, A and B). To assess this mechanism in the context of natural *MYCN* amplification and the chronically high levels of N-MYC expression that ensue, we performed loss-of-function experiments using the neuroblastoma cell line Kelly (*36*). Consistent with the results obtained with DOX-induced N-MYC, enhanced basal and 2′3′-cGAMP–induced expression of ISGs was seen after *MYCN* knockdown or pharmacological inhibition in Kelly cells (fig. S16, A to D).

**Fig. 6.**
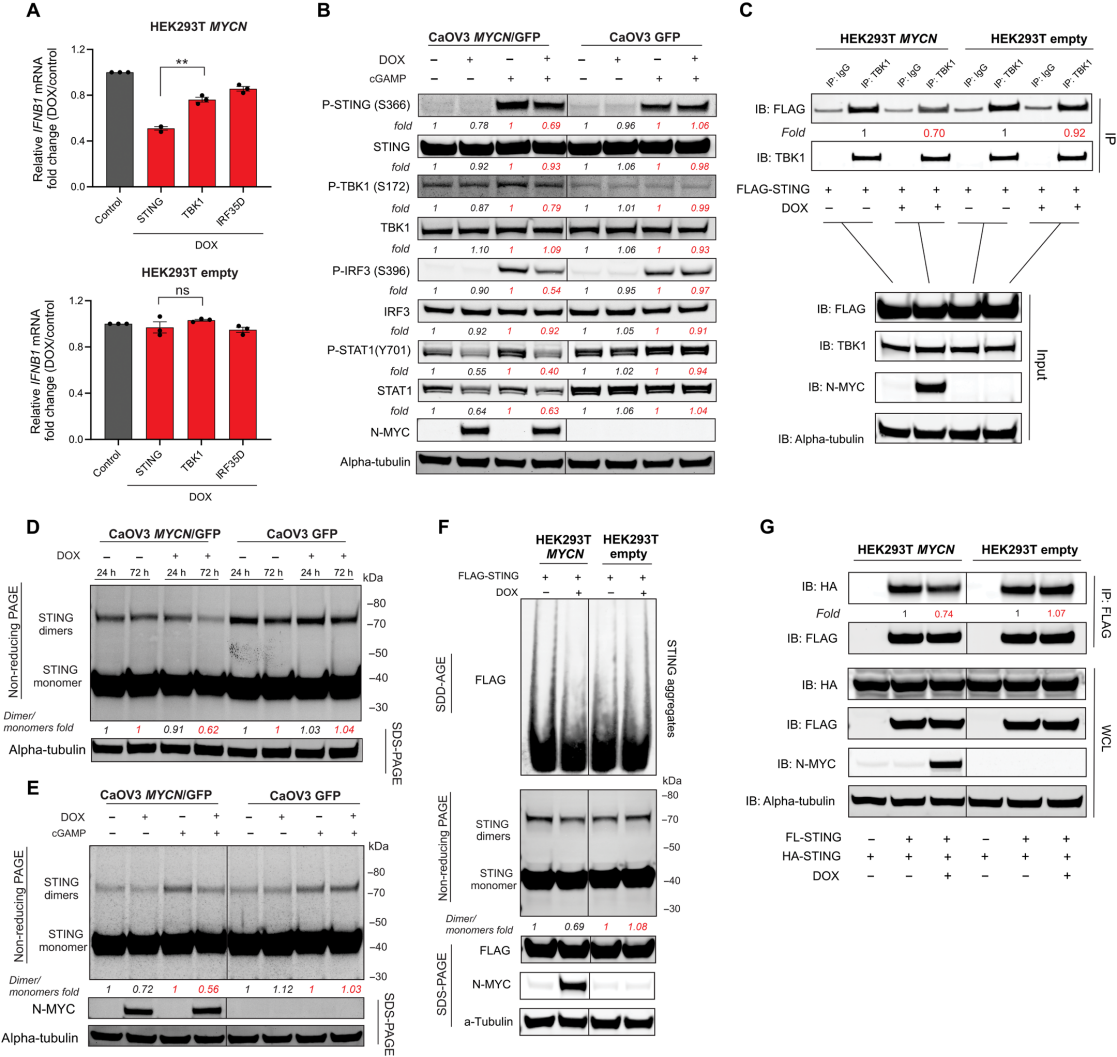
N-MYC affects STING phosphorylation and oligomerization. (**A**) qRT-PCR of *IFNB1* expression in HEK293T *MYCN* and empty cells pretreated ± DOX and transfected with the indicated plasmids. Mean ± SEM of *n* = 3 biological replicates are shown. (**B**) Immunoblot with the indicated antibodies against lysates from CaOV3 *MYCN*/GFP and GFP cells pretreated ± DOX and transfected ± 2′3′-cGAMP (5 μg/ml). Data are representative of three independent experiments. (**C**) HEK293T *MYCN* and HEK293T empty vector cells pretreated ± DOX and transfected with pFLAG-STING for 24 hours, lysed, immunoprecipitated and immunoblotted with the indicated antibodies. Data are representative of two independent experiments. (**D**) CaOV3 *MYCN*/GFP and GFP cells were pretreated ± DOX for 24 or 72 hours. Cell lysates were run by SDS-PAGE and analyzed by immunoblots with the indicated antibodies. Data are representative of two independent experiments (**E**) CaOV3 *MYCN*/GFP and GFP cells were pretreated ± DOX and transfected ± 2′3’-cGAMP (5 μg/ml). Cell lysates were run by SDS-PAGE and analyzed by immunoblots with the indicated antibodies. Data are representative of three independent experiments. (**F**) HEK293T *MYCN* and HEK293T empty vector cells were pretreated ± DOX, transfected with pFLAG-STING for 24 hours, and transfected ± 2′3′-cGAMP (5 μg/ml) for another 4 hours. Cell lysates were run by SDD-AGE or SDS-PAGE and analyzed by immunoblots with the indicated antibodies. Data are representative of four independent experiments. (**G**) HEK293T *MYCN* and empty vector cells pretreated ± DOX were cotransfected with pFLAG-STING and pHA-STING, lysed, immunoprecipitated with anti-FLAG–conjugated magnetic beads, and immunoblotted with the indicated antibodies. Data are representative of two independent experiments. All *P* values were calculated using an unpaired two-tailed Student’s *t* test. *P < 0.05; **P < 0.005; ***P < 0.001; ****P < 0.0001. Paired comparisons are shown in the same color for densitometry fold changes. Densitometry analysis was performed using ImageJ software.

We next investigated the effects of N-MYC on the cGAS/STING pathway in the absence of exogenous dsDNA. N-MYC can induce genomic instability and DNA damage in multiple ways (*37*), which in turn can result in accumulation of cytoplasmic dsDNA. DOX-induced expression of N-MYC in CaOV3 *MYCN* TET-On cells led to increased cytosolic dsDNA (Fig. 5I), which by qPCR was found to be preferentially derived from mitochondria as opposed to nuclear DNA (Fig. 5, J and K). Despite this increase in cytoplasmic dsDNA, downstream activation of STING remained suppressed (Fig. 6D). Thus, N-MYC increases cytosolic mitochondrial DNA (mtDNA) while at the same time inhibiting the cGAS/STING signaling pathway.

### The effects of N-MYC on nucleic acid sensing are independent of a direct transcriptional mechanism

To assess whether N-MYC regulates the cGAS/STING pathway by a transcriptional mechanism, we performed ChIP followed by high-throughput sequencing (ChIP-seq) to identify N-MYC–binding site across the genome after DOX treatment of CaOV3 *MYCN* TET-On cells (fig. S17, A to D). Consistent with prior reports (*38*), N-MYC–bound genes were associated with translation initiation and regulation, ribosome biogenesis, and rRNA metabolism. Unexpectedly, we did not detect significant binding of N-MYC to genes involved in IFN type I signaling or nucleic acid sensing (fig. S18, A and B), indicating that regulation of these pathways by N-MYC does not involve direct transcriptional repression. Accordingly, except for RIG-I and MDA-5 (classical ISGs), we did not observe any major changes at the mRNA and protein levels of multiple components of cytosolic nucleic acid sensing pathways after DOX treatment of CaOV3 *MYCN* TET-On cells (fig. S18, C and D).

To validate this conclusion by an independent means, we used the N-MYC mutant V421D, which has greatly reduced affinity for the corepressor MIZ1 (*39*). We generated CaOV3 *MYCN* V41D TET-On cells (fig. S18E) and stimulated them with 2′3′-cGAMP in the presence or absence of DOX. Similar to wild-type *MYCN* TET-On cells, DOX treatment of CaOV3 *MYCN* V41D TET-On cells inhibited the basal expression of ISGs (fig. S18F) and 2′3′-cGAMP–induced *IFNB1* mRNA expression (fig. S18G). Collectively, our findings indicate that repression of nucleic acid sensing by N-MYC does not involve direct transcriptional repression of genes in the relevant pathways.

### N-MYC suppresses STING phosphorylation and oligomerization

To investigate alternative mechanisms by which N-MYC could repress cGAS/STING signaling, we turned to the HEK293T *MYCN* TET-On cell line (fig. S12, A to C), which facilitated the experimental manipulation of various signaling intermediates. Transfection of FLAG-tagged STING, TBK1, or IRF35D into HEK293T *MYCN* TET-On cells induced *IFNB1* mRNA expression. DOX-induced N-MYC expression preferentially inhibited the ability of transfected STING to induce *IFNB1* mRNA, with a relatively weaker impact against transfected TBK1 or IRF35D (Fig. 6A). Following 2′3′-cGAMP transfection of CaOV3 *MYCN* TET-On cells, DOX-induced N-MYC expression led to reduced phosphorylation of STING, TBK1, IRF3, and STAT1 (Fig. 6B). Moreover, we observed reduced recruitment of endogenous TBK1 to transfected FLAG-STING in DOX-treated HEK293T *MYCN* TET-On cells (Fig. 6C). These data indicate that N-MYC represses the cGAS/STING signaling pathway primarily by acting on STING.

STING is a transmembrane protein, which, in the absence of stimulation, is anchored on the endoplasmic reticulum (ER). Upon binding of cGAMP, STING molecules undergo structural rearrangements that lead to lateral oligomerization along the ER surface (*40*). STING oligomers then translocate to the Golgi apparatus, where TBK1 is recruited (*41*). To detect oligomerization of STING, we performed nonreducing SDS–polyacrylamide gel electrophoresis (SDS-PAGE), which can depolymerize the STING polymer and preserve the dimer (*42*). DOX-induced N-MYC expression in CaOV3 *MYCN* TET-On cells led to a decrease in baseline STING oligomer levels after 72 hours (Fig. 6D). Treatment with 2′3′-cGAMP led to increased STING oligomerization, an effect that was suppressed by DOX treatment (Fig. 6E). In parallel experiments in HEK293T *MYCN* TET-On cells transfected with FLAG-tagged STING, DOX treatment also reduced STING oligomers (Fig. 6F, bottom). Evaluation of HMW STING oligomers by SDD-AGE validated the N-MYC–-mediated suppression of 2′3′-cGAMP and ligand-independent induced STING oligomerization (Fig. 6F, top). Furthermore, coimmunoprecipitation experiments using two differentially tagged STING constructs also demonstrated reduced STING oligomerization following DOX-induced N-MYC expression (Fig. 6G). Thus, N-MYC impairs cGAS/STING signaling in part by attenuating STING oligomerization.

### N-MYC transcriptional program is associated with cancer cell–intrinsic repression of IFN type I signature genes in human HGSC clinical samples

**Fig. 7.**
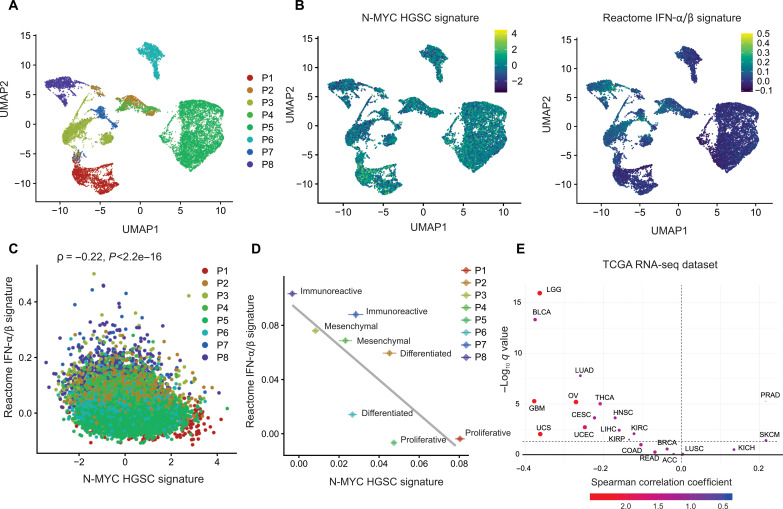
N-MYC is associated with cancer cell–intrinsic repression of IFN type I signature in human clinical samples. (**A**) Uniform Manifold Approximation and Projection (UMAP) of scRNA-seq from CD45^−^ cells (*n* = 13,966 cells, excluding fibroblasts) from eight HGSC cases (P1 to P8), showing that tumor cell phenotypes cluster by patient. (**B**) UMAP plot with each cell color-coded for the N-MYC HGSC gene signature (left) and the Reactome IFN-α/β pathway gene signature (right) computed on that cell (color scale is defined in the inset). (**C**) N-MYC HGSC and the Reactome IFN-α/β pathway gene signatures negatively correlate across all patients’ tumor cells. (**D**) Mean N-MYC HGSC gene signature and mean IFN-α/β signature negatively correlate across patients; means ± SEM across cells shown for each patient (ρ = −0.76; *n* = 8). Molecular subtype classification is indicated for each patient based on Nanostring data from FFPE whole-tumor sections (*43*). (**E**) Volcano plot reveals that *MYCN* mRNA expression and type I IFN signaling (Reactome IFN-α/β pathway ssGSEA) are negatively correlated in high *MYCN*–expressing cancers (top left quadrant). The *x* axis represents Spearman correlation ρ values, and the *y* axis represents −1 × log_10_ FDR *q* value for each gene. The dashed line indicates FDR *q* value = 0.05. Each point represents an individual cell except in (D), where each point represents one patient, and (E), where each point represents one cancer type.

We next evaluated the association between N-MYC transcriptional program and IFN type I genes in primary, untreated tumor samples from eight patients with HGSC. The CD45^−^ cell compartment was subjected to scRNA-seq, which yielded a total of 13,966 tumor cells for analysis (Fig. 7A). We generated an N-MYC HGSC signature (composed of genes identified as up-regulated in DOX-treated CaOV3 *MYCN* TET-On cells, above) and validated its positive correlation with *MYCN* expression based on Nanostring data from formalin fixation and paraffin embedding(FFPE) whole-tumor sections of the same samples (fig. S19A) (*43*); use of Nanostring data was required for this comparison, as *MYCN* mRNA was not reliably detected in scRNA-seq data (presumably due to “gene drop out”). In a within-patient analysis, we found a modest negative association between the N-MYC HGSC signature and IFN type I gene signature, and this reached significance in seven of eight patients (Fig. 7B and fig. S19B). Pooling data from across patients, the N-MYC HGSC signature showed a clear negative association with the type I IFN signature across all cells (*r* = −0.22; *P* < 2.2 × 10^−16^), irrespective of patient (Fig. 7C). This negative association was primarily driven by a notable inter- rather than intra-patient negative association in these signatures. Accordingly, mean patient N-MYC and IFN type I signatures displayed a strong negative correlation despite the modest sample size, mainly driven by C2/immunoreactive and C5/proliferative cases (Fig. 7D). Thus, in clinical HGSC samples, inferred N-MYC activity is associated with cancer cell–intrinsic repression of the type I IFN gene expression program, with individual patients exhibiting distinct “set points” for N-MYC activity.

We also analyzed cancer cell–intrinsic STING (*TMEM173*) expression levels in the scRNA-seq dataset (fig. S20A). This revealed a negative but nonsignificant correlation between mean patient STING expression and the *MYCN* signature (fig. S20B). This is consistent with our data suggesting that MYCN regulates STING posttranslationally rather than transcriptionally.

Last, we evaluated whether the negative association between *MYCN* and IFN type I signatures extended to other cancer types. Using tumor purity–corrected RNA-seq data from TCGA (Pancan21 dataset, *n* = 8290 tumors), we found a strong negative relationship between median *MYCN* expression and median IFN type I signature within cancer types with high *MYCN* expression (Fig. 7E). To address this at the protein level, we used proteomic data for 54 cancer cell lines from the CCLE, which revealed a negative relationship between N-MYC and IFN type I signature–related proteins across multiple cancer types (fig. S19C).

## DISCUSSION

We describe a role for N-MYC in suppressing nucleic acid sensing and IFN type I signaling in HGSC. Our analysis revealed high expression of *MYCN* and downstream signature genes in HGSC compared to other cancer types. We also identified a strong association between N-MYC activity and numerous aspects of the immunologically cold tumor phenotype. Evaluation of the N-MYC–driven transcriptomic program in HGSC-derived cell lines revealed a robust suppression of type I IFN–regulated genes with concomitant repression of IFN ligands, basal and induced JAK/STAT signaling, and T cell chemokines and chemoattraction. Using multiple, independent HGSC cell lines, we showed that N-MYC–driven type I IFN repression was dose dependent and rapidly reversible. N-MYC suppressed tumor cell–intrinsic STING and RIG-I–like receptor signaling by inhibiting in vitro oligomerization of STING and MAVS, independent of transcriptional repression. Last, single-cell analysis of clinical HGSC samples validated the cancer cell–intrinsic connection between the N-MYC activity and suppression of IFN type I signaling.

Unexpectedly, the relatively high *MYCN* mRNA levels we found in HGSC samples contrasted with low/undetectable mRNA levels and no gene amplification in HGSC cell lines. In theory, this could reflect an inability of high *MYCN*–expressing HGSC tumor cells to give rise to stable cell lines. Alternatively, tumor microenvironmental features may be required to induce *MYCN* transcription in vivo. In neuroblastoma and neuroendocrine prostate cancer, activation of the anaplastic lymphoma kinase (ALK) receptor tyrosine kinase up-regulates *MYCN* transcription through a STAT3-dependent mechanism (*44*–*46*). Although this mechanism has yet to be investigated in HGSC, elevated expression of ALK protein was reported in 28% of HGSC samples (independent of *ALK* mutation or gene rearrangement), providing one potential mechanism for *MYCN* up-regulation in the tumor microenvironment (*47*, *48*). In general, the lack of *MYCN* amplification and/or expression in commonly used HGSC cell lines may explain why the role of N-MYC in regulating innate immune signaling pathways has not been fully recognized until now. It also presents challenges for modeling the effects of N-MYC on antitumor immunity in vivo. To address this, we are pursuing a patient-derived organotypic tumor spheroid approach, which allows modeling of the native tumor immune microenvironment in primary human tumor samples (*49*).

Basal ISG expression in cancer cells has been attributed to activation of the STING pathway in response to aberrant DNA species (*34*). Accordingly, we found that basal expression of ISG in HGSC-derived cell lines was dependent on the STING pathway. Moreover, by multiple lines of evidence, we showed that N-MYC can suppress cGAS/STING signaling in tumor cells. We detected N-MYC dose-dependent inhibition of several downstream events, including *IFNB1* induction, secretion of IFN-regulated chemokines, and cGAS/STING pathway activation after treatment with multiple agonists. Intriguingly, we observed these inhibitory effects across HGSC cell lines with diverse genomic backgrounds (CaOV3: *TP53* mutation, JHOS-2: *TP53* and *BRCA1* mutation, OVCAR3: *TP53* mutation and *C11orf30* and *CCNE1* amplification) (*50*, *51*), as well as in nonmalignant ovarian epithelial cell lines (IOSE-397 and IOSE-7576). This adds to a growing list of oncogenic/tumor suppressor genes that modulate the cGAS/STING pathway, including mutated p53, NF2, and LKB1 (*52*–*54*).

DNA sensing within tumor cells has been demonstrated to be essential for antitumor immunity, specifically in DNA repair–deficient and/or highly immunogenic tumor cells (*9*–*11*). Although suppression of the STING signaling pathway in ovarian cancer has been reported (*55*, *56*), the mechanistic basis of this phenomenon has been elusive until now. Using our DOX-inducible system, we found that N-MYC expression can inhibit the oligomerization of STING, which has previously been shown to be required for recruitment and transactivation of TBK1 (*40*). In turn, STING phosphorylation at S366 by TBK1 is critical for direct IRF3 recruitment and activation (*57*). We found that N-MYC inhibits the interaction between STING and TBK1, as well as STING phosphorylation at S366, possibly as secondary events to suppression of STING oligomerization. Our results further suggest that the regulation of innate immune signaling by N-MYC occurs through an indirect mechanism. In this regard, it was recently reported that N-MYC can induce profound lipid peroxidation, which in turn sensitizes cells to ferroptosis (*58*, *59*). Enhanced cellular lipid peroxidation has been linked to STING carbonylation at C88 and inhibition of STING trafficking from the ER to the Golgi complex (*60*), providing one potential mechanism through which N-MYC could repress the STING signaling pathway.

The cGAS-STING pathway can be activated by not only pathogen-derived DNAs but also self-DNAs, including mtDNA aberrantly localized in the cytosol under certain stress conditions (*61*, *62*). We observed an N-MYC–driven increase in cytosolic dsDNA (most notably mitochondrial dsDNA), which suggests that N-MYC, like c-MYC, may trigger the mitochondrial apoptosis pathway (*63*–*65*). Thus, our data suggest that N-MYC induces cytosolic mtDNA release while at the same time suppressing the cGAS/STING signaling pathway, therefore preventing cytosolic mtDNA from being detected and triggering innate immune signaling.

Accumulating evidence suggests that dsRNA can be produced in cancer cells from endogenous sources such as retroelements and mtDNA (*66*). c-MYC activation has been shown to promote biogenesis of RLR-stimulatory dsRNAs (*67*). Basal detection of RIG-I ligands within tumor cells is known to be required for antitumor immunity and response to anti-CTLA4 (*7*, *8*). Moreover, in a murine ovarian cancer model, increased exposure to RIG-I ligands by hypomethylating agents was shown to transform the tumor microenvironment and prolong animal survival (*68*). Notably, this dsRNA sensing pathway also appears to be inhibited by N-MYC. Specifically, we showed that N-MYC suppressed the RIG-I/MDA-5 pathway after Poly I:C treatment, as evidenced by inhibition of TBK1, IRF3, and STAT1 phosphorylation; *IFNB1* induction; and IFN-dependent chemokine secretion. This effect of N-MYC involved the inhibition of MAVS aggregation and localization in the mitochondria, without a major effect on K63-linked ubiquitination.

ChIP-seq revealed that N-MYC does not bind to the promoter region of genes associated with RIG-I/MDA-5 (including MAVS), cGAS/STING, or IFN type I signaling pathways. With the exception of STAT1, induced N-MYC expression did not cause any major changes in these innate immune genes at the mRNA or protein levels, indicating an indirect mechanism of regulation. These data are consistent with previous N-MYC ChIP-seq data in neuroblastoma, where no binding to innate immune signaling genes was reported (*38*). We hypothesized that N-MYC may indirectly regulate STAT1 transcription, for example, via altered DNA methylation or histone modifications. In contrast, c-MYC has been shown to repress type I IFN signaling by binding to the promoters of multiple IFN type I–regulated genes, including STAT1 and STAT2 (*69*–*72*). In addition, c-MYC has been shown to inhibit STING-dependent innate immunity by transcriptionally regulating STING (*73*). Thus, while both N-MYC and c-MYC repress multiple components of cancer-intrinsic innate immune signaling, they do so via different mechanisms. Adding further complexity, induced expression of N-MYC led to down-regulation of endogenous c-MYC, in agreement with a prior report of negative cross-regulation between these two MYC paralogs (*74*). This finding also rules out the possibility that N-MYC suppresses innate immune signaling by increasing expression of c-MYC.

STING and RIG-I agonists are being actively developed as therapeutic agents to overcome the immunologically cold tumor phenotype, including in HGSC (*75*, *76*). Our findings suggest that such approaches may be less effective in N-MYC–expressing tumors due to cell-intrinsic impairment of these pathways. As a means to potentially overcome this, several drugs targeting N-MYC are currently being tested in clinical trials (*77*). Our work suggests that such compounds could prove valuable for enhancing the efficacy of immunotherapies, including STING and RIG-I agonists, against HGSC and other cancer types with elevated N-MYC activity.

## METHODS

### Reagents and antibodies

DOX hyclate (#D5207) was purchased from Sigma-Aldrich. TBK1 inhibitor MRT67307 (#inh-mrt) was from InvivoGen. Recombinant human IFN-β protein (#499-IF) was from R&D Systems. Carboxyfluorescein diacetate succinimidyl ester (CFSE) cell proliferation kit (#C34554) was from Invitrogen. T cell TransAct (#130-111-160) was from Miltenyi Biotec. Recombinant human IL-2 (#HZ-1015) was from Proteintech. Zombie NIR viability dye (#423106) was from BioLegend. Aurora A/*MYCN* Dual Inhibitor, CD532 (#532605) was from Calbiochem. Mitomycin C (#10107409001) was from Roche. Poly(I:C) (HMW)/LyoVec (#tlrl-piclv), Poly(I:C) (LMW)/LyoVec (#tlrl-picwlv), G3-YSD (#tlrl-ydna), G3-YSD Control (#tlrl-ydnac), 2′3′-cGAMP (#tlrl-nacga23-02), ISD/LyoVec (#tlrl-isdc), and LyoVec (#lyec-12) were obtained from InvivoGen.

The following antibodies were used in this study: anti-Hu Fc receptor binding inhibitor (#50-112-9053, eBioscience), P-STAT1 (Tyr^701^) (#9167, Cell Signaling Technology), STAT1 (#14994, Cell Signaling Technology), P-STAT2 (Tyr^690^) (#4441, Cell Signaling Technology), STAT2 (#72604, Cell Signaling Technology), IRF9 (#76684, Cell Signaling Technology), α-tubulin (#3873, Cell Signaling Technology), DYKDDDDK Tag rabbit monoclonal antibody (mAb) (#14793, Cell Signaling Technology), DYKDDDDK Tag mouse mAb (#8146, Cell Signaling Technology), STING (#13647, Cell Signaling Technology), TATA box–binding protein (#8515, Cell Signaling Technology), CD3-PECy7 (#341111, BD Bioscience Technology), RIG-I (#3743, Cell Signaling Technology), MDA-5 (#5321, Cell Signaling Technology), P-TBK1 (Ser^172^) (#5483, Cell Signaling Technology), TBK1 (#3504, Cell Signaling Technology), P-IRF3 (Ser^396^) (#4947, Cell Signaling Technology), N-MYC (#51705, Cell Signaling Technology), VDAC (#4661, Cell Signaling Technology), MAVS (#24930, Cell Signaling Technology), c-MYC (#5605, Cell Signaling Technology), L-MYC (#76266, Cell Signaling Technology), P-STING (Ser^366^) (#50907, Cell Signaling Technology), hemagglutinin tag (#3724, Cell Signaling Technology), Myc tag (#2278, Cell Signaling Technology), cGAS (#15102, Cell Signaling Technology), anti-rabbit immunoglobulin G (IgG) (H+L) (DyLight 800 4× polyethylene glycol conjugate) (#5151, Cell Signaling Technology), and anti-mouse IgG (H+L) (DyLight 680 conjugate) (#5470, Cell Signaling Technology). Anti–IFN-α/β receptor 1 antibody (#ab10739) and goat IgG, polyclonal—isotype control (#ab37373) were purchased from Abcam.

### Cell culture and transfection

CaOV3 [American Type Culture Collection (ATCC): HTB-75] (*TP53* mutated) and HEK293T (ATCC: CRL-11268) cell lines were cultured in Dulbecco’s modified Eagle’s medium high glucose (DMEM-high glucose, HyClone) and supplemented with 10% fetal bovine serum (FBS; HyClone) or 10% tetracycline-free FBS (Wisent Bioproducts). Jurkat cells were cultured in RPMI 1640 media (Thermo Fisher Scientific) containing 10% FBS. JHOS-2 (*TP53* and *BRCA1* mutated) (gift from D. Bowtell) were cultured in DMEM/Ham F12 (1:1) (Gibco) supplemented with 0.1 mM nonessential amino acids and 10% FBS. NIH:OVCAR3 (ATCC: HTB-161) (*TP53* mutated and *C11orf30* and *CCNE1* amplification) cells were cultured in RPMI 1640 ATCC modification (Gibco) supplemented with 20% FBS. The IOSE-397 and IOSE-7576 cells (University of British Columbia) were cultured in a 1:1 mix of media 199 (#M5017, Sigma-Aldrich) and 105 (#M6395, Sigma-Aldrich) supplemented with 5% FBS. All cells were maintained in a humidified incubator at 37°C with 5% CO_2_.

Transfection of dsDNA and dsRNA was performed using LyoVec transfection reagent (InvivoGen) as per the manufacturer’s instructions and at the concentrations indicated in the figure legends. Transfections of plasmids and duplexes of siRNAs were performed using JetPrime reagent (VWR) as per the manufacturer’s instructions. siRNAs were purchased from Integrated DNA Technologies, and AllStars negative-control siRNA (Qiagen) was used as a control siRNA. All siRNA sequences are reported in table S2.

### Plasmids

cDNA open reading frame of human *MYCN* (NM_001293228.2) was synthetized and cloned into the pLVX-TRE3G-IRES (internal ribosomal entry site) (#631354, Takara Bio USA) using In-Fusion HD Cloning System (#638909, Takara Bio USA) followed by enhanced green fluorescent protein (eGFP) insertion into the multicloning site. To generate HEK293T *MYCN* TET-On inducible cell line, the blasticidin resistance cassette BSR was cloned into vector pLVX-EF1a-Tet3G. Expression vectors for transient transfections were generated by subcloning synthetized human *MAVS* (NM_020746.5), *TMEM173* (NM_198282.4), *IRF3 5D* (NM_001571; S396D/S398D/S402D/T404D/S405D), *TBK1* (NM_013254), and *RIG-IN* (N-terminal CARD region; 1 to 284 amino acids) into pcDNA3.1-C-(k)DYK (GenScript). All constructs were validated by sequencing. pCMV-Myc-*TBK1* and pCMV-HA-*STING* were a gift from L. Martinez (Stony Brook University). pRK5-HA-Ubiquitin-K63 was a gift from T. Dawson (Addgene plasmid #17606).

### Lentiviral production and generation of *MYCN* TET-On inducible cell lines

The TET-On 3G DOX-inducible expression system (#631354, Takara Bio USA) was used to induce the expression of *MYCN* under the control of the TRE3G promoter in CaOV3, JHOS-2, NIH:OVCAR3, IOSE-397, IOSE-7576, and HEK293T cells according to the manufacturer’s protocol. Briefly, HEK293T cells (ATCC, Manassas, VA) were plated in 10-cm plates at 4 × 10^6^ cells per plate. After 24 hours, the cells were transfected with 7 μg of target lentiviral construct, along with Lenti-X Packaging Single Shots and incubated overnight. The next day, fresh medium was added. At 48 hours after transfection, supernatant was harvested and passed through a 0.45-μm filter to remove cell debris as per the manufacturer’s instructions (Clontech). Virus-containing medium was stored at −80°C until use. A day before transduction, target cells were plated at density 4 × 10^5^ cells per well in culture media containing 10% tetracycline-free FBS in six-well plate. The next day, lentivirus supernatant from 293T cells expressing both regulator vector (pLVX-EF1a-Tet3G) and response vector (pLVX-TRE3G-eGFP-IRES-*MYCN* or pLVX-TRE3G-eGFP-IRES) were coinfected at a 1:1 multiplicity of infection ratio in the presence of polybrene (8 μg/ml). Target cells were selected with G418 or blasticidin and puromycin for another 15 days before further experiments. DOX (1 μg/ml)–induced mRNA and protein levels were confirmed by qPCR and Western blotting.

### Transcriptional profiling

CaOV3 *MYCN*/GFP and GFP control cells (2 × 10^5^) were seeded in triplicate in wells of a six-well plate. After 3 days of culturing in the presence or absence of DOX (1 μg/ml), total RNA was isolated using the RNeasy plus kit (Qiagen). Subsequently, cDNA was synthesized and labeled at Genome Quebec, and hybridization was conducted on Gene ChIP Clariom S Human Transcriptome Array (Thermo Fisher Scientific). Data were preprocessed using the R package OLIGO prior and limma to identify differentially expressed genes that were input to Ingenuity Pathway Analysis software (Ingenuity Systems, Qiagen) to characterize molecular networks and pathways.

### Nuclear and cytoplasmic fractionation

Cells were lysed in ice-cold cytoplasmic lysis buffer [10 mM Hepes (pH 7.4), 1 mM MgCl_2_, 0.05 mM EGTA, 0.5 mM EDTA, 1 mM dithiothreitol (DTT), and 0.2% NP-40] supplemented with a protease inhibitor cocktail. After centrifugation at 14,000*g* for 10 min, supernatants were collected as cytoplasmic extracts. Pellets were washed two times with cytoplasmic lysis buffer and then lysed in ice-cold nuclear lysis buffer [5 mM NaCl, 10 mM Hepes (pH 7.4), 1 mM MgCl_2_, 0.05 mM EGTA, 0.5 mM EDTA, 1 mM DTT, 0.2% NP-40, and 20% glycerol] supplemented with a protease inhibitor cocktail. After centrifugation at 14,000*g* for 20 min, the supernatants were collected as nuclear extracts and subjected to SDS-PAGE.

### Protein immunoprecipitation

Cells were collected 24 hours after transfection and lysed in lysis buffer [0.5% (v/v) NP-40, 10 mM tris-HCl (pH 7.5), 0.5 mM EDTA, and 150 mM NaCl] supplemented with a protease and phosphatase inhibitor cocktail (Cell Signaling Technology). After centrifugation for 10 min at 14,000*g*, supernatants were collected and incubated with anti-FLAG magnetic beads (Sigma-Aldrich) or Myc Trap magnetic beads (Chromotek) overnight at 4°C. Beads were washed three times with cold lysis buffer and eluted with 3×FLAG peptide (Sigma-Aldrich) or Laemmli sample buffer, respectively. For endogenous TBK1 immunoprecipitation assays, cell lysates were obtained in lysis buffer [20 mM tris-HCl (pH 7.5), 150 mM NaCl, 1 mM Na_2_EDTA, 1 mM EGTA, 1% Triton X-100, 2.5 mM sodium pyrophosphate, 1 mM β-glycerophosphate, 1 mM Na_3_VO_4_, leupeptin (1 μg/ml), and 1 mM phenylmethylsulfonyl fluoride (PMSF)]. Cell extracts were incubated overnight at 4°C, with 1 μg of anti-TBK1 antibody and protein A magnetic beads (Cell Signaling Technology). The next day, beads were washed with cold lysis buffer and resuspended in Laemmli sample buffer. All samples were heated at 95°C for 10 min.

### Immunoblotting

Cell lysates, quantified by Pierce bicinchoninic acid assay kit (Thermo Fisher Scientific) and resuspended in Laemmli sample buffer, were resolved by SDS-PAGE and transferred to polyvinylidene difluoride membranes. Membranes were blocked in Intercept (tris-buffered saline) blocking buffer (LICOR) for 1 hour at 37°C. Primary and secondary antibodies were diluted in blocking buffer/Tween (0.1%), and membranes were incubated overnight at 4°C or 1 hour at room temperature, respectively. Images were revealed and analyzed using Odyssey CLx (LICOR) and Image Studio Lite software.

### Semi-denaturing detergent agarose gel electrophoresis

As described previously (*32*), mitochondria were isolated using the qProteome mitochondria isolation kit (Qiagen), and mitochondria pellets were suspended in sample buffer [0.5× tris-borate EDTA (TBE), 10% glycerol, 2% SDS, and 0.0025% bromophenol blue] and subjected to SDD-AGE. Samples were loaded onto a vertical 1.5% agarose gel (Bio-Rad). After electrophoresis in the running buffer (1× TBE and 0.1% SDS) for 40 min with a constant voltage of 80 to 100 V at 4°C, the proteins were transferred to Immobilon membrane (Millipore) for immunoblotting.

### RNA isolation, cDNA generation, and real-time qPCR

Total RNA was isolated using the RNeasy plus kit (Qiagen). To eliminate genomic DNA, RNA samples were additionally treated with deoxyribonuclease I (DNase I; Thermo Fisher Scientific). One microgram of total RNA was reverse-transcribed using High-Capacity cDNA Reverse Transcription kit (Thermo Fisher Scientific). qPCR was performed using Luna Universal qPCR Master Mix (NEB) on a StepOne Plus qPCR system (Applied Biosystems, Foster City, CA). *GAPDH* (Integrated DNA Technologies) mRNA level was used for normalization, and the relative expression levels of genes were calculated with the 2−ΔΔCt method. All primer sequences are reported in table S1.

### Isolation of cytoplasmic dsDNA

Cytoplasmic DNA was extracted by using the qProteome mtDNA isolation kit (Qiagen) according to the modified manufacturer’s instructions. Total levels of cytoplasmic dsDNA in the cytosol were quantified using the Qubit dsDNA HS assay kit (Thermo Fisher Scientific) and the Qubit Fluorometer (Thermo Fisher Scientific). Cytoplasmic DNA was isolated from cytosolic fractions using the QIAquick nucleotide removal kit (Qiagen) and eluting in 50 μl of elution buffer. The amount of mtDNA in cytosol was determined by qPCR using MT-ND1 primers. The amount of nuclear DNA in cytosol was determined by qPCR using three different sets of primers designed for different chromosomes as described previously (*78*). The sequences of the primers are listed in table S2.

### Microfluidic culture

CaOV3 *MYCN*/GFP and GFP TET-On control cells were pretreated with DOX (1 μg/ml) for 72 hours, after which 2.5 × 10^4^ cells were resuspended in type I rat tail collagen (Corning) at a concentration of 2.5 mg/ml following addition of 10 × phosphate-buffered saline with phenol red with pH adjusted using NaOH. The cell-collagen mixture was then injected into the center gel region of the 3D microfluidic culture device. Microfluidic culture devices were designed with a central region containing the cell-collagen mixture, surrounded by two media channels located on either side formed by bonding a coverslip to a patterned polydimethylsiloxane substrate. Collagen hydrogels containing cells were incubated for 30 min at 37°C and then hydrated with media with or without 2.5 × 10^4^ CFSE-labeled Jurkat T cells in the side media channels in the presence or absence of DOX (1 μg/ml). Jurkat T cells were labeled with the CFSE Cell Division Tracker Kit (BioLegend) following the manufacturer’s instructions. After 48 hours of incubation, images were captured on a Nikon Eclipse 80i fluorescence microscope equipped with Z-stack (Prior) and CoolSNAP charge-coupled device camera (Roper Scientific). Image capture and analysis were performed using NIS-Elements AR software package. Whole device images were achieved by stitching in multiple captures. Cell quantitation was performed by measuring the total cell area of CFSE dye.

### Cytokine profiling

Multiplex assays were performed using the Human Cytokine/Chemokine 48-Plex Discovery Assay Array (HD48) on a Luminex bead-based assay (Eve Technologies). Conditioned media concentration levels (picograms per milliliter) of each protein were derived from five-parameter curve-fitting models. Fold changes relative to the corresponding control were calculated and plotted as log_2_(fold change) (log_2_FC). Lower and upper limits of quantitation were imputed from standard curves for cytokines above or below detection.

### Chromatin immunoprecipitation sequencing

CaOV3 *MYCN*/GFP TET-On cells were cultured with ± DOX (1 μg/ml) for 72 hours. ChIP-seq was performed using the ChIP-IT High-Sensitivity kit (Active Motif) according to the manufacturer’s instructions. Briefly, 5 × 10^6^ to 10 × 10^6^ cells were harvested and fixed for 15 min in a 1% formaldehyde solution. Cells were lysed and homogenized using a Dounce homogenizer, and the lysate was sonicated (25% amplitude, duty cycle 30, 30-s ON and 30-s OFF for a total elapsed time of 30 min per sample in a Branson 450 Sonicator). Between 10 and 30 μg of the resulting sheared chromatin was used for each immunoprecipitation.

For N-MYC, 5 μg of N-MYC antibody (#61185, Active Motif) or normal rabbit IgG (#2729, Cell Signaling Technology) was used per reaction. For IRF9, 5 μg of IRF-9 antibody (#76684, Cell Signaling Technology) or normal rabbit IgG (#2729, Cell Signaling Technology) was used per reaction. Chromatin was incubated with primary antibodies overnight at 4°C on a rotator followed by incubation with Protein G agarose beads for 3 hours at 4°C on a rotator. Reversal of cross-links and DNA purification were performed according to the ChIP-IT High-Sensitivity instructions.

For ChIP-qPCR experiments, immunoprecipitated DNA was analyzed by quantitative reverse transcription PCR (qRT-PCR), and the amplification product was expressed as percentage of the input. ChIP-PCR primer pairs of indicated genes are listed in table S1.

For ChIP-seq experiments, sequencing libraries were constructed by Genome Quebec using Shotgun library preparation for ChIP samples (NEB Ultra II) and library sequencing was performed on a NovaSeq 6000 system (Illumina) with paired-end 2 × 50 base pairs. Two replicates from independent biological replicates were generated for the N-MYC ChIP-seq experiment.

Read alignment was performed against the GRCh38 genome build using BWA mem2 with default parameters. MACS2 was used to perform peak calling, and the R packages diffbind and ChIPseeker were used to call differentially enriched peaks and annotate the genomic regions (fig. S17, A and B). Peak regions were examined for the presence of consensus *MYCN* binding motifs using the STREME web server (fig. S17D) (*79*).

Functional annotation maps of N-MYC–bound genes were generated by testing for enriched GO “Biological Process” terms using the ClueGo plugin (*80*) within the Cytoscape framework (*80*), adjusting for multiple comparisons using the Bonferroni correction. GO terms with an adjusted *P* value of <0.01 were considered significant. A kappa score calculated reflecting the relationships between the terms based was set to 0.5 as the threshold in this study.

### Patient samples

Frozen primary tumor samples were collected through a prospective study entitled Immune Response to Ovarian Cancer (IROC) in partnership with the Tumor Tissue Repository (BC Cancer, Victoria, BC). All specimens were obtained with informed written consent under protocols approved by the Research Ethics Board of the BC Cancer Agency and the University of British Columbia. Samples were collected before patients received standard platinum-based chemotherapy. Briefly, freshly resected tumor samples were minced and enzymatically digested overnight at 4°C with a mix of collagenase, DNase I, and hyaluronidase (Sigma-Aldrich) to obtain a cell suspension that was filtered through a cell strainer (100 μm). Cells were then cryopreserved in liquid nitrogen until use.

### Generation of single-cell sequencing data

#### Sample preparation

After thawing, cryopreserved tumor samples were washed once with 10 ml of complete media [RPMI 1640 (#11875-093) with 10% FBS (#12483020), both purchased from Thermo Fisher Scientific] and filtered through a cell strainer (100 μm) to remove any large aggregates. Cell suspensions were then incubated in the dark with (i) Zombie NIR viability dye (BioLegend, San Diego, CA) for 15 min at room temperature, (ii) anti-Hu Fc receptor binding inhibitor (eBioscience, San Diego, CA) for 10 min at 4°C, (iii) fluorescein isothiocyanate–CD45 (BD Biosciences, clone: HI30) antibody for 30 min at 4°C, and (iv) 0.5 μg of a distinct TotalSeq-C antibody (hashtag multiplexing antibodies, BioLegend) per sample, according to the manufacturer’s protocol. Cell sorting was performed on a BD FACSMelody. Following doublet exclusion, Zombie NIRlow CD45^−^ cells were collected and placed on ice in low-binding 2-ml tube until all samples were ready for single-cell library preparation.

#### Single-cell library construction and sequencing

Cells were loaded into a Chromium Next GEM Chip K (PN-2000182) and processed according to the Chromium Next GEM Single Cell 5′ VDJ Reagent Kits v2 user guide (10X Genomics). Briefly, cells were lysed inside each gel bead-in-emulsion, for reverse transcription and cell barcoding, in the Chromium Controller (10X Genomics). Full-length cDNA along with cell barcode identifiers were PCR-amplified, and 5′ gene expression (5′GEX) libraries were constructed using Chromium Next GEM Single Cell 5′ Kit v2 (PN-1000263, 10X Genomics) and Dual Index Plate TT Set A (PN-1000215). 5′GEX libraries were sequenced on an Illumina NovaSeq.

#### Single-cell sequencing data analysis 

Reads were processed using CellRanger v5 with the GRCh38 human genome build. Additional analyses used Seurat, including for cell quality control (QC) and filtering (cells with >500 unique molecular identifiers (UMIs) and <25% mitochondrial reads retained), count normalization and scaling using SCTransform, and dimension reduction and projection using Uniform Manifold Approximation and Projection (UMAP). Feature barcodes were demultiplexed using MULTISeqDemux, with putative doublets or unassigned cells discarded. To remove nontumor cells (e.g., fibroblasts), cells were clustered using the Louvain method and clusters expressing fibroblast markers (e.g., *COL1A1*) were discarded. Cells from two patients with <200 cells recovered at this analysis stage were likewise excluded from further analysis. Signatures were scored for each cell using score.cells.puram in the Pagoda2 R package. Controlling for predicted cell cycle phase in linear models did not appreciably alter the results.

### Statistical analyses

Tests for differences between two group means were performed using two-tailed unpaired Student’s *t* test as specified in the figure legends. One-way analysis of variance (ANOVA) using the Tukey post hoc test or Kruskal-Wallis with Wilcoxon posttest for pairwise comparison was performed where applicable. *P* values were considered significant if less than 0.05. Asterisks used to indicate significance correspond with the following: *P < 0.05; **P < 0.005; ***P < 0.001, ****P < 0.001. R version 4.0 and GraphPad Prism9 were used for statistical analysis of experiments, data processing, and presentation.

## Supplementary Materials

### This PDF file includes:

Supplementary Methods Figs. S1 to S20 Tables S1 and S2 Legends for data S1 to S3 References
