# Supplementary material for: N-MYC impairs innate immune signaling in high-grade serous ovarian carcinoma
Source: Sci Adv. 2024 May 15;10(20):eadj5428. doi: 10.1126/sciadv.adj5428 (PMC11095474; doi:10.1126/sciadv.adj5428)
Supplement: Supplementary file 1 — Supplementary Methods Figs. S1 to S20 Tables S1 and S2 Legends for data S1 to S3 References [file sciadv.adj5428_sm.pdf]

Science Advances
AAAS

Supplementary Materials for

N-MYC impairs innate immune signaling in high-grade serous ovarian carcinoma

Alex Miranda *et al.*

Corresponding author: Brad H. Nelson, bnelson@bccrc.ca

Sci. Adv. **10**, eadj5428 (2024)
DOI: 10.1126/sciadv.adj5428

The PDF file includes:

Supplementary Methods
Figs. S1 to S20
Tables S1 and S2
Legends for data S1 to S3
References

Other Supplementary Material for this manuscript includes the following:

Data S1 to S3

Supplementary Methods

Cell proliferation, wound healing assay and soft agar colony formation assay. For cell proliferation assay, $5x10^3$ CaOV3, JHOS2 and OVCAR3 *MYCN*/GFP and GFP control cells were seeded in wells of a 96-well plate. After 1, 2, 3, 4 and 5 days of culture in the presence or absence of DOX (1 ug/ml) cells were resuspended and further incubated with LIVE/DEAD Fixable Violet stain. After 2 washes with PBS, cells were resuspended in PBS containing 2% FBS and count on a Cytek Aurora.

To perform wound healing assay, $5x10^5$ CaOV3, JHOS2 and OVCAR3 *MYCN*/GFP and GFP control cells were seeded in wells of a 24-well plate containing inserts of CytoSelect 24-Well Wound Healing Assay (Cell Biolabs, San Diego, CA) and treated ± DOX (1 ug/ml) until confluence was reached. The inserts were then carefully removed to produce 0.9-mm-diameter wounds, and fresh culture medium supplemented or not with DOX (1 ug/ml) was added to each well. The wound was monitored using a phase-contrast microscope at 0, 24, 48, and 72 hours (Leica Microsystems). The area of the non-healed wound was measured in 3 different regions.

Percent of wound closure were calculated as follow:

Percent of wound closure was calculated as (%) = Migrated cell surface area / Total surface area × 100

For anchorage-independent growth of CaOV3, JHOS2 and OVCAR3 *MYCN*/GFP and GFP control cells, 10^4 cells were transferred to 2 mL of culture medium containing 0.35% low melting point agarose (Sigma Aldrich). Cells were seeded in triplicate in 6-well plates containing a layer of solidified 0.6% agarose. Fresh medium was added every 3 days with ± DOX (1 ug/ml). Colonies were photographed at 40x magnification on day 12.

Evaluation of GFP$^+$ *MYCN* TET-ON cells by flow cytometry. *MYCN* TET-ON cancer cells were pre-treated with ± DOX (1 ug/ml) for 72 hours, resuspended, washed and further incubated with LIVE/DEAD Fixable Violet stain. After 2 washes with PBS, cells were resuspended in PBS containing 2% FBS and analyzed on a Cytek Aurora. The percentage of GFP positive cells were calculated by comparison with untreated live cells. The data analyses were performed with FlowJo software (TreeStar).

Inhibition of T cell proliferation assay. Human T cells were isolated using the Easy SEP Human T cell Isolation kit (Stem Cell Technologies) and labeled with 0.5 uM of CFSE (Life Technologies) following the manufacturer instructions. CaOV3 *MYCN*/GFP and GFP control cells were pre-treated with DOX (1 ug/ml) for 3 or 7 days and then treated with Mitomycin-C to a final concentration of 25 ug/mL for 15 min. CaOV3 cells were consequently washed 3 times and co-cultured in 24-well plates with CFSE-labeled T cells at different CaOV3: T cells ratios. T cells were activated with T Cell TransAct (Miltenyi Biotec) and 20 IU of IL-2 (Proteintech), harvested after 72 hours, and stained with PE-Cy7 anti-human CD3 (1:100 dilution, BD Bioscience) before acquisition on a Cytek Aurora.

TCGA data analysis. We accessed RNA sequencing as Transcripts Per Kilobase Million (TPM) mapped reads using the TCGAbiolinks R/Bioconductor package, for each cancer of interest. For genes with multiple annotated transcripts, we selected the transcript with the highest expression to represent the gene, then filtered the expression set to include only primary samples, removed patients with duplicate samples, and removed any patients without a consensus purity score in Aran et al. (28) to enable purity corrections in analyses. The data was log10 transformed for the purpose of visualization and downstream analysis (**Fig 1A**) to estimate association between median expression levels of *MYCN* gene expression and ssGSEA score of IFN signature (**Fig 7E**).

For the microarray HGSOC dataset (n = 486), we converted probe IDs to human gene symbols using biomaRt (81) and retained the probe with the highest expression for each gene. To identify transcriptomic patterns specific to ovarian cancer subtypes, purity corrected TCGA microarray data was utilized (**Fig 1B-D, F**). The subtype specific differentially expressed genes (DE) were utilized to reconstruct and analyze transcriptional regulatory networks using R Bioconductor package RTN. As a first step RTN combined subtype specific DE genes with transcription factors (TF) derived from FANTOM5 database to construct transcriptional networks using ARACNE (25) that utilized a mutual information-based network reconstruction algorithm. Next, RTN was used to perform an association test between each TF and its potential targets (DE genes) using the MRA algorithm (82). Further, a two tailed GSEA was performed to identify the top 20 TF regulated transcriptional networks or regulons using the association test output generated in the previous step. These top enriched regulons were clustered across the four different subtypes and were depicted in the heatmap based on the observed enrichment score, color coded as red and blue indicating positive and negative correlation to phenotype (**Fig 1C**). The phenotype and the rank ordered enrichment scores derived from GSEA depict the relationship between expression profile of *MYCN* and enrichment score of its target genes (**Fig 1D**).

Pathway analysis was conducted using the R package GAGE (83). The purity corrected TCGA microarray data was rank-ordered based on *MYCN* expression or *MYCN* HGSC signature and the low and high *MYCN* expressing or *MYCN* HGSC signature phenotypes comprised of the first and last 30 percentile samples of the rank-ordered were compared. A total of 6229 curated gene sets (C2) obtained from the human molecular signature database (MsigDB, version 7.2) were used. GAGE was performed with default parameters assuming that the gene sets are co-regulated with all the constituent genes either upregulated or downregulated. Top 30 statistically significant gene sets, sorted based on gene-set test statistic, are shown in (**Fig 1F and Fig S3B**).

The correlation between *MYCN* expression or *MYCN* HGSC signature and the cytolytic score (Geometric mean *GZMA*, *PRF1*) (27) was assessed across multiple ovarian cancer cell lines using data obtained from R Bioconductor package curatedOvarianData (26). We used nonparametric Spearman's correlation adjusting for multiple tests using the Benjamini–Hochberg method. The whiskers represent the bootstrapped (n =1000) confidence interval of the Spearman's correlation coefficient calculated using CRAN package RVAideMemoire (84). The pooled correlation coefficient was estimated using a random effect model using R package metafor (85) (**Fig 1E and Fig S3A**).

CCLE data analysis. The recent Cancer Cell Line Encyclopedia (Broad, 2019) data including copy number alteration, transcriptomic and protein expression was downloaded directly from the public cBioportal site. Gene expression profiles measured by RNA-seq, in Reads Per Kilobase of transcript per Million (RPKM) were used for estimating the median *MYCN* expression values and were log2 transformed for visualization across different tissues derived cell lines (**Fig S2A**). The segmentation mean profile of somatic copy number alteration data was used to visualize *MYCN* specific copy number changes in the corresponding tissues derived cell lines (**Fig S2B**). Reverse phase protein array derived protein expression data (86) represented as Z-scores values were used to estimate association between *MYCN* protein abundance and ssGSEA scores for the IFN signature (**Fig S19C**). R package GSVA (87) was used for the calculation of ssGSEA scores.

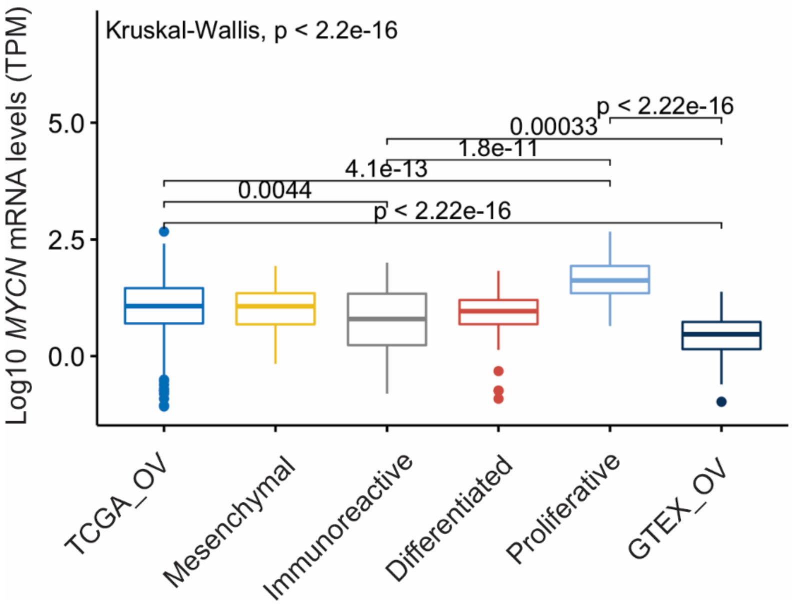

Fig. S1. *MYCN* mRNA levels across the four molecular subtypes of HGSC and on normal ovarian tissues.

Box plots depict expression of *MYCN* (TPM) in HGSC molecular subtypes and in ovarian normal tissues. Gene expression data was obtained from TCGA (The Cancer Genome Atlas) and GTEX (Genotype-Tissue Expression Project), respectively. The end of the whiskers represents Min to Max ranges. Kruskal Wallis with Dunn post-hoc test for pairwise comparison.

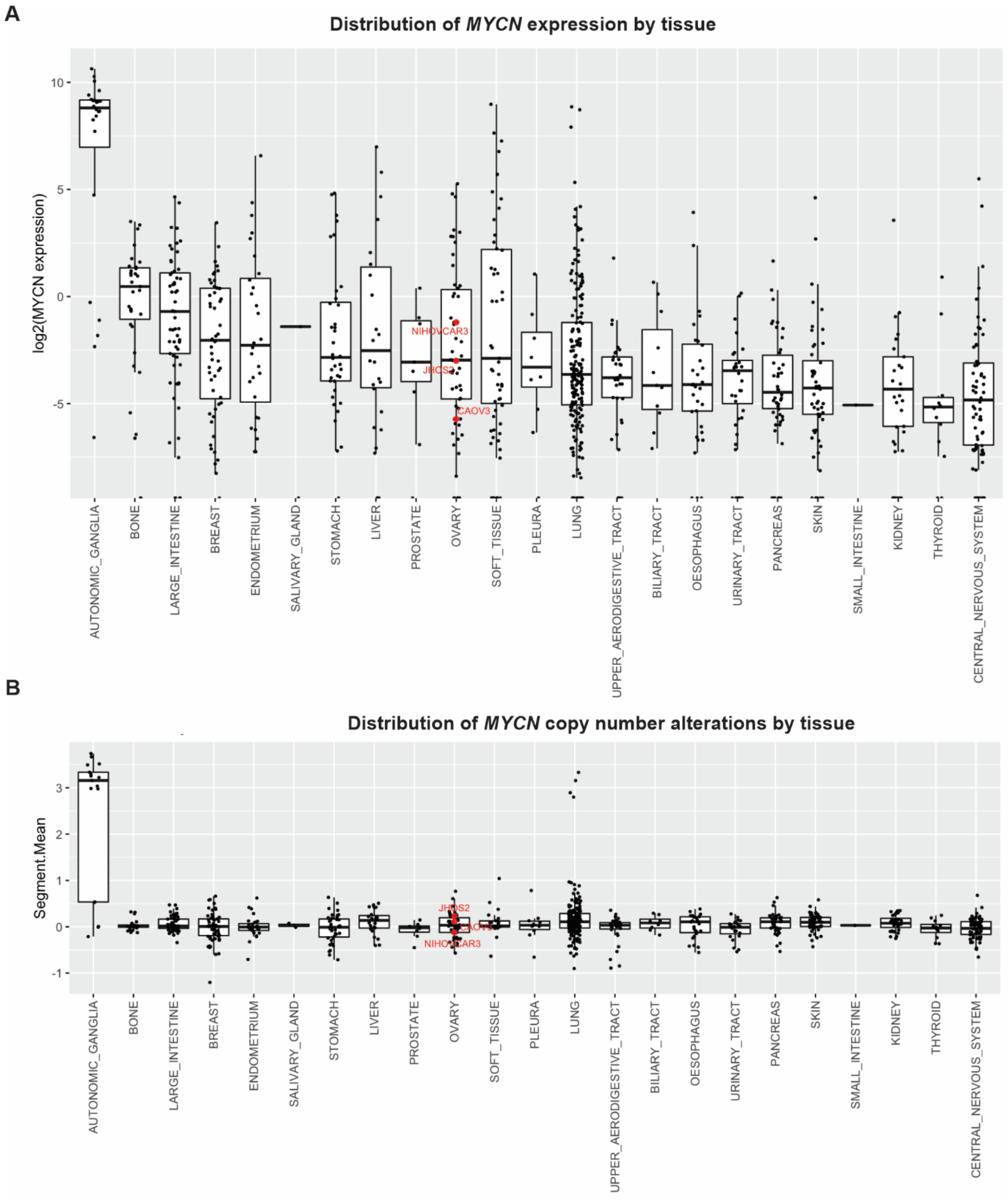

Fig. S2. *MYCN* mRNA levels and copy number alterations are comparatively lower in HGSC cell lines.

(A) *MYCN* RNA transcripts levels in Cancer Cell Line Encyclopedia (CCLE) models (n =921). **(B)** *MYCN* copy number alterations (CNA) in CCLE models (n =848). Each point represents an individual case, and cancer types are ordered by median *MYCN* CNA levels. Whiskers extend from

the lower to the upper adjacent value; the box extends from the 25th to the 75th percentile, with the median in each lineage as indicated by the line in the box.

A

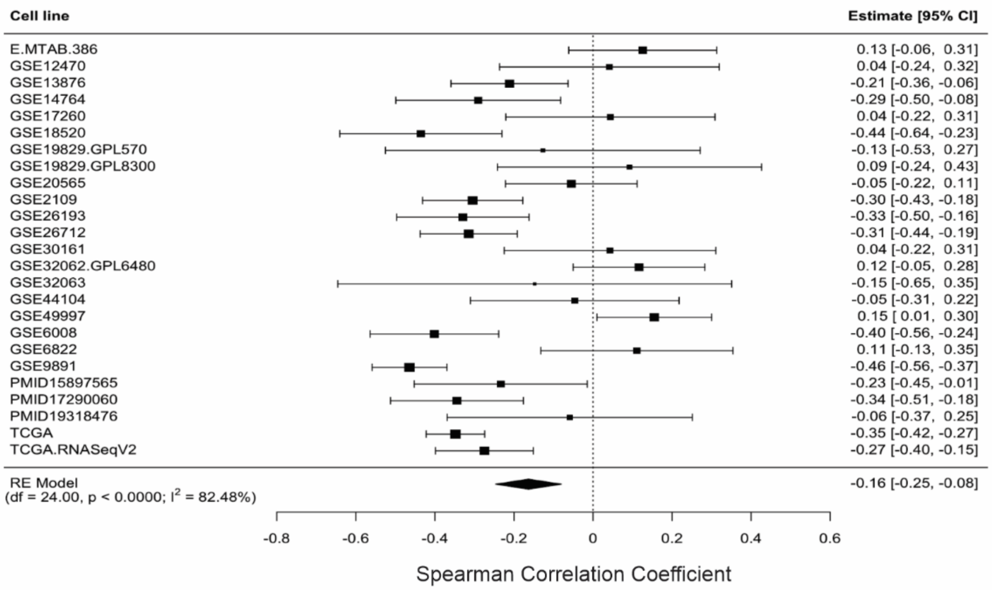

B

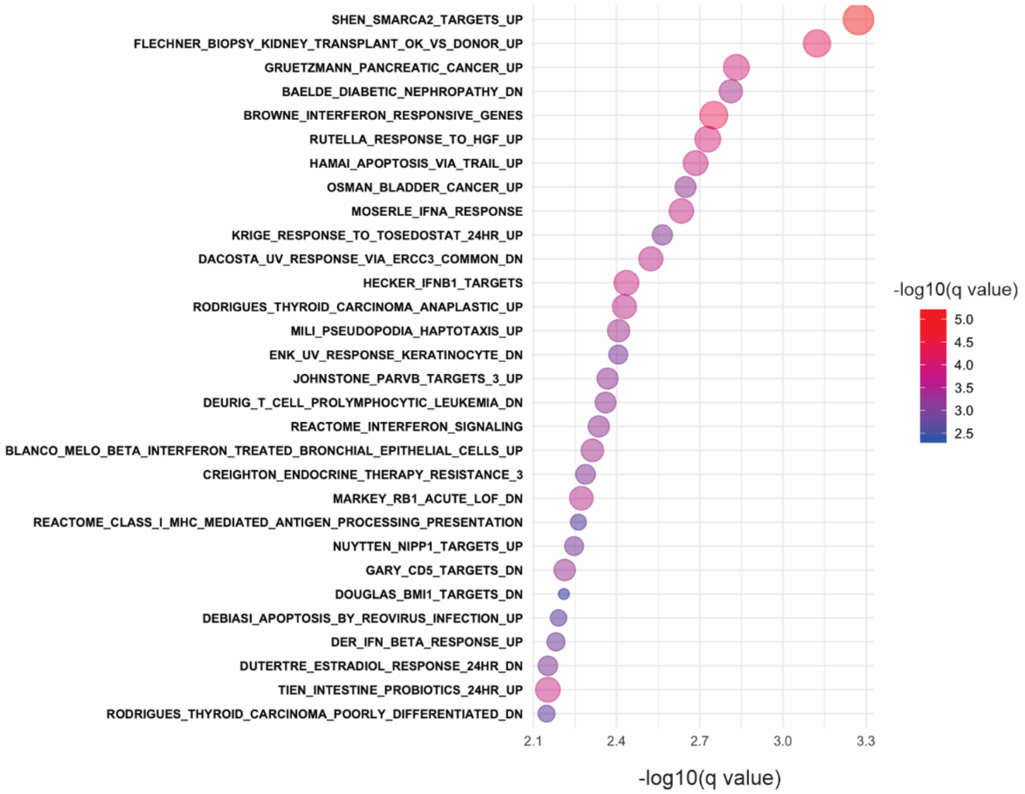

Fig. S3. ***MYCN*** **HGSC gene signature is negatively associated with features of anti-tumor immunity.**

(A) Forest plot depicting correlation of N-MYC HGSC gene signature with the immune cytolytic score (Geometric mean *GZMA*, *PRF1*(*27*) on multiple ovarian cohorts (*26*). Correlation analysis was performed using Spearman's rank method. **(B)** Gene signatures showing significant up-regulation in tumors scoring low for the N-MYC HGSC gene signature. GAGE (Generally Applicable Gene-set Enrichment) analysis was performed comparing high (higher than 70th

percentile) versus low (lower than 30th percentile) N-MYC HGSC gene signature tumors (TCGA). Pathway gene sets contained in the MSigDb (C2 genesets) database were used.

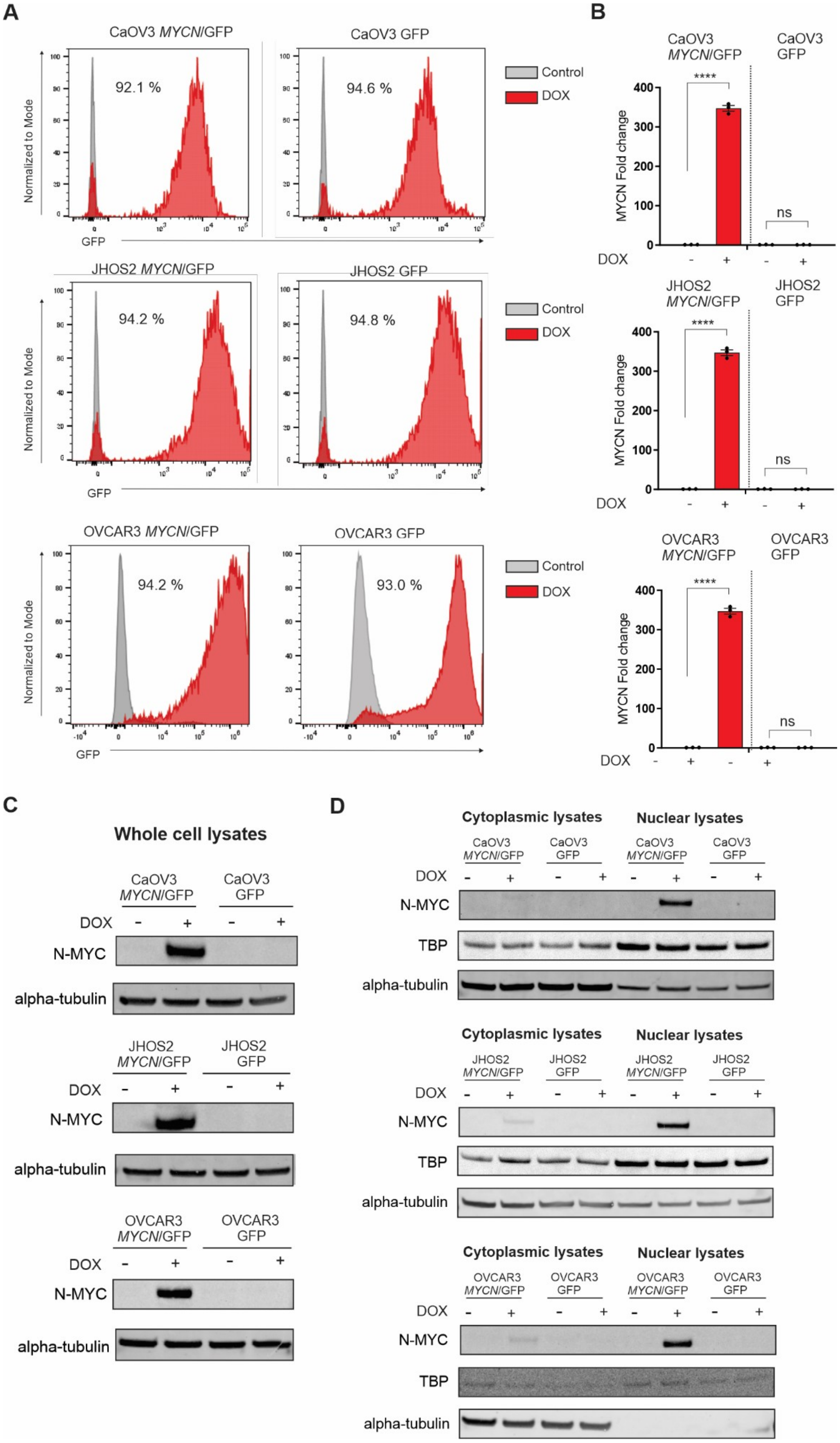

Fig. S4. ***MYCN*** **TET-On models in HGSC cell lines.**

(A) Flow cytometry quantification of GFP induction in CaOV3, JHOS2 and OVCAR3 *MYCN*/GFP and GFP controls cells pre-treated ± DOX for 72 hours. Representative fluorescence-activated cell sorting (FACS) histograms and statistical data are shown. **(B)** qRT-PCR of *MYCN* in CaOV3, JHOS2 and OVCAR3 *MYCN*/GFP and GFP controls cells pre-treated ± DOX for 72 hours. All P values were calculated using an unpaired two-tailed Student's t-test. Mean ± s.e.m of n=3 biological replicates shown. *P< 0.05; **P< 0.005; ***P< 0.001; ****P< 0.0001; ns, not significant. **(C-D)** Immunoblot of N-MYC, TBP and alpha-tubulin in CaOV3, JHOS2 and OVCAR3 *MYCN*/GFP and GFP controls cells pre-treated ± DOX for 72 hours. Whole cell lysates (WCL) **(C)** and nuclear and cytoplasmic lysates **(D)** were prepared and subject to western blot. Data are representative of 2 independent experiments.

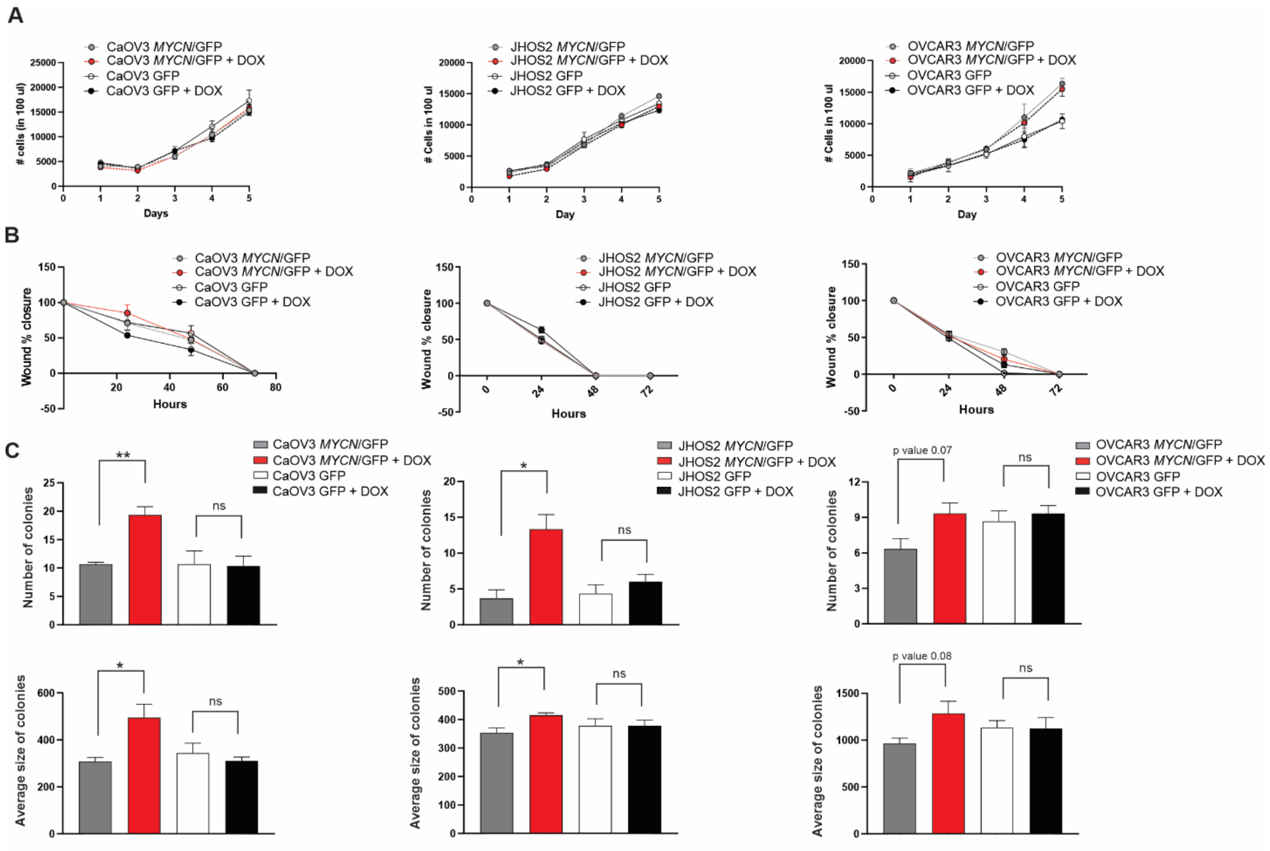

Fig. S5. N-MYC does not impact cell proliferation or migration but increases anchorage-independent growth.

(A) Growth curve showing the number of CaOV3, JHOS2 and OVCAR3 *MYCN*/GFP and GFP controls cells at 1, 2, 3, 4 and 5 days of cultured in the presence or absence of DOX. **(B)** Quantitation of wound closure rates at different time points of CaOV3, JHOS2 and OVCAR3 *MYCN*/GFP and GFP controls cells cultured in the presence or absence of DOX. **(C)** Number of colonies (*top*) and average size of colonies (*bottom*) in soft agarose of CaOV3, JHOS2 and OVCAR3 *MYCN*/GFP and GFP controls cells after 12 days of culture ± DOX. All P values were calculated using an unpaired two-tailed Student's t-test. Mean ± s.e.m of n=3 biological replicates shown. *P< 0.05; **P< 0.005; ***P< 0.001; ****P< 0.0001; ns, not significant.

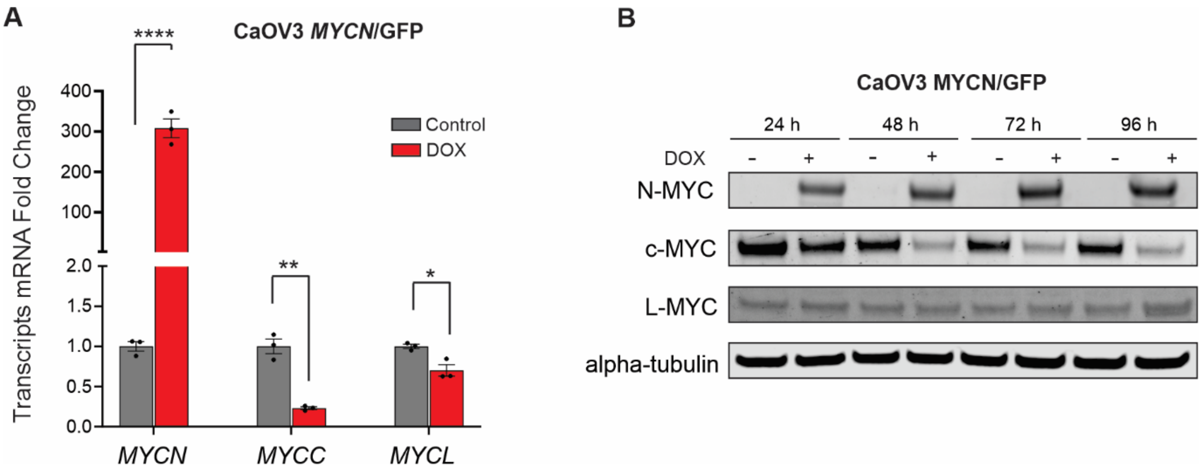

Fig. S6. N-MYC negatively regulates endogenous expression of c-MYC expression.

(A) qRT-PCR of *MYCN, MYCC and MYCL* in CaOV3 *MYCN*/GFP cells pre-treated ± DOX for 72 hours. All P values were calculated using an unpaired two-tailed Student's t-test. Mean ± s.e.m of n=3 biological replicates shown. *P< 0.05; **P< 0.005; ***P< 0.001; ****P< 0.0001; ns, not significant. **(B)** Immunoblot of N-MYC, c-MYC, L-MYC and alpha-tubulin in CaOV3 *MYCN*/GFP pre-treated ± DOX for different time points. Whole cell lysates were prepared and subject to western blot. Data are representative of 2 independent experiments.

A

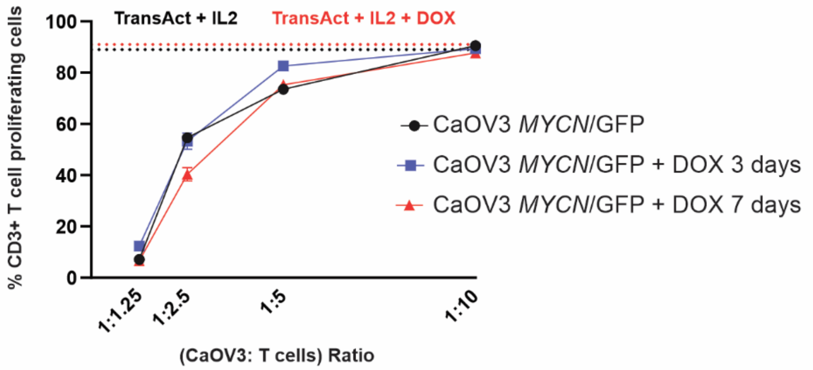

B

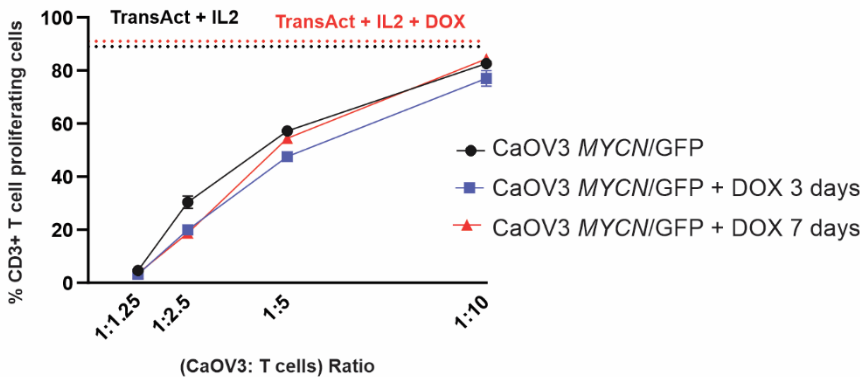

Fig. S7. N-MYC expression in CaOV3 *MYCN* TET-ON cells does not affect T cell proliferation.

T cell proliferation induced via CD3 and CD28 with T Cell TransAct™ and assayed as CFSE dilution by flow cytometry. CFSE-stained T cells were co-cultured with +/- DOX (1 ug/ml) pre-treated CaOV3 *MYCN*/GFP **(A)** and GFP controls cells **(B)** at different (CaOV3: T cells) ratios (1: 1.25), (1:2.5), (1:5) and (1:10). Data are expressed as percentage of CD3$^+$ T-cell proliferating cells (± s.e.m) of n=3 biological replicates. Dashed line represents the percentage of proliferating cells in the absence of CaOV3 TET-ON cells. Data are representative of 3 independent experiments.

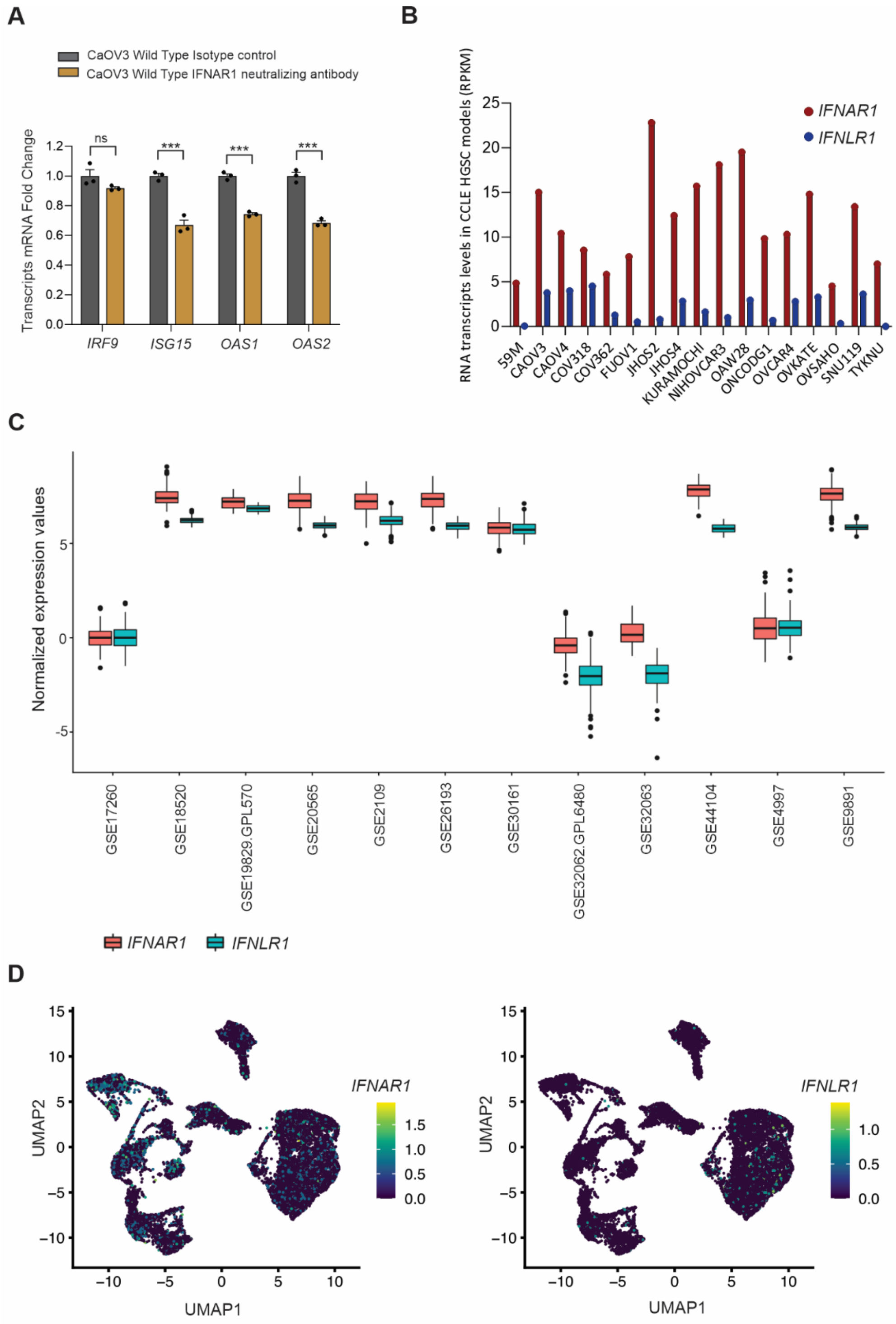

Fig. S8. ***IFNAR1*****, but not** ***IFNLR1,*** **is required for establishing the basal expression of ISG genes in HGSC cell lines**.

(A) qRT-PCR evaluation of ISGs (*IRF9, ISG15, OAS1* and *OAS2*) in CaOV3 wild type cells treated with 1ug/ml *IFNAR1* neutralizing antibody or 1 ug/ml of isotype control for 72 hours. P values were calculated using an unpaired two-tailed Student's t-test. Mean ± s.e.m of n=3 biological replicates shown. *P< 0.05; **P< 0.005; ***P< 0.001; ****P< 0.0001; ns, not significant. **(B)** *IFNAR1* and *IFNLR1* RNA transcript levels in HGSC cell lines from the CCLE (n =17). All P values were calculated using an unpaired two-tailed Student's t-test. Mean ± s.e.m of n=3 biological

replicates shown. *P< 0.05; **P< 0.005; ***P< 0.001; ****P< 0.0001; ns, not significant. **(C)** Box plots depicting expression of *IFNAR1* and *IFNLR1* on multiple ovarian cohorts (*26*). Whiskers extend from the lower to the upper adjacent value; the box extends from the 25th to the 75th percentile, with the median in each lineage as indicated by the line in the box. **(D)** UMAP plot with each cell color-coded for *IFNAR1 (left)* and *IFNLR1 (right)* computed on that cell (color scale is defined in the inset).

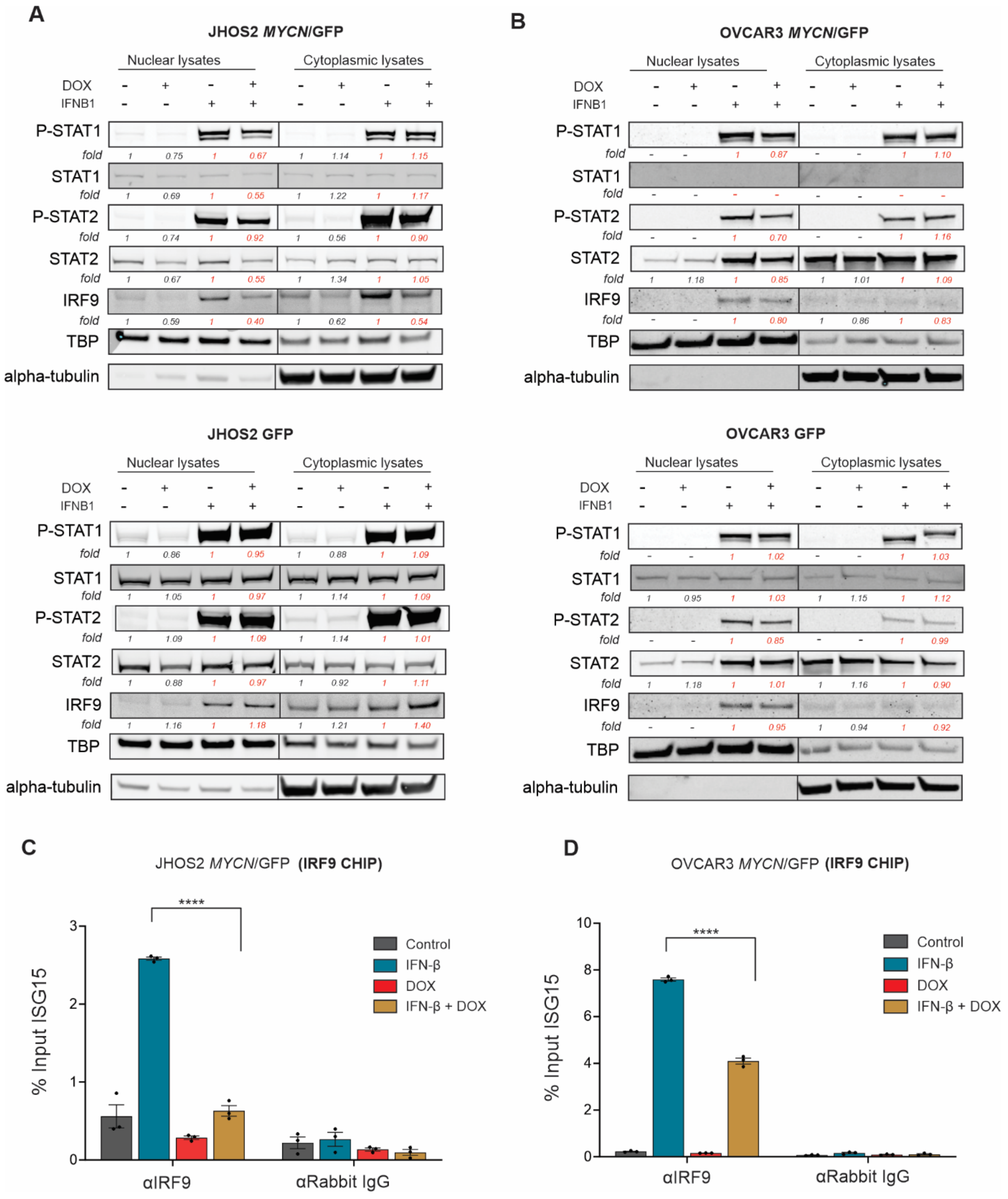

Fig. S9. N-MYC represses response to exogenous Type I IFN.

(A-B) Immunoblot of pSTAT1, STAT1, P-STAT2, STAT2, IRF9 and a-tubulin levels in JHOS2 *MYCN*/GFP and GFP controls cells (A) and OVCAR3 *MYCN*/GFP and GFP controls cells (B) pre-treated ± DOX for 72 hours and then treated with IFNB1 (10 ng/ml) for 2 hours. Nuclear and cytoplasmic lysates were prepared and subject to western blot. Paired comparisons are shown in the same color for densitometry fold changes. Data are representative of 2 independent experiments. **(C-D)** Chip-qPCR analysis of IRF9 binding to the ISRE sequence of the ISG15 promoter in JHOS2

MYCN/GFP and GFP controls cells **(C)** and OVCAR3 *MYCN*/GFP and GFP controls cells **(D)** pre-treated ± DOX for 72 hours and then treated with IFNB1 (50 ng/ml) for 30 minutes. P values were calculated using One-Way Anova with Tukey post-test for pairwise comparison. Mean ± s.e.m. of n= 3 biological replicates. *P< 0.05; **P< 0.005; ***P< 0.001; ****P< 0.0001; ns, not significant. Densitometry analysis was performed using ImageJ software.

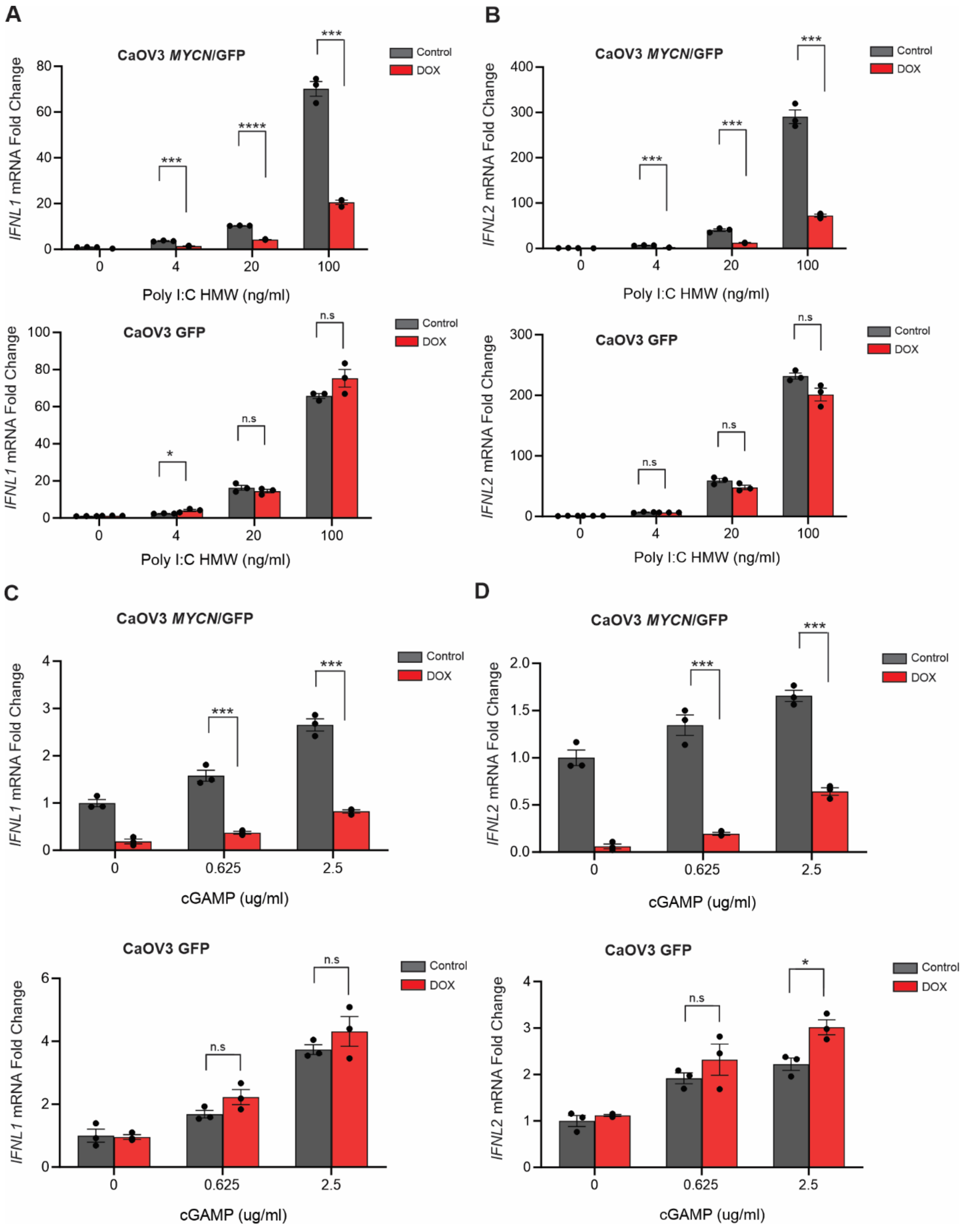

Fig. S10. N-MYC inhibits induction of IFN Type III ligands by cytosolic dsRNA and dsDNA.

(A-B) qRT-PCR of *IFNL1* **(A)** and *IFNL2* **(B)** in CaOV3 *MYCN*/GFP and GFP controls cells pre-treated ± DOX (1 ug/ml) for 72 hours and transfected with increasing concentrations of Poly I:C HMW **(B)** (0, 4, 20 and 100 ng/ml) for 24 hours. **(C-D)** qRT-PCR of *IFNL1* **(C)** and *IFNL2* **(D)** in CaOV3 *MYCN*/GFP and GFP controls cells pre-treated ± DOX (1 ug/ml) for 72 hours and transfected with increasing concentrations of 2’3’-cGAMP (0, 0.625 and 2.5 mg/ml) for 24 hours. All P values were calculated using an unpaired two-tailed Student’s t-test. Mean ± s.e.m of n=3

biological replicates shown. *P< 0.05; **P< 0.005; ***P< 0.001; ****P< 0.0001; ns, not significant.

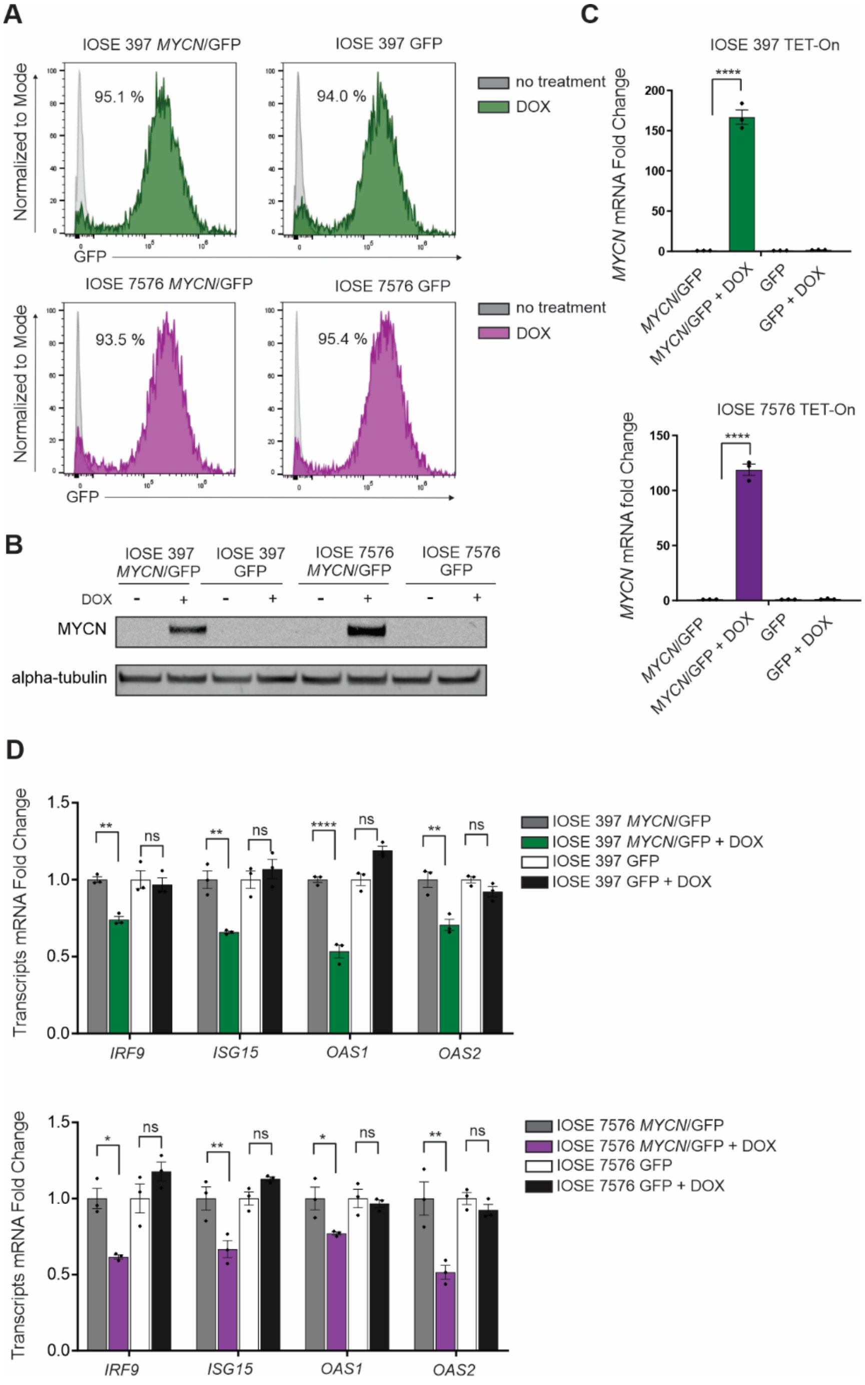

Fig. S11. ***MYCN*** **TET-On models in IOSE cell lines.**

(A) Flow cytometry quantification of GFP induction in IOSE 397 and IOSE 7576 *MYCN*/GFP and GFP controls cells pre-treated ± DOX for 72 hours. Representative fluorescence-activated cell sorting (FACS) histograms and statistical data are shown. **(B)** Immunoblot of N-MYC and alpha-tubulin in IOSE 397 and IOSE 7576 *MYCN*/GFP and GFP controls cells pre-treated ± DOX for 72 hours. Whole cell lysates (WCL) were prepared and subject to western blot. Data are representative of 2 independent experiments. **(C-D)** qRT-PCR of *MYCN* **(C)** and of ISGs (*IRF9, ISG15, OAS1* and *OAS2*) **(D)** in IOSE 397 and IOSE 7576 *MYCN*/GFP and GFP controls cells pre-treated ± DOX for 72 hours. All P values were calculated using an unpaired two-tailed Student's t-test. Mean ±

s.e.m of n=3 biological replicates shown. *P< 0.05; **P< 0.005; ***P< 0.001; ****P< 0.0001; ns, not significant.

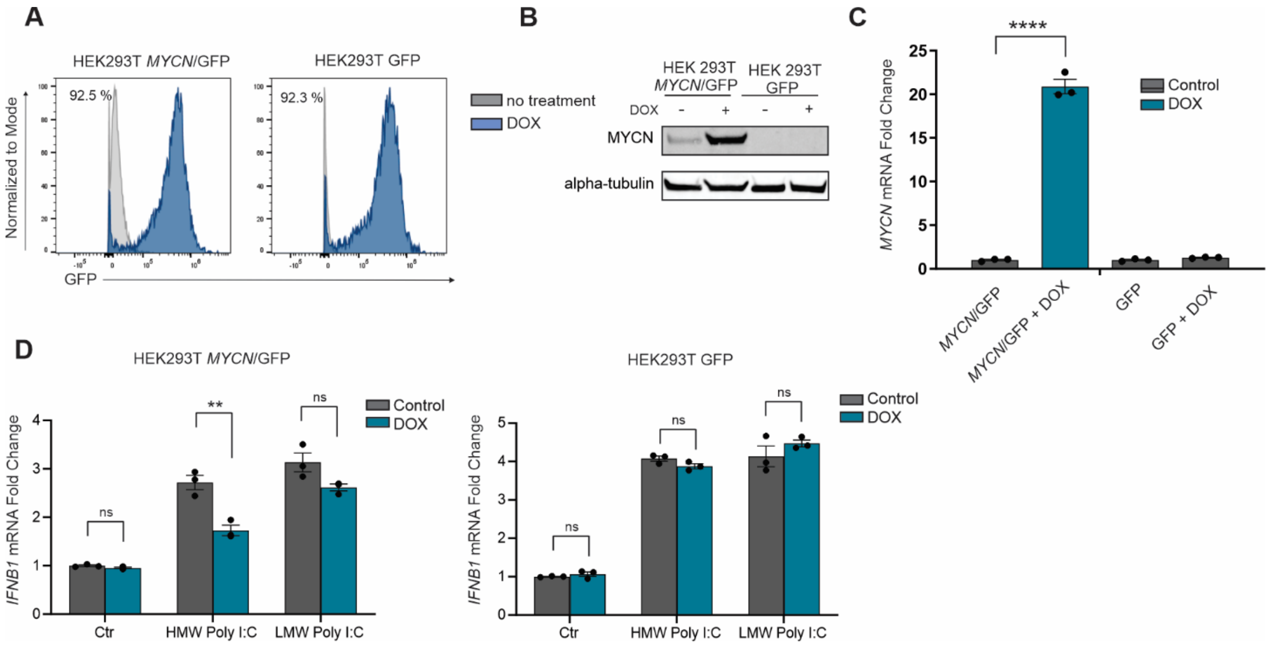

Fig. S12. **N-MYC suppress response to Poly I:C in HEK293T *MYCN* TET-ON model.**

(A) Flow cytometry quantification of GFP induction in HEK293T *MYCN*/GFP and GFP controls cells pre-treated ± DOX for 72 hours. Representative fluorescence-activated cell sorting (FACS) histograms and statistical data are shown. **(B)** Immunoblot of N-MYC and alpha-tubulin in HEK293T *MYCN*/GFP and GFP controls cells pre-treated ± DOX for 72 hours. Whole cell lysates (WCL) were prepared and subject to western blot. Data are representative of 2 independent experiments. **(C)** qRT-PCR of *MYCN* in HEK293T *MYCN*/GFP and GFP controls cells pre-treated ± DOX for 72 hours. **(D)** qRT-PCR of *IFNB1* in HEK293T *MYCN*/GFP and GFP controls cells pre-treated ± DOX for 72 hours and transfected with ± 100 ng/ml of Poly I:C HMW or ± 100 ng/ml of Poly I:C LMW for 24 hours. All P values were calculated using an unpaired two-tailed Student's t-test. Mean ± s.e.m of n=3 biological replicates shown. *P< 0.05; **P< 0.005; ***P< 0.001; ****P< 0.0001; ns, not significant

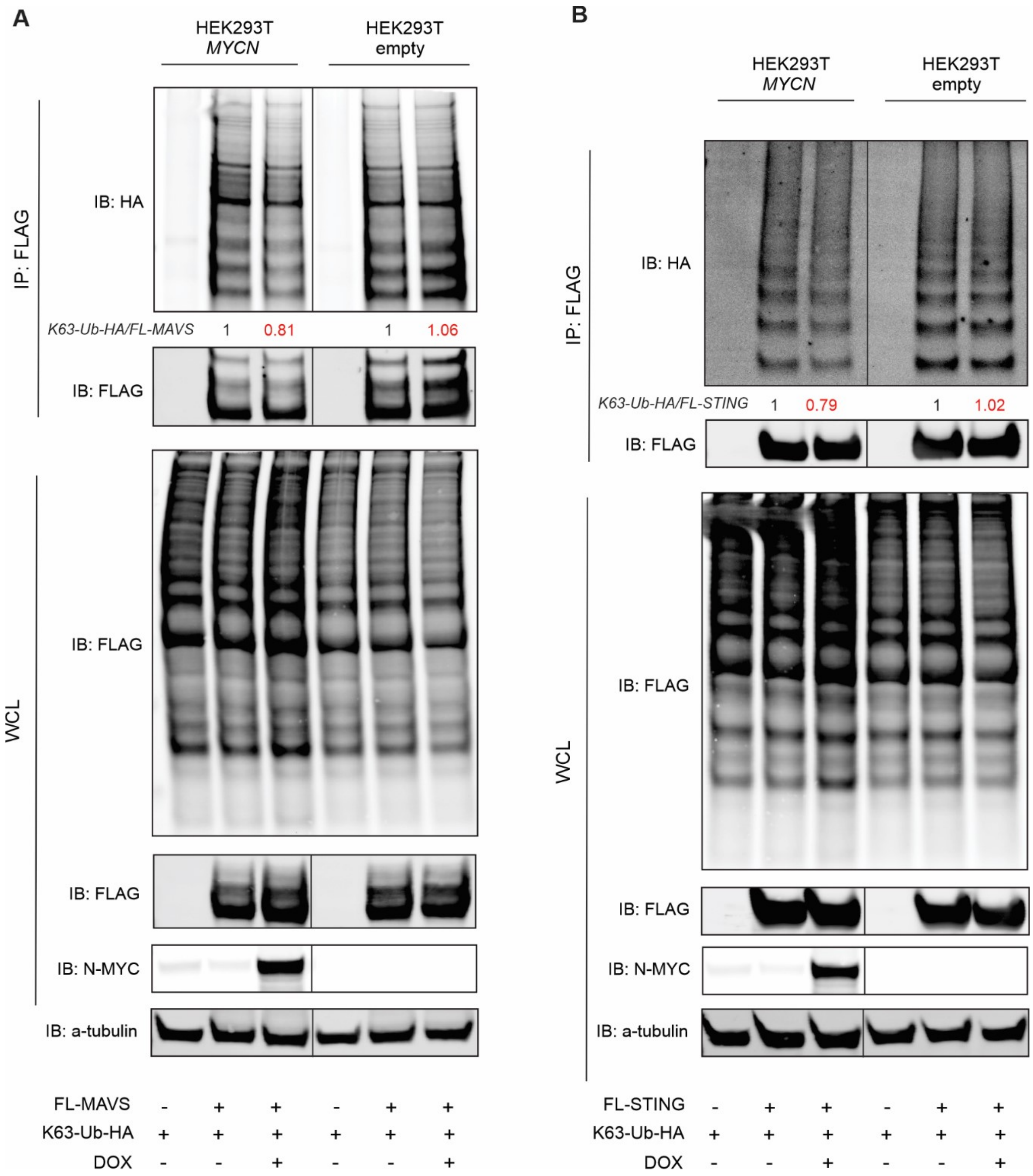

Fig. S13. N-MYC has marginal effects on K63-linked ubiquitination of MAVS and STING.

(A) HEK293T *MYCN* and Empty controls cells were pre-treated ± DOX for 72 hours and co-transfected with pFLAG-MAVS and pHA-Ub-K63 for 24 hours, lysed and immunoprecipitated with anti-FLAG conjugated magnetic beads. Immune complexes were collected and subjected to western blot and immunoblotted with the indicated antibodies. Data are representative of 2 independent experiments. **(B)** HEK293T *MYCN* and Empty controls cells were pre-treated ± DOX for 72 hours and co-transfected with pFLAG-STING and pHA-Ub-K63 for 24 hours, lysed and immunoprecipitated with anti-FLAG conjugated magnetic beads. Immune complexes were collected and subjected to western blot and immunoblotted with the indicated antibodies. Data are representative of 2 independent experiments. Paired comparisons are shown in the same color for densitometry fold changes. Densitometry analysis was performed using ImageJ software.

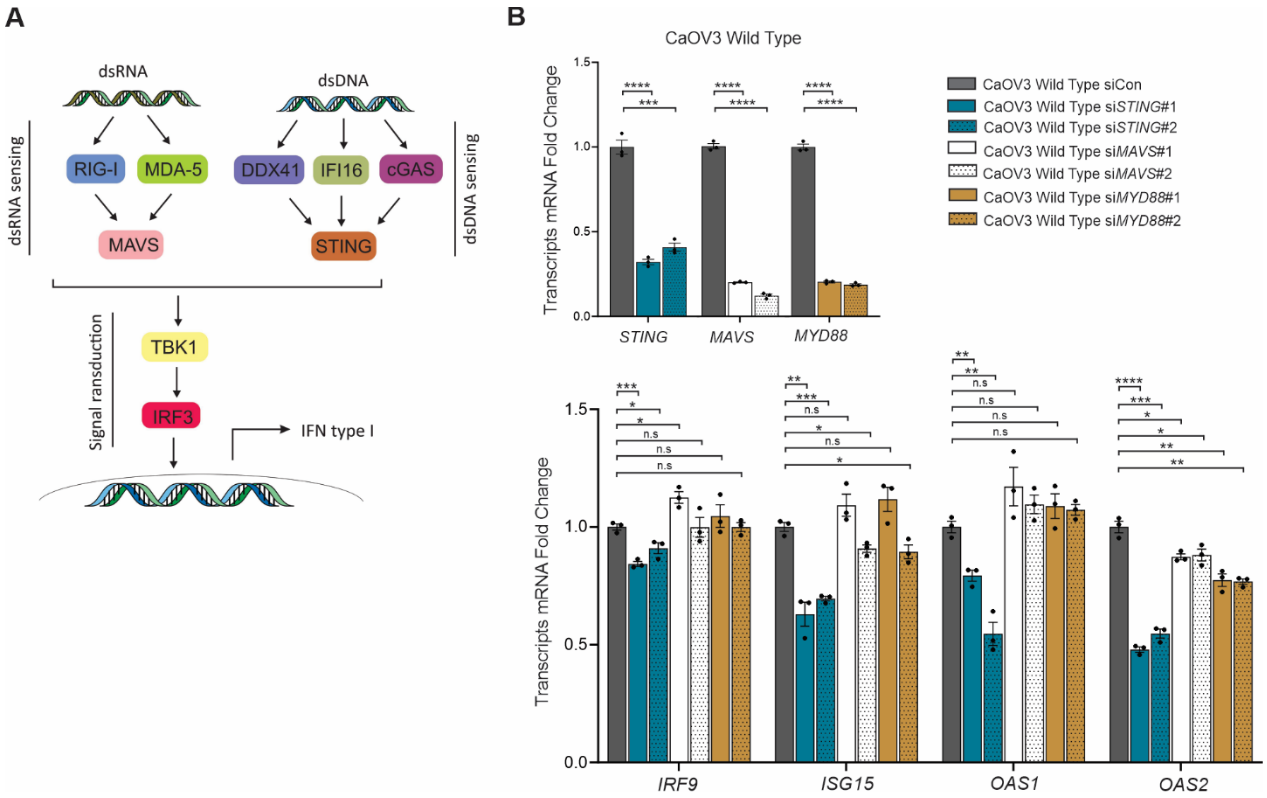

Fig. S14. Basal expression of IFN regulated genes on CaOV3 wild type cells depends on STING signaling.

(A) Schematic of dsRNA and dsDNA sensing pathways that induce IFN type 1. **(B)** qRT-PCR evaluation of *STING (TMEM173)*, *MAVS* and *MYD88* (*top*) and ISGs (*IRF9, ISG15, OAS1* and *OAS2*) (*bottom*) in CaOV3 wild type cells transfected with control siRNA or human *STING*, *MAVS* and *MYD88* siRNA for 24 hours. P values were calculated using an unpaired two-tailed Student's t-test. Mean ± s.e.m of n=3 biological replicates shown. *P< 0.05; **P< 0.005; ***P< 0.001; ****P< 0.0001; ns, not significant.

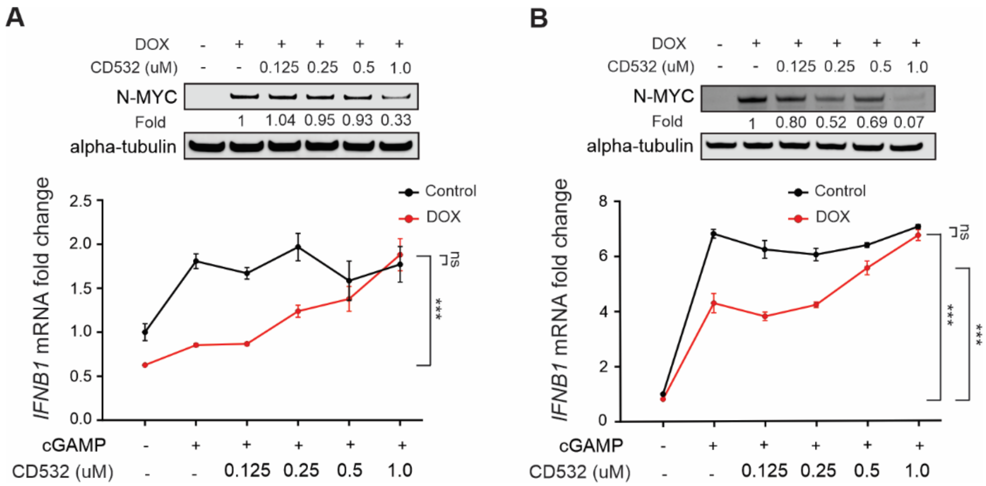

Fig. S15. Treatment with Aurora A kinase inhibitor reduced DOX-induced N-MYC protein levels and restored 2'3'-cGAMP-induced *IFNB1* mRNA expression.

(A) *Upper:* Immunoblot of N-MYC and a-tubulin in JHOS2 *MYCN*/GFP cells treated ± DOX and the indicated concentrations of the Aurora A/N-MYC inhibitor CD532. *Lower:* qRT-PCR of *IFNB1* expression in JHOS2 *MYCN*/GFP cells co-treated ± DOX and the indicated concentrations of CD532 for 72 hours, followed by transfection ± 2.5 ug/ml 2'3'-cGAMP for 24 hours. **(B)** *Upper:* Immunoblot of N-MYC and a-tubulin in OVCAR3 *MYCN*/GFP cells treated ± DOX and the indicated concentrations of the Aurora A/N-MYC inhibitor CD532. *Lower:* qRT-PCR of *IFNB1* expression in OVCAR3 *MYCN*/GFP cells co-treated ± DOX and the indicated concentrations of CD532 for 72 hours, followed by transfection ± 2.5 ug/ml 2'3'-cGAMP for 24 hours. All P values were calculated using an unpaired two-tailed Student's t-test. Mean ± s.e.m of n=3 biological replicates shown. *P< 0.05; **P< 0.005; ***P< 0.001; ****P< 0.0001; ns, not significant. Densitometry analysis was performed using ImageJ software.

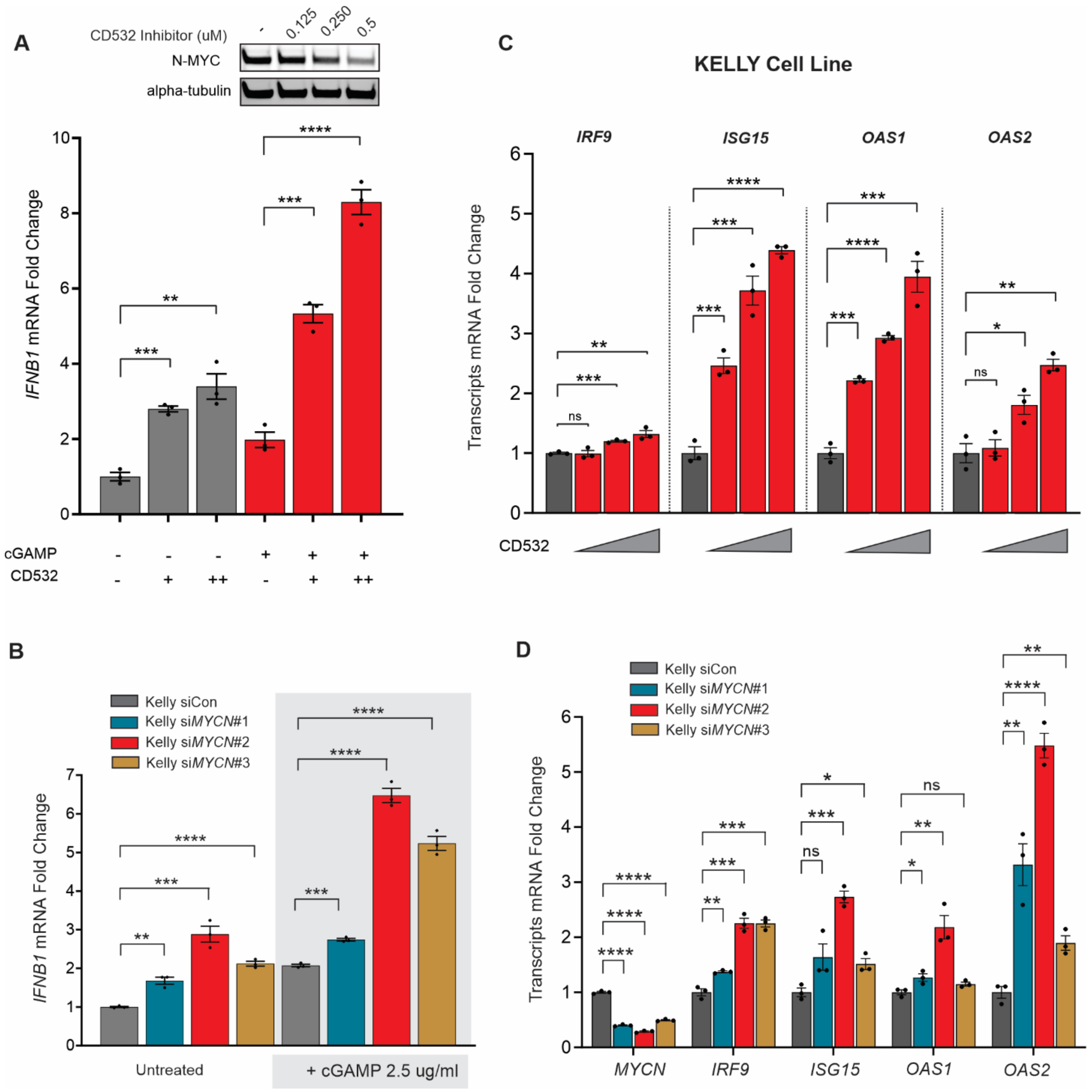

Fig. S16. Inhibition of N-MYC increases basal expression of IFN regulated genes and STING agonism in Kelly cell line.

(A) qRT-PCR of *IFNB1* in Kelly cell line treated with increasing concentrations of Aurora A/*MYCN* dual inhibitor CD532 (0, 0.125, 0., 25 and 0.5 uM) for 24 hours and transfected with ± 2.5 mg/ml of 2'3'-cGAMP for another 24 hours. **(B)** qRT-PCR of *IFNB1* in Kelly cell line transfected with control siRNA or human *MYCN* siRNAs for 48 hours and transfected with ± 2.5 mg/ml of 2'3'-cGAMP for another 24 hours. **(C)** qRT-PCR of ISGs (*IRF9, ISG15, OAS1* and *OAS2*) in Kelly cell line treated with increasing concentrations of Aurora A/*MYCN* dual inhibitor CD532 (0, 0.125, 0., 25 and 0.5 uM) for 24 hours. **(D)** qRT-PCR of *MYCN* and ISGs (*IRF9, ISG15, OAS1* and *OAS2*) in Kelly cell line transfected with control siRNA or human *MYCN* siRNAs for 48 hours. P values were calculated using an unpaired two-tailed Student's t-test. Mean ± s.e.m of n=3 biological replicates shown. *P< 0.05; **P< 0.005; ***P< 0.001; ****P< 0.0001; ns, not significant.

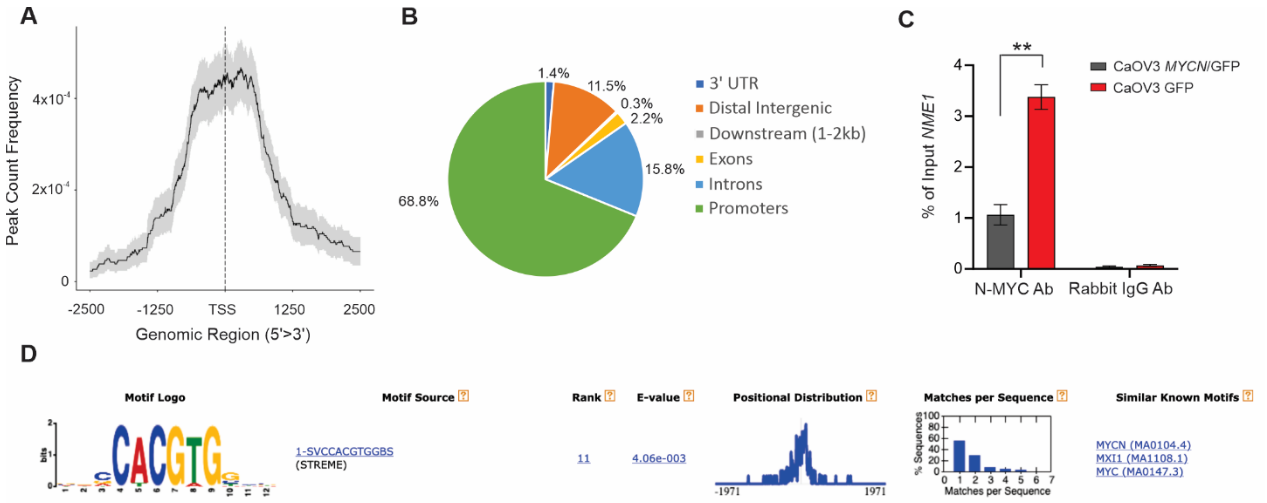

Fig. S17. N-MYC Chip-seq of CaOV3 *MYCN* TET-On cell line.

(A) Genome-wide occupancy for N-MYC as determined by ChIP-seq in CaOV3 *MYCN*/GFP cells pre-treated ± DOX for 72 hours. Comparing binding peaks between CaOV3 *MYCN* TET-ON treated or not with DOX revealed clear enrichment of peaks centred around transcription start sites following *MYCN* overexpression. The region shown is from -2.5 kb to +2.5 kb around the TSS. **(B)** Pie chart showing the genome-wide distribution of N-MYC-binding relative to gene structure in CaOV3 *MYCN*/GFP cell line pre-treated ± DOX for 72 hours. Relative ratio of N-MYC peaks at each defined genomic region versus total peaks was indicated as %. **(C)** Chip-qPCR analysis of N-MYC binding to the NME1 promoter in CaOV3 *MYCN*/GFP and GFP controls cells pre-treated ± DOX for 72 hours. **(D)** de novo motif analysis of N-MYC-binding regions in CaOV3 *MYCN*/GFP pre-treated ± DOX for 72 hours. Motif scanning of differentially-abundant peaks using STREME recovered CACGTG—the canonical N-MYC binding e-box sequence—as the most enriched motif (e-value = 0.004).

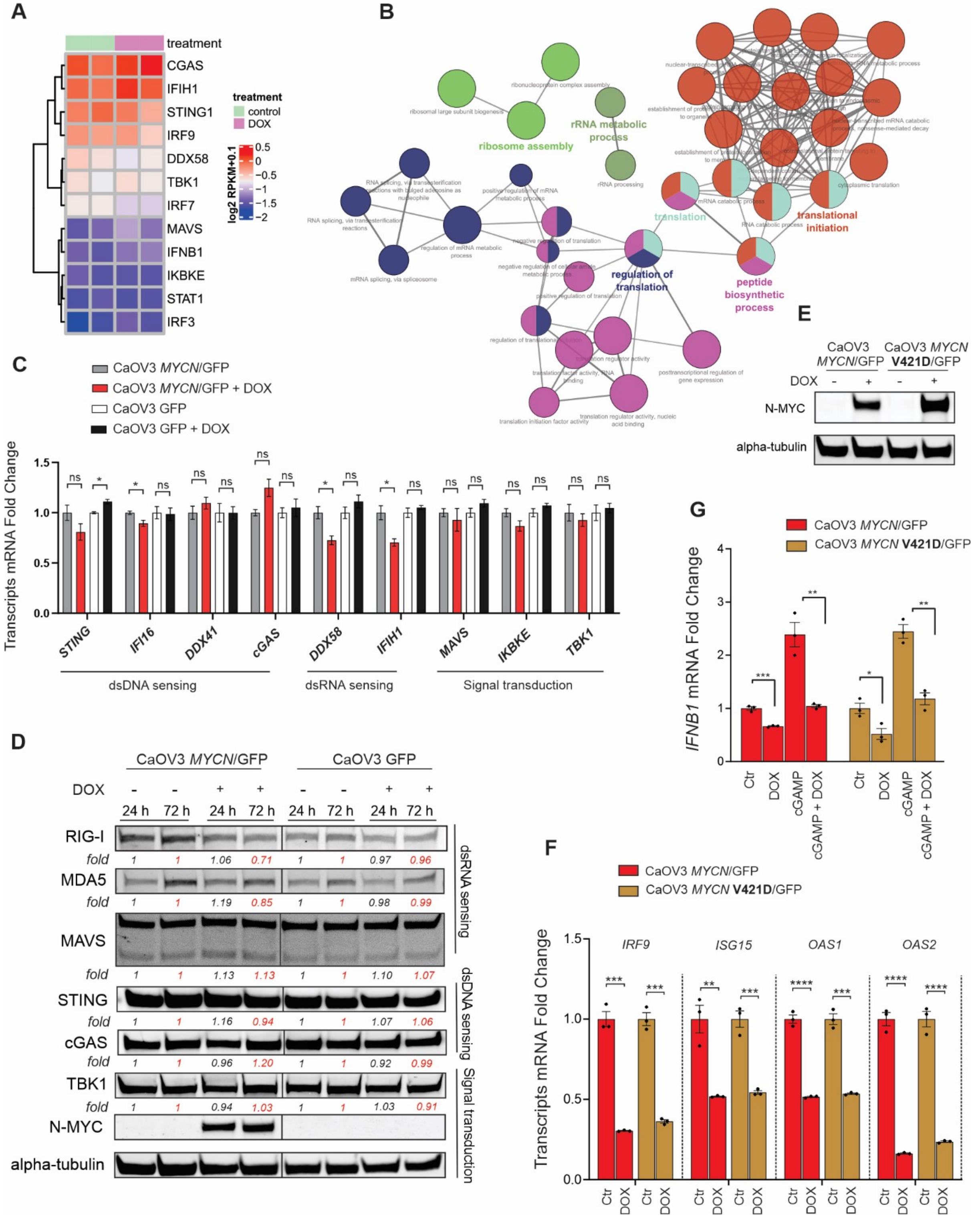

Fig. S18. N-MYC regulates basal expression of IFN regulated genes and nucleic acid sensing pathways genes by an indirect mechanism.

(A) Heatmaps represent N-MYC occupancy within promoters of innate immune signaling genes in CaOV3 *MYCN*/GFP cells pre-treated ± DOX for 72 hours. **(B)** ClueGO analysis of N-MYC bound genes. ClueGO integrates Gene Ontology (GO) terms, creating a functionally organized GO/pathway term network. The size of the nodes reflects term significance after Bonferroni

correction. Only GO terms with a p value < 0.01 were considered significant and therefore represented in the network. **(C)** qRT-PCR of *STING1 (TMEM173)*, *IFI16*, *DDX41*, *CGAS*, *DDX58*, *IFIH1*, *MAVS*, *IKBKE* and *TBK1* in CaOV3 *MYCN*/GFP and GFP controls cells pre-treated ± DOX (1 ug/ml) for 72 hours. All P values were calculated using an unpaired two-tailed Student's t-test. Mean ± s.e.m of n=3 biological replicates. *P< 0.05; **P< 0.005; ***P< 0.001; ****P< 0.0001; ns, not significant. **(D)** Immunoblot of RIG-I, MDA5, MAVS, STING, cGAS, TBK1, N-MYC and alpha-tubulin in CaOV3 *MYCN*/GFP and GFP controls cells pre-treated ± DOX for 24 or 72 hours. Whole cell lysates (WCL) were prepared and subject to western blot. Paired comparisons are shown in the same color for densitometry fold changes. Data are representative of 3 independent experiments. **(E)** Immunoblot of N-MYC and alpha-tubulin in CaOV3 *MYCN*/GFP and *MYCN*(V421D)/GFP cells pre-treated ± DOX for 72 hours. Whole cell lysates (WCL) were prepared and subject to western blot. Data are representative of 2 independent experiments. **(F)** qRT-PCR of *IRF9*, *ISG15*, *OAS1* and *OAS2* in CaOV3 *MYCN*/GFP and *MYCN*(V421D)/GFP cells pre-treated ± DOX for 72 hours. **(G)** qRT-PCR of *IFNB1* in CaOV3 *MYCN*/GFP and *MYCN*(V421D)/GFP cells pre-treated ± DOX for 72 hours and transfected with ± 2.5 mg/ml of 2'3'-cGAMP for another 24 hours. All P values were calculated using an unpaired two-tailed Student's t-test. Data in F and G are mean ± s.e.m of n=3 biological replicates. *P< 0.05; **P< 0.005; ***P< 0.001; ****P< 0.0001; ns, not significant.

A

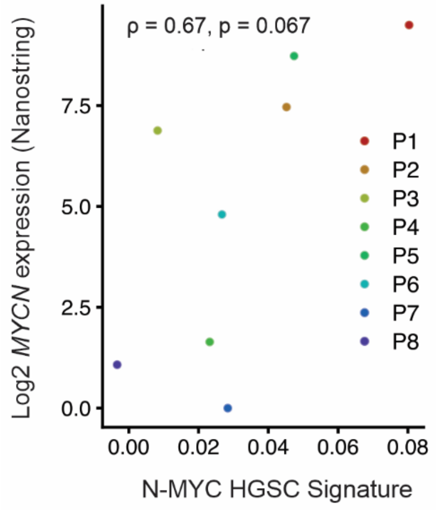

B

Correlation of N-MYC gene signature - Reactome IFN α/β signature within HGSC patients.

	ρ Values	P Values
**Patient 1**	-0.28	1.17×10^{-32}
**Patient 2**	-0.06	0.115
**Patient 3**	-0.14	5.55×10^{-11}
**Patient 4**	-0.14	4.04×10^{-4}
**Patient 5**	-0.04	3.30×10^{-3}
**Patient 6**	-0.11	7.35×10^{-5}
**Patient 7**	-0.22	2.32×10^{-7}
**Patient 8**	-0.07	0.019

ρ: Spearman's rank correlation coefficient

C

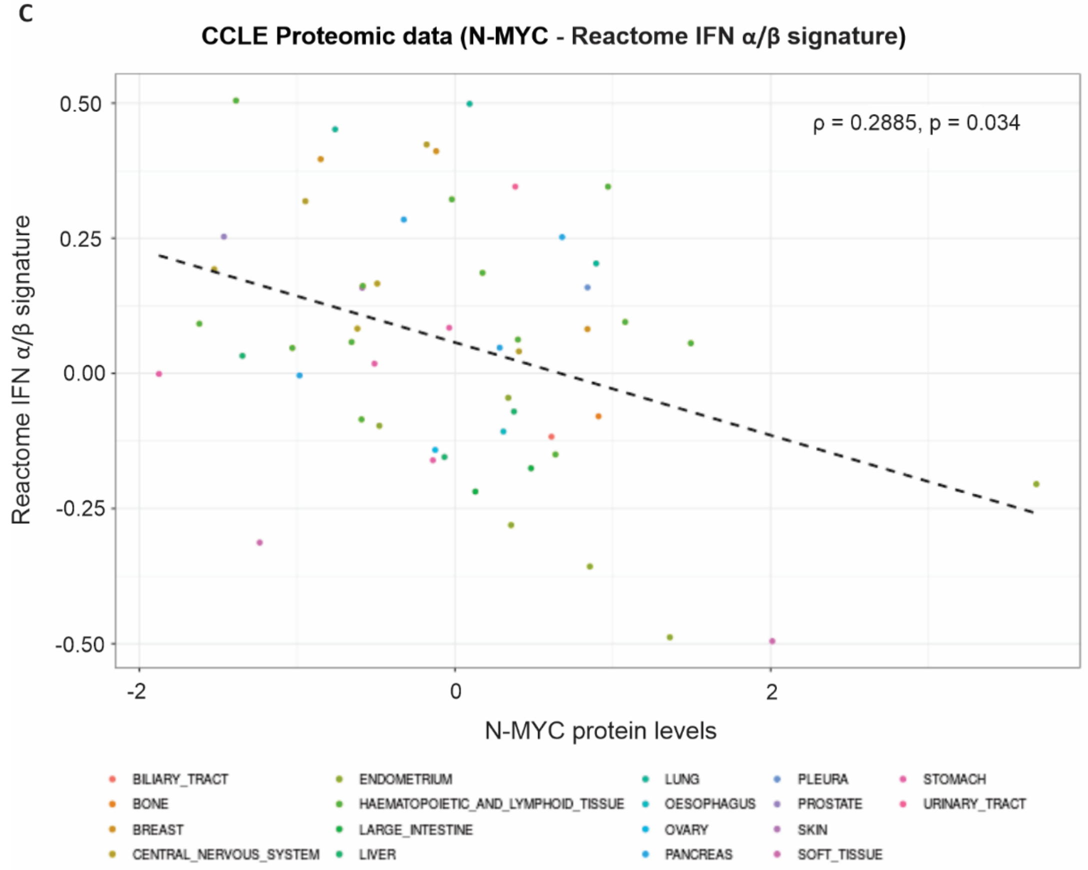

Fig. S19. ***MYCN*** **gene signature and N-MYC protein levels are negatively associated with IFN type I signaling.**

(A) Mean N-MYC HGSC gene signature (scRNA-seq CD45- compartment) and *MYCN* expression (Nanostring-FFPE whole sections) correlation across patients; N-MYC HGSC gene signature mean ± s.e.m across cells shown for each patient. **(B)** N-MYC HGSC signature score (genes identified as

up-regulated in DOX-treated CaOV3 *MYCN* TET-On cells) is negatively associated with type I IFN signaling (Reactome IFN α/β pathway) in tumor cells from 7 of 8 HGSC cancer patients. **(C)** Association of N-MYC protein levels with type I interferon signaling ($P < 0.05$; Reactome IFN α/β pathway ssGSEA) across cancer cell lines. Colored points represent different cell lines in data from the CCLE.

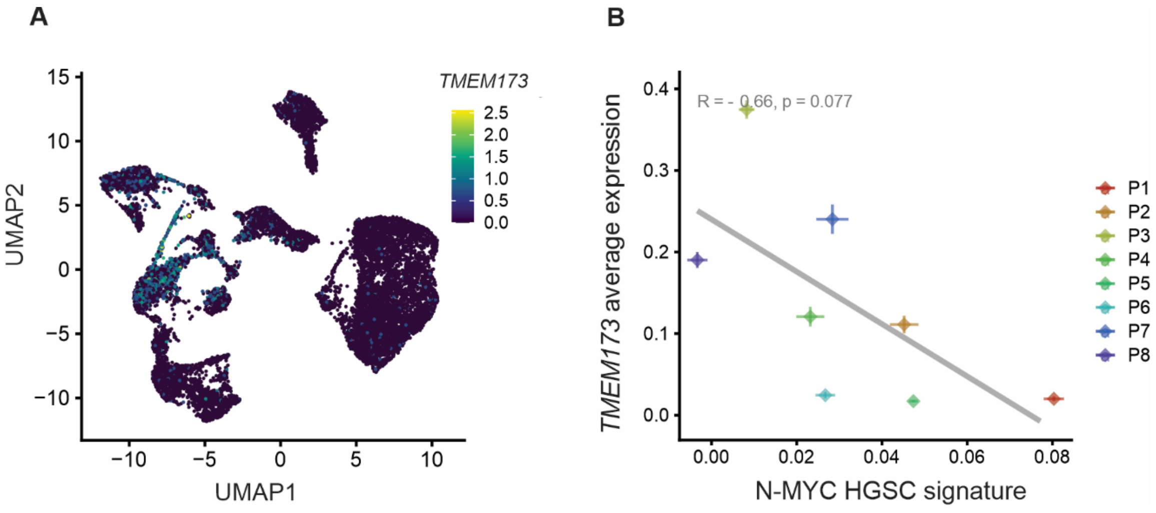

Fig. S20. *MYCN* gene signature and STING (*TMEM173*) expression in tumor cells from HGSC patients.

(A) UMAP of scRNA-seq from CD45^{-} cells (n=13,966 cells, excluding fibroblasts) from 8 HGSC cases (P1-P8). Each cell color-coded for the *TMEM173* expression computed on that cell (color scale is defined in the inset). **(B)** Mean N-MYC HGSC gene signature and mean *TMEM173* expression correlation across patients; mean ± s.e.m across cells shown for each patient (n=8). Each point represents one patient.

Table S1. Primer or oligo sequences (5'-3') used in this study.

**For qRT-PCR**		
	**Forward (5'-3')**	**Reverse (5'-3')**
human *IFNA2*	CCATTCTGGCTGTGAGGAAATA	TGATTTCTGCTCTGACAACCTC
human *IFNB1*	GCTTCTCCACTACAGCTCTTTC	CAGTATTCAAGCCTCCCATTCA
human *IFNL1*	ATCTGTCACCTTCAACCTCTTC	GTAGGGCTCAGCGCATAAATA
human *IFNL2*	CTCTGTCACCTTCAACCTCTTC	ATCTCAGGTTGCATGACTGG
human *IFNL3*	CTCTGTCACCTTCAACCTCTTC	ATCTCAGGTTGCATGACTGG
human *IFNAR1*	CACAGTGGCTCATGCCTATAA	TACCCAGGTTGGTTGATCTTG
human *IFNLR1*	GGTCACAGCCAGGATTTCTAA	CACAGGTGTGTGTCGTATGT
human *IRF3*	GCAAAGAAGGGTTGCGTTTAG	CCTGAGTTCACAAACTCGTAGAT
human *IRF5*	CCCGAGAGAAGAAGCTCATTAC	GTCTTTGAGGTCTGGGTTTGA
human *IRF7*	TCTTCGACTTCAGAGTCTTCTTC	GAAGCCCAGGTAGATGGTATAG
human *IRF1*	GTGTGGATCTTGCCACATTTC	CCGAGCAAGGCACTGTATAA
human *STAT1*	CACCTACGAACATGACCCTATC	GCTGTCTTTCCACCACAAAC
human *STAT2*	GAGGAGAAGCAATGGGTCTTAG	GGTCCACAACCAACGAATAGA
human *OAS1*	GAGGCAGCGAACTCATCTTT	CCAGCATCTTCACCGTCAG
human *OAS2*	CCAACGTGACATCCTCGATAAA	GAACCCATCAAGGGACTTCTG
human *IRF9*	CCTCTTTGTTCTTCCTGTCTCC	CACGATTATCACGGACAACTACT
human *ISG15*	GAGGCAGCGAACTCATCTTT	CCAGCATCTTCACCGTCAG
human *IFIT3*	AGTGGCTCATGCCTGTAATC	AGACGGGATTTCACTGTGTTAG
human *MB21D1*	GTATGTACCCAGAACCCTCAAG	GTCCTGAGGCACTGAAGAAA
human *DDX41*	GCCAGAGGAGATTGAGAACTATG	CACACGCTTTGTTGATGAAGG
human *DDX58*	CCATGCTGTTCTTGGGATAGT	GATGAGAGAGAGAGTGTGTGTAAAG
human *IFI16*	GCCGAACTCTCTCAATGGTATC	CTCCTCCTCCTCTCCATTCTT
human *IFIH1*	CACCATCTGCTTGGGAGAA	CCTGAAGCACGAGATGAGATAG
human *IKBKE*	CCCTGTTGGAGATGTGGAAATA	CCCTGCCAAATACAAGCAATC
human *MAVS*	CCCTGTTGGAGATGTGGAAATA	CCCTGCCAAATACAAGCAATC
human *STING*	GCCGAACTCTCTCAATGGTATC	CTCCTCCTCCTCTCCATTCTT
human *TBK1*	GAAGGGCCTCGTAGGAATAAAG	CCCGAGAAAGACTGCAAGAA
human *MYCN*	ATCCTCAAACGATGCCTTCC	CGCCTCGCTCTTTATCTTCTT
human *MYCC*	AAGCTGAGGCACACAAAGA	GCTTGGACAGGTTAGGAGTAAA
human *MYCL*	CATCAGCAACAGCACAACTATG	TCTCTCTCCAGAACCTCTTCTT
**For qPCR**		
	**Forward (5'-3')**	**Reverse (5'-3')**

human *ND1*	AACATACCCATGGCCAACCT	AGCGAAGGGTTGTAGTAGCCC
human *b-globin*	GAAGAGCCAAGGACAGGTAC	CAACTTCATCCACGTTCACC
human chromosome 3	TCAAGTGCCACATCCTATGC	ATTTTTCTAGCCAGGCACCA
human chromosome 10	ACCTGGAAATGGCTGAAATG	AAGTCCTCGCAGAGGTTTCA
human chromosome 13	CGCCAGTGTGTGTAGCACTT	TCGGCCTCTCTCAGTTCTGT
**For ChIP-qPCR**		
	**Forward (5'-3')**	**Reverse (5'-3')**
human *ISG15* promoter	GGGAAAGGGAAACCGAAACT	CCTATTATAAGCCTGAGGCACAC
human *NME1* promoter	ACAGGGCTGTGCCTTATTT	CACACACACCCGTCATTTATTC

Table S2. Dicer-substrate siRNA (DsiRNA) duplex sequences used in this study.

**DsiRNA duplex sequences**		
	**Oligo 1 (5'-3')**	**Oligo 2 (3'-5')**
human *IFNAR1* #1	AAAGCAGCACUACUUACGUCAUGGA	AAUUUCGUCGUGAUGAAUGCAGUACCU
human *IFNAR1* #2	GUCAGAAUAUUACUAGUACCAAATG	CACAGUCUUAUAAUGAUCAUGGUUUAC
human *IFNLR1* #1	AGAUUCAGGUGACUAUCCUUACCTG	GAUCUAAGUCCACUGAUAGGAAUGGAC
human *IFNLR1* #2	CUGAAGUAUGAGGUGGCAUUCUGGA	UAGACUUCAUACUCCACCGUAAGACCU
human *STING* #1	AAUCAGCAUUACAACAACCUGCUAC	UGUUAGUCGUAAUGUUGUUGGACGAUG
human *STING* #2	AUGGUCAUAUUACAUCGGAUAUCTG	CGUACCAGUAUAAUGUAGCCUAUAGAC
human *MAVS* #1	GGUGUUUCAUUGUAACCUGAAUGGA	UUCCACAAAGUAACAUUGGACUUACCU
human *MAVS* #2	CAAGUUGCCAACUAGCUCAAAGCCC	AGGUUCAACGGUUGAUCGAGUUUCGGG
human *MYD88* #1	GUAUUUAUACCUCUAAUGAAGCACA	GUCAUAAAUAUGGAGAUUACUUCGUGU
human *MYD88* #2	GCUAUCUCAACUUUCUGAUAAAACC	CUCGAUAGAGUUGAAAGACUAUUUUGG
human *MYCN* #1	GAUAUUACUUUAUCACUUUUUGAAC	AUCUAUAAUGAAAUAGUGAAAAACUUG
human *MYCN* #2	ACUUGCUAGACGCUUCUCAAAACTG	CCUGAACGAUCUGCGAAGAGUUUUGAC

Data S1. (separate file)
GSEA2 enrichment analysis table all subtypes mRNA (microarray data).

Data S2. (separate file)
GSVA enrichment analysis table in low *MYCN* tertiles.

Data S3. (separate file)
GSVA enrichment analysis table CaOV3 *MYCN*-GFP plus minus DOX

REFERENCES AND NOTES

1. D. D. Bowtell, S. Böhm, A. A. Ahmed, P.-J. Aspuria, R. C. Bast Jr, V. Beral, J. S. Berek, M. J. Birrer, S. Blagden, M. A. Bookman, J. D. Brenton, K. B. Chiappinelli, F. C. Martins, G. Coukos, R. Drapkin, R. Edmondson, C. Fotopoulou, H. Gabra, J. Galon, C. Gourley, V. Heong, D. G. Huntsman, M. Iwanicki, B. Y. Karlan, A. Kaye, E. Lengyel, D. A. Levine, K. H. Lu, I. A. McNeish, U. Menon, S. A. Narod, B. H. Nelson, K. P. Nephew, P. Pharoah, D. J. Powell Jr, P. Ramos, I. L. Romero, C. L. Scott, A. K. Sood, E. A. Stronach, F. R. Balkwill, Rethinking ovarian cancer II: Reducing mortality from high-grade serous ovarian cancer. *Nat. Rev. Cancer* **15**, 668–679 (2015).

2. L. E. Kandalaft, D. D. Laniti, G. Coukos, Immunobiology of high-grade serous ovarian cancer: Lessons for clinical translation. *Nat. Rev. Cancer* **22**, 640–656 (2022).

3. B. H. Nelson, New insights into tumor immunity revealed by the unique genetic and genomic aspects of ovarian cancer. *Curr. Opin. Immunol.* **33**, 93–100 (2015).

4. B. J. Monk, N. Colombo, A. M. Oza, K. Fujiwara, M. J. Birrer, L. Randall, E. V. Poddubskaya, G. Scambia, Y. V. Shparyk, M. C. Lim, S. M. Bhoola, J. Sohn, K. Yonemori, R. A. Stewart, X. Zhang, J. Perkins Smith, C. Linn, J. A. Ledermann, Chemotherapy with or without avelumab followed by avelumab maintenance versus chemotherapy alone in patients with previously untreated epithelial ovarian cancer (JAVELIN Ovarian 100): An open-label, randomised, phase 3 trial. *Lancet Oncol.* **22**, 1275–1289 (2021).

5. E. Pujade-Lauraine, K. Fujiwara, J. A. Ledermann, A. M. Oza, R. Kristeleit, I.-L. Ray-Coquard, G. E. Richardson, C. Sessa, K. Yonemori, S. Banerjee, A. Leary, A. V. Tinker, K. H. Jung, R. Madry, S.-Y. Park, C. K. Anderson, F. Zohren, R. A. Stewart, C. Wei, S. S. Dychter, B. J. Monk, Avelumab alone or in combination with chemotherapy versus chemotherapy alone in platinum-resistant or platinum-refractory ovarian cancer (JAVELIN Ovarian 200): An open-label, three-arm, randomised, phase 3 study. *Lancet Oncol.* **22**, 1034–1046 (2021).

6. M. Chen, S. Hu, Y. Li, T. T. Jiang, H. Jin, L. Feng, Targeting nuclear acid-mediated immunity in cancer immune checkpoint inhibitor therapies. *Signal Transduct. Target. Ther.* **5**, 270 (2020).

7. S. Heidegger, A. Wintges, F. Stritzke, S. Bek, K. Steiger, P.-A. Koenig, S. Göttert, T. Engleitner, R. Öllinger, T. Nedelko, J. C. Fischer, V. Makarov, C. Winter, R. Rad, M. R.M. van den Brink, J. Ruland, F. Bassermann, T. A. Chan, T. Haas, H. Poeck, RIG-I activation is critical for responsiveness to checkpoint blockade. *Sci. Immunol.* **4**, eaau8943 (2019).

8. H. Poeck, A. Wintges, S. Dahl, F. Bassermann, T. Haas, S. Heidegger, Tumor cell-intrinsic RIG-I signaling governs synergistic effects of immunogenic cancer therapies and checkpoint inhibitors in mice. *Eur. J. Immunol.* **51**, 1531–1534 (2021).

9. C. Lu, J. Guan, S. Lu, Q. Jin, B. Rousseau, T. Lu, D. Stephens, H. Zhang, J. Zhu, M. Yang, Z. Ren, Y. Liang, Z. Liu, C. Han, L. Liu, X. Cao, A. Zhang, J. Qiao, K. Batten, M. Chen, D. H. Castrillon, T. Wang, B. Li, L. A. Diaz Jr, G.-M. Li, Y.-X. Fu, DNA sensing in mismatch repair-deficient tumor cells is essential for anti-tumor immunity. *Cancer Cell* **39**, 96–108.e6 (2021).

10. R. Falahat, A. Berglund, R. M. Putney, P. Perez-Villarroel, S. Aoyama, S. Pilon-Thomas, G. N. Barber, J. J. Mulé, Epigenetic reprogramming of tumor cell-intrinsic STING function sculpts antigenicity and T cell recognition of melanoma. *Proc. Natl. Acad. Sci. U.S.A.* **118**, e2013598118 (2021).

11. J. C. Kim, X. Liu, K. Fitzgerald, J. S. Eng, J. Orf, S. A. O’Brien, B. Belmontes, A.-J. Casbon, S. V. Novitskiy, K. V. Tarbell, J. De Voss, J. G. Egen, Brief report: STING expressed in tumor and non-tumor compartments has distinct roles in regulating anti-tumor immunity. *Cancer Immunol. Immunother.* **72**, 1327–1335 (2023).

12. E. E. Parkes, M. P. Humphries, E. Gilmore, F. A. Sidi, V. Bingham, S. M. Phyu, S. Craig, C. Graham, J. Miller, D. Griffin, M. Salto-Tellez, S. F. Madden, R. D. Kennedy, S. F. Bakhoum, S. McQuaid, N. E. Buckley, The clinical and molecular significance associated with STING signaling in breast cancer. *NPJ Breast Cancer* **7**, 81 (2021).

13. K. A. Lawson, C. M. Sousa, X. Zhang, E. Kim, R. Akthar, J. J. Caumanns, Y. Yao, N. Mikolajewicz, C. Ross, K. R. Brown, A. A. Zid, Z. P. Fan, S. Hui, J. A. Krall, D. M. Simons, C. J. Slater, V. de Jesus, L. Tang, R. Singh, J. E. Goldford, S. Martin, Q. Huang, E. A. Francis, A. Habsid, R. Climie, D. Tieu, J. Wei, R. Li, A. H. Y. Tong, M. Aregger, K. S. Chan, H. Han, X. Wang, P. Mero, J. H. Brumell, A.

Finelli, L. Ailles, G. Bader, G. A. Smolen, G. A. Kingsbury, T. Hart, C. Kung, J. Moffat, Functional genomic landscape of cancer-intrinsic evasion of killing by T cells. *Nature* **586**, 120–126 (2020).

14. T. F. Gajewski, The next hurdle in cancer immunotherapy: Overcoming the non-T-cell-inflamed tumor microenvironment. *Semin. Oncol.* **42**, 663–671 (2015).

15. S. Spranger, T. F. Gajewski, Impact of oncogenic pathways on evasion of antitumour immune responses. *Nat. Rev. Cancer* **18**, 139–147 (2018).

16. Cancer Genome Atlas Research Network, Integrated genomic analyses of ovarian carcinoma. *Nature* **474**, 609–615 (2011).

17. Y. K. Wang, A. Bashashati, M. S. Anglesio, D. R. Cochrane, D. S. Grewal, G. Ha, A. McPherson, H. M. Horlings, J. Senz, L. M. Prentice, A. N. Karnezis, D. Lai, M. R. Aniba, A. W. Zhang, K. Shumansky, C. Siu, A. Wan, M. K. McConechy, H. Li-Chang, A. Tone, D. Provencher, M. de Ladurantaye, H. Fleury, A. Okamoto, S. Yanagida, N. Yanaihara, M. Saito, A. J. Mungall, R. Moore, M. A. Marra, C. B. Gilks, A.-M. Mes-Masson, J. N. McAlpine, S. Aparicio, D. G. Huntsman, S. P. Shah, Genomic consequences of aberrant DNA repair mechanisms stratify ovarian cancer histotypes. *Nat. Genet.* **49**, 856–865 (2017).

18. A. W. Zhang, A. McPherson, K. Milne, D. R. Kroeger, P. T. Hamilton, A. Miranda, T. Funnell, N. Little, C. P.E. de Souza, S. Laan, S. LeDoux, D. R. Cochrane, J. L.P. Lim, W. Yang, A. Roth, M. A. Smith, J. Ho, K. Tse, T. Zeng, I. Shlafman, M. R. Mayo, R. Moore, H. Failmezger, A. Heindl, Y. K. Wang, A. Bashashati, D. S. Grewal, S. D. Brown, D. Lai, A. N.C. Wan, C. B. Nielsen, C. Huebner, B. Tessier-Cloutier, M. S. Anglesio, A. Bouchard-Côté, Y. Yuan, W. W. Wasserman, C. B. Gilks, A. N. Karnezis, S. Aparicio, J. N. McAlpine, D. G. Huntsman, R. A. Holt, B. H. Nelson, S. P. Shah, Interfaces of malignant and immunologic clonal dynamics in ovarian cancer. *Cell* **173**, 1755–1769.e22 (2018).

19. I. Vázquez-García, F. Uhlitz, N. Ceglia, J. L. P. Lim, M. Wu, N. Mohibullah, J. Niyazov, A. E. B. Ruiz, K. M. Boehm, V. Bojilova, C. J. Fong, T. Funnell, D. Grewal, E. Havasov, S. Leung, A. Pasha, D. M. Patel, M. Pourmaleki, N. Rusk, H. Shi, R. Vanguri, M. J. Williams, A. W. Zhang, V. Broach, D. S. Chi, A. D. C. Paula, G. J. Gardner, S. H. Kim, M. Lennon, K. L. Roche, Y. Sonoda, O. Zivanovic, R. Kundra, A. Viale, F. N. Derakhshan, L. Geneslaw, S. I. Bhaloo, A. Maroldi, R. Nunez, F. Pareja, A.

Stylianou, M. Vahdatinia, Y. Bykov, R. N. Grisham, Y. L. Liu, Y. Lakhman, I. Nikolovski, D. Kelly, J. Gao, A. Schietinger, T. J. Hollmann, S. F. Bakhoum, R. A. Soslow, L. H. Ellenson, N. R. Abu-Rustum, C. Aghajanian, C. F. Friedman, A. M. Pherson, B. Weigelt, D. Zamarin, S. P. Shah, Ovarian cancer mutational processes drive site-specific immune evasion. *Nature* **612**, 778–786 (2022).

20. Å. Helland, M. S. Anglesio, J. George, P. A. Cowin, C. N. Johnstone, C. M. House, K. E. Sheppard, D. Etemadmoghadam, N. Melnyk, A. K. Rustgi, W. A. Phillips, H. Johnsen, R. Holm, G. B. Kristensen, M. J. Birrer; Australian Ovarian Cancer Study Group; R. B. Pearson, A.-L. Børresen-Dale, D. G. Huntsman, A. deFazio, C. J. Creighton, G. K. Smyth, D. D. L. Bowtell, Deregulation of *MYCN*, LIN28B and LET7 in a molecular subtype of aggressive high-grade serous ovarian cancers. *PLOS ONE* **6**, e18064 (2011).

21. H. Beltran, The N-myc oncogene: Maximizing its targets, regulation, and therapeutic potential. *Mol. Cancer Res.* **12**, 815–822 (2014).

22. S. Raieli, D. di Renzo, S. Lampis, C. Amadesi, L. Montemurro, A. Pession, P. Hrelia, M. Fischer, R. Tonelli, *MYCN* drives a tumor immunosuppressive environment which impacts survival in neuroblastoma. *Front. Oncol.* **11**, 625207 (2021).

23. P. Zhang, X. Wu, M. Basu, C. Dong, P. Zheng, Y. Liu, A. D. Sandler, *MYCN* amplification is associated with repressed cellular immunity in neuroblastoma: An *in silico* immunological analysis of TARGET database. *Front. Immunol.* **8**, 1473 (2017).

24. M. Schwab, *MYCN* in neuronal tumours. *Cancer Lett.* **204**, 179–187 (2004).

25. A. A. Margolin, I. Nemenman, K. Basso, C. Wiggins, G. Stolovitzky, R. D. Favera, A. Califano, ARACNE: An algorithm for the reconstruction of gene regulatory networks in a mammalian cellular context. *BMC Bioinformatics* **7**, S7 (2006).

26. B. F. Ganzfried, M. Riester, B. Haibe-Kains, T. Risch, S. Tyekucheva, I. Jazic, X. V. Wang, M. Ahmadifar, M. J. Birrer, G. Parmigiani, C. Huttenhower, L. Waldron, curatedOvarianData: Clinically annotated data for the ovarian cancer transcriptome. *Database (Oxford)* **2013**, bat013 (2013).

27. M. S. Rooney, S. A. Shukla, C. J. Wu, G. Getz, N. Hacohen, Molecular and genetic properties of tumors associated with local immune cytolytic activity. *Cell* **160**, 48–61 (2015).

28. D. Aran, M. Sirota, A. J. Butte, Systematic pan-cancer analysis of tumour purity. *Nat. Commun.* **6**, 8971 (2015).

29. E. Platanitis,D. Demiroz, A. Schneller, K. Fischer, C. Capelle, M. Hartl, T. Gossenreiter, M. Müller, M. Novatchkova, T. Decker, A molecular switch from STAT2-IRF9 to ISGF3 underlies interferon-induced gene transcription. *Nat. Commun.* **10**, 2921 (2019).

30. K. Onomoto, K. Onoguchi, M. Yoneyama, Regulation of RIG-I-like receptor-mediated signaling: Interaction between host and viral factors. *Cell. Mol. Immunol.* **18**, 539–555 (2021).

31. T. M. Karve,A. Preet, R. Sneed, C. Salamanca, X. Li, J. Xu, D. Kumar, E. M. Rosen, T. Saha, BRCA1 regulates follistatin function in ovarian cancer and human ovarian surface epithelial cells. *PLOS ONE* **7**, e37697 (2012).

32. F. Hou,L. Sun, H. Zheng, B. Skaug, Q.X. Jiang, Z. J. Chen, MAVS forms functional prion-like aggregates to activate and propagate antiviral innate immune response. *Cell* **146**, 448–461 (2011).

33. B. Liu,M. Zhang, H. Chu, H. Zhang, H. Wu, G. Song, P. Wang, K. Zhao, J. Hou, X. Wang, L. Zhang, C. Gao, The ubiquitin E3 ligase TRIM31 promotes aggregation and activation of the signaling adaptor MAVS through Lys63-linked polyubiquitination. *Nat. Immunol.* **18**, 214–224 (2017).

34. H. Liu,J. Golji, L. K. Brodeur, F. S. Chung, J. T. Chen, R. S. deBeaumont, C. P. Bullock, M. D. Jones, G. Kerr, L. Li, D. P. Rakiec, M. R. Schlabach, S. Sovath, J. D. Growney, R. A. Pagliarini, D. A. Ruddy, K. D. MacIsaac, J. M. Korn, E. R. McDonald III, Tumor-derived IFN triggers chronic pathway agonism and sensitivity to ADAR loss. *Nat. Med.* **25**, 95–102 (2019).

35. G. N. Barber, STING: Infection, inflammation and cancer. *Nat. Rev. Immunol.* **15**, 760–770 (2015).

36. M. Huang, W. A. Weiss, Neuroblastoma and *MYCN*. *Cold Spring Harb. Perspect. Med.* **3**, a014415 (2013).

37. S. Mai, J. F. Mushinski, c-Myc-induced genomic instability. *J. Environ. Pathol. Toxicol. Oncol.* **22**, 179–199 (2003).

38. K. Upton,A. Modi, K. Patel, N. M. Kendsersky, K. L. Conkrite, R. T. Sussman, G. P. Way, R. N. Adams, G. I. Sacks, P. Fortina, S. J. Diskin, J. M. Maris, J. L. Rokita, Epigenomic profiling of neuroblastoma cell lines. *Sci. Data* **7**, 116 (2020).

39. B. T. Vo,E. Wolf, D. Kawauchi, A. Gebhardt, J. E. Rehg, D. Finkelstein, S. Walz, B. L. Murphy, Y. H. Youn, Y.-G. Han, M. Eilers, M. F. Roussel, The interaction of Myc with Miz1 defines medulloblastoma subgroup identity. *Cancer Cell* **29**, 5–16 (2016).

40. Z. Sun, V. Hornung, cGAS-STING signaling. *Curr. Biol.* **32**, R730–R734 (2022).

41. H. Ishikawa, Z. Ma, G. N. Barber, STING regulates intracellular DNA-mediated, type I interferon-dependent innate immunity. *Nature* **461**, 788–792 (2009).

42. R. S. Singh,V. Vidhyasagar, S. Yang, A. B. Arna, M. Yadav, A. Aggarwal, A. N. Aguilera, S. Shinriki, K. K. Bhanumathy, K. Pandey, A. Xu, N. Rapin, M. Bosch, J. DeCoteau, J. Xiang, F. J. Vizeacoumar, Y. Zhou, V. Misra, H. Matsui, S. R. Ross, Y. Wu, DDX41 is required for cGAS-STING activation against DNA virus infection. *Cell Rep.* **39**, 110856 (2022).

43. D. R. Kroeger, K. Milne, B. H. Nelson, Tumor-infiltrating plasma cells are associated with tertiary lymphoid structures, cytolytic T-cell responses, and superior prognosis in ovarian cancer. *Clin. Cancer Res.* **22**, 3005–3015 (2016).

44. K. Sattu,F. Hochgräfe, J. Wu, G. Umapathy, C. Schönherr, K. Ruuth, D. Chand, B. Witek, J. Fuchs, P.K. Li, F. Hugosson, R. J. Daly, R. H. Palmer, B. Hallberg, Phosphoproteomic analysis of anaplastic lymphoma kinase (ALK) downstream signaling pathways identifies signal transducer and activator of transcription 3 as a functional target of activated ALK in neuroblastoma cells. *FEBS J.* **280**, 5269–5282 (2013).

45. T. Berry,W. Luther, N. Bhatnagar, Y. Jamin, E. Poon, T. Sanda, D. Pei, B. Sharma, W. R. Vetharoy, A. Hallsworth, Z. Ahmad, K. Barker, L. Moreau, H. Webber, W. Wang, Q. Liu, A. Perez-Atayde, S. Rodig, N.K. Cheung, F. Raynaud, B. Hallberg, S. P. Robinson, N. S. Gray, A. D.J. Pearson, S. A. Eccles, L. Chesler, R. E. George, The ALK(F1174L) mutation potentiates the oncogenic activity of *MYCN* in neuroblastoma. *Cancer Cell* **22**, 117–130 (2012).

46. K. Unno,Z. R. Chalmers, S. Pamarthy, R. Vatapalli, Y. Rodriguez, B. Lysy, H. Mok, V. Sagar, H. Han, Y. A. Yoo, S.-Y. Ku, H. Beltran, Y. Zhao, S. A. Abdulkadir, Activated ALK cooperates with N-Myc via Wnt/β-catenin signaling to induce neuroendocrine prostate cancer. *Cancer Res.* **81**, 2157–2170 (2021).

47. T. Matsumoto,Y. Oda, Y. Hasegawa, M. Hashimura, Y. Oguri, H. Inoue, A. Yokoi, M. Tochimoto, M. Nakagawa, Z. Jiang, M. Saegusa, Anaplastic lymphoma kinase overexpression is associated with aggressive phenotypic characteristics of ovarian high-grade serous carcinoma. *Am. J. Pathol.* **191**, 1837–1850 (2021).

48. S. Tang,F. Yang, X. du, Y. Lu, L. Zhang, X. Zhou, Aberrant expression of anaplastic lymphoma kinase in ovarian carcinoma independent of gene rearrangement. *Int. J. Gynecol. Pathol.* **35**, 337–347 (2016).

49. R. W. Jenkins,A. R. Aref, P. H. Lizotte, E. Ivanova, S. Stinson, C. W. Zhou, M. Bowden, J. Deng, H. Liu, D. Miao, M. X. He, W. Walker, G. Zhang, T. Tian, C. Cheng, Z. Wei, S. Palakurthi, M. Bittinger, H. Vitzthum, J. W. Kim, A. Merlino, M. Quinn, C. Venkataramani, J. A. Kaplan, A. Portell, P. C. Gokhale, B. Phillips, A. Smart, A. Rotem, R. E. Jones, L. Keogh, M. Anguiano, L. Stapleton, Z. Jia, M. Barzily-Rokni, I. Cañadas, T. C. Thai, M. R. Hammond, R. Vlahos, E. S. Wang, H. Zhang, S. Li, G. J. Hanna, W. Huang, M. P. Hoang, A. Piris, J.-P. Eliane, A. O. Stemmer-Rachamimov, L. Cameron, M.-J. Su, P. Shah, B. Izar, M. Thakuria, N. R. LeBoeuf, G. Rabinowits, V. Gunda, S. Parangi, J. M. Cleary, B. C. Miller, S. Kitajima, R. Thummalapalli, B. Miao, T. U. Barbie, V. Sivathanu, J. Wong, W. G. Richards, R. Bueno, C. H. Yoon, J. Miret, M. Herlyn, L. A. Garraway, E. M. van Allen, G. J. Freeman, P. T. Kirschmeier, J. H. Lorch, P. A. Ott, F. S. Hodi, K. T. Flaherty, R. D. Kamm, G. M. Boland, K.-K. Wong, D. Dornan, C. P. Paweletz, D. A. Barbie, Ex vivo profiling of PD-1 blockade using organotypic tumor spheroids. *Cancer Discov.* **8**, 196–215 (2018).

50. F. Coscia,K. M. Watters, M. Curtis, M. A. Eckert, C. Y. Chiang, S. Tyanova, A. Montag, R. R. Lastra, E. Lengyel, M. Mann, Integrative proteomic profiling of ovarian cancer cell lines reveals precursor cell associated proteins and functional status. *Nat. Commun.* **7**, 12645 (2016).

51. S. Domcke, R. Sinha, D. A. Levine, C. Sander, N. Schultz, Evaluating cell lines as tumour models by comparison of genomic profiles. *Nat. Commun.* **4**, 2126 (2013).

52. S. Kitajima,E. Ivanova, S. Guo, R. Yoshida, M. Campisi, S. K. Sundararaman, S. Tange, Y. Mitsuishi, T. C. Thai, S. Masuda, B. P. Piel, L. M. Sholl, P. T. Kirschmeier, C. P. Paweletz, H. Watanabe, M. Yajima, D. A. Barbie, Suppression of STING associated with LKB1 loss in KRAS-driven lung cancer. *Cancer Discov.* **9**, 34–45 (2019).

53. M. Ghosh,S. Saha, J. Bettke, R. Nagar, A. Parrales, T. Iwakuma, A. W.M. van der Velden, L. A. Martinez, Mutant p53 suppresses innate immune signaling to promote tumorigenesis. *Cancer Cell* **39**, 494–508.e5 (2021).

54. F. Meng,Z. Yu, D. Zhang, S. Chen, H. Guan, R. Zhou, Q. Wu, Q. Zhang, S. Liu, M. K. Venkat Ramani, B. Yang, X.-Q. Ba, J. Zhang, J. Huang, X. Bai, J. Qin, X.-H. Feng, S. Ouyang, Y. J. Zhang, T. Liang, P. Xu, Induced phase separation of mutant NF2 imprisons the cGAS-STING machinery to abrogate antitumor immunity. *Mol. Cell* **81**, 4147–4164.e7 (2021).

55. T. Xia, H. Konno, G. N. Barber, Recurrent loss of STING signaling in melanoma correlates with susceptibility to viral oncolysis. *Cancer Res.* **76**, 6747–6759 (2016).

56. N. de Queiroz, T. Xia, H. Konno, G. N. Barber, Ovarian cancer cells commonly exhibit defective STING signaling which affects sensitivity to viral oncolysis. *Mol. Cancer Res.* **17**, 974–986 (2019).

57. S. Liu,X. Cai, J. Wu, Q. Cong, X. Chen, T. Li, F. du, J. Ren, Y.T. Wu, N. V. Grishin, Z. J. Chen, Phosphorylation of innate immune adaptor proteins MAVS, STING, and TRIF induces IRF3 activation. *Science* **347**, aaa2630 (2015).

58. H. Alborzinia,A. F. Flórez, S. Kreth, L. M. Brückner, U. Yildiz, M. Gartlgruber, D. I. Odoni, G. Poschet, K. Garbowicz, C. Shao, C. Klein, J. Meier, P. Zeisberger, M. Nadler-Holly, M. Ziehm, F. Paul, J. Burhenne, E. Bell, M. Shaikhkarami, R. Würth, S. A. Stainczyk, E. M. Wecht, J. Kreth, M. Büttner, N. Ishaque, M. Schlesner, B. Nicke, C. Stresemann, M. Llamazares-Prada, J. H. Reiling, M. Fischer, I. Amit, M. Selbach, C. Herrmann, S. Wölfl, K.-O. Henrich, T. Höfer, A. Trumpp, F. Westermann, *MYCN* mediates cysteine addiction and sensitizes neuroblastoma to ferroptosis. *Nat. Cancer* **3**, 471–485 (2022).

59. Y. Lu,Q. Yang, Y. Su, Y. Ji, G. Li, X. Yang, L. Xu, Z. Lu, J. Dong, Y. Wu, J.-X. Bei, C. Pan, X. Gu, B. Li, *MYCN* mediates TFRC-dependent ferroptosis and reveals vulnerabilities in neuroblastoma. *Cell Death Dis.* **12**, 511 (2021).

60. M. Jia,D. Qin, C. Zhao, L. Chai, Z. Yu, W. Wang, L. Tong, L. Lv, Y. Wang, J. Rehwinkel, J. Yu, W. Zhao, Redox homeostasis maintained by GPX4 facilitates STING activation. *Nat. Immunol.* **21**, 727–735 (2020).

61. M. J. White,K. McArthur, D. Metcalf, R. M. Lane, J. C. Cambier, M. J. Herold, M. F. van Delft, S. Bedoui, G. Lessene, M. E. Ritchie, D. C.S. Huang, B. T. Kile, Apoptotic caspases suppress mtDNA-induced STING-mediated type I IFN production. *Cell* **159**, 1549–1562 (2014).

62. A. P. West,W. Khoury-Hanold, M. Staron, M. C. Tal, C. M. Pineda, S. M. Lang, M. Bestwick, B. A. Duguay, N. Raimundo, D. A. MacDuff, S. M. Kaech, J. R. Smiley, R. E. Means, A. Iwasaki, G. S. Shadel, Mitochondrial DNA stress primes the antiviral innate immune response. *Nature* **520**, 553–557 (2015).

63. A. I. Nieminen, J. I. Partanen, A. Hau, J. Klefstrom, c-Myc primed mitochondria determine cellular sensitivity to TRAIL-induced apoptosis. *EMBO J.* **26**, 1055–1067 (2007).

64. J. S. Riley,G. Quarato, C. Cloix, J. Lopez, J. O'Prey, M. Pearson, J. Chapman, H. Sesaki, L. M. Carlin, J. F. Passos, A. P. Wheeler, A. Oberst, K. M. Ryan, S. W.G. Tait, Mitochondrial inner membrane permeabilisation enables mtDNA release during apoptosis. *EMBO J.* **37**, e99238 (2018).

65. F. Li,Y. Wang, K. I. Zeller, J. J. Potter, D. R. Wonsey, K. A. O'Donnell, J.-W. Kim, J. T. Yustein, L. A. Lee, C. V. Dang, Myc stimulates nuclearly encoded mitochondrial genes and mitochondrial biogenesis. *Mol. Cell. Biol.* **25**, 6225–6234 (2005).

66. Y. G. Chen, S. Hur, Cellular origins of dsRNA, their recognition and consequences. *Nat. Rev. Mol. Cell Biol.* **23**, 286–301 (2022).

67. E. A. Bowling,J. H. Wang, F. Gong, W. Wu, N. J. Neill, I. S. Kim, S. Tyagi, M. Orellana, S. J. Kurley, R. Dominguez-Vidaña, H.-C. Chung, T. Y.-T. Hsu, J. Dubrulle, A. B. Saltzman, H. Li, J. K. Meena, G. M. Canlas, S. Chamakuri, S. Singh, L. M. Simon, C. M. Olson, L. E. Dobrolecki, M. T. Lewis, B.

Zhang, I. Golding, J. M. Rosen, D. W. Young, A. Malovannaya, F. Stossi, G. Miles, M. J. Ellis, L. Yu, S. Buonamici, C. Y. Lin, K. L. Karlin, X. H.-F. Zhang, T. F. Westbrook, Spliceosome-targeted therapies trigger an antiviral immune response in triple-negative breast cancer. *Cell* **184**, 384–403.e21 (2021).

68. S. Gomez,O. L. Cox, R. R. Walker III, U. Rentia, M. Hadley, E. Arthofer, N. Diab, E. E. Grundy, T. Kanholm, J. I. McDonald, J. Kobyra, E. Palmer, S. Noonepalle, A. Villagra, D. Leitenberg, C. M. Bollard, Y. Saunthararajah, K. B. Chiappinelli, Inhibiting DNA methylation and RNA editing upregulates immunogenic RNA to transform the tumor microenvironment and prolong survival in ovarian cancer. *J. Immunother. Cancer* **10**, e004974 (2022).

69. D. Zimmerli,C. S. Brambillasca, F. Talens, J. Bhin, R. Linstra, L. Romanens, A. Bhattacharya, S. E. P. Joosten, A. M. da Silva, N. Padrao, M. D. Wellenstein, K. Kersten, M. de Boo, M. Roorda, L. Henneman, R. de Bruijn, S. Annunziato, E. van der Burg, A. P. Drenth, C. Lutz, T. Endres, M. van de Ven, M. Eilers, L. Wessels, K. E. de Visser, W. Zwart, R. S. N. Fehrmann, M. A. T. M. van Vugt, J. Jonkers, MYC promotes immune-suppression in triple-negative breast cancer via inhibition of interferon signaling. *Nat. Commun.* **13**, 6579 (2022).

70. N. Muthalagu,T. Monteverde, X. Raffo-Iraolagoitia, R. Wiesheu, D. Whyte, A. Hedley, S. Laing, B. Kruspig, R. Upstill-Goddard, R. Shaw, S. Neidler, C. Rink, S. A. Karim, K. Gyuraszova, C. Nixon, W. Clark, A. V. Biankin, L. M. Carlin, S. B. Coffelt, O. J. Sansom, J. P. Morton, D. J. Murphy, Repression of the type I interferon pathway underlies MYC- and KRAS-dependent evasion of NK and B cells in pancreatic ductal adenocarcinoma. *Cancer Discov.* **10**, 872–887 (2020).

71. R. Dhanasekaran,A. Deutzmann, W. D. Mahauad-Fernandez, A. S. Hansen, A. M. Gouw, D. W. Felsher, The MYC oncogene—The grand orchestrator of cancer growth and immune evasion. *Nat. Rev. Clin. Oncol.* **19**, 23–36 (2022).

72. S. Swaminathan,A. S. Hansen, L. D. Heftdal, R. Dhanasekaran, A. Deutzmann, W. D. M. Fernandez, D. F. Liefwalker, C. Horton, A. Mosley, M. Liebersbach, H. T. Maecker, D. W. Felsher, MYC functions as a switch for natural killer cell-mediated immune surveillance of lymphoid malignancies. *Nat. Commun.* **11**, 2860 (2020).

73. S.-Y. Wu,Y. Xiao, J.-L. Wei, X.-E. Xu, X. Jin, X. Hu, D.-Q. Li, Y.-Z. Jiang, Z.-M. Shao, MYC suppresses STING-dependent innate immunity by transcriptionally upregulating DNMT1 in triple-negative breast cancer. *J. Immunother. Cancer* **9**, e002528 (2021).

74. S. Breit, M. Schwab, Suppression of MYC by high expression of NMYC in human neuroblastoma cells. *J. Neurosci. Res.* **24**, 21–28 (1989).

75. S. M. McWhirter, C. A. Jefferies, Nucleic acid sensors as therapeutic targets for human disease. *Immunity* **53**, 78–97 (2020).

76. C. Vanpouille-Box, J. A. Hoffmann, L. Galluzzi, Pharmacological modulation of nucleic acid sensors - therapeutic potential and persisting obstacles. *Nat. Rev. Drug Discov.* **18**, 845–867 (2019).

77. B. Qiu, K. K. Matthay, Advancing therapy for neuroblastoma. *Nat. Rev. Clin. Oncol.* **19**, 515–533 (2022).

78. A. Takahashi,T. M. Loo, R. Okada, F. Kamachi, Y. Watanabe, M. Wakita, S. Watanabe, S. Kawamoto, K. Miyata, G. N. Barber, N. Ohtani, E. Hara, Downregulation of cytoplasmic DNases is implicated in cytoplasmic DNA accumulation and SASP in senescent cells. *Nat. Commun.* **9**, 1249 (2018).

79. T. L. Bailey, STREME: Accurate and versatile sequence motif discovery. *Bioinformatics* **37**, 2834–2840 (2021).

80. G. Bindea,B. Mlecnik, H. Hackl, P. Charoentong, M. Tosolini, A. Kirilovsky, W.H. Fridman, F. Pagès, Z. Trajanoski, J. Galon, ClueGO: A cytoscape plug-in to decipher functionally grouped gene ontology and pathway annotation networks. *Bioinformatics* **25**, 1091–1093 (2009).

81. S. Durinck, P. T. Spellman, E. Birney, W. Huber, Mapping identifiers for the integration of genomic datasets with the R/Bioconductor package biomaRt. *Nat. Protoc.* **4**, 1184–1191 (2009).

82. M. S. Carro,W. K. Lim, M. J. Alvarez, R. J. Bollo, X. Zhao, E. Y. Snyder, E. P. Sulman, S. L. Anne, F. Doetsch, H. Colman, A. Lasorella, K. Aldape, A. Califano, A. Iavarone, The transcriptional network for mesenchymal transformation of brain tumours. *Nature* **463**, 318–325 (2010).

83. W. Luo, M. S. Friedman, K. Shedden, K. D. Hankenson, P. J. Woolf, GAGE: Generally applicable gene set enrichment for pathway analysis. *BMC Bioinformatics* **10**, 161 (2009).

84. Maxime, RVAideMemoire: Testing and plotting procedures for biostatistics. In: R package version 0.9-81-2 (2022).

85. W. Viechtbauer, Conducting meta-analyses in R with the metafor package. *J. Stat. Softw.* **36**, 1–48 (2010).

86. D. P. Nusinow,J. Szpyt, M. Ghandi, C. M. Rose, E. R. McDonald III, M. Kalocsay, J. Jané-Valbuena, E. Gelfand, D. K. Schweppe, M. Jedrychowski, J. Golji, D. A. Porter, T. Rejtar, Y. K. Wang, G. V. Kryukov, F. Stegmeier, B. K. Erickson, L. A. Garraway, W. R. Sellers, S. P. Gygi, Quantitative proteomics of the cancer cell line encyclopedia. *Cell* **180**, 387–402.e16 (2020).

87. S. Hanzelmann, R. Castelo, J. Guinney, GSVA: Gene set variation analysis for microarray and RNA-seq data. *BMC Bioinformatics* **14**, 7 (2013).
